# Adaptation to seasonal drought in *Arabis alpina* is linked to the demographic history and climatic changes since the last glacial maximum

**DOI:** 10.1093/molbev/msag212

**Published:** 2026-08-22

**Authors:** Bastiaan Tjeng, Mehak Mehak, Helene Bråten Grindeland, Jasmin Zohren, Andrea Dalla Libera, Jörg Wunder, Carlos Alonso-Blanco, George Coupland, Andrea Fulgione

**Affiliations:** Department of Plant Developmental Biology, Max Planck Institute for Plant Breeding Research, Carl-von-Linne-Weg 10, Cologne 50829, Germany; Department of Plant Developmental Biology, Max Planck Institute for Plant Breeding Research, Carl-von-Linne-Weg 10, Cologne 50829, Germany; Department of Plant Developmental Biology, Max Planck Institute for Plant Breeding Research, Carl-von-Linne-Weg 10, Cologne 50829, Germany; Department of Plant Developmental Biology, Max Planck Institute for Plant Breeding Research, Carl-von-Linne-Weg 10, Cologne 50829, Germany; Department of Plant Developmental Biology, Max Planck Institute for Plant Breeding Research, Carl-von-Linne-Weg 10, Cologne 50829, Germany; Department Digitalization 2, Nunhems Netherlands BV, Napoleonsweg 152, AB Nunhem 6083, The Netherlands; Centro Nacional de Biotecnologia (CNB), Consejo Superior de Investigaciones Científicas (CSIC), Madrid 28049, Spain; Department of Plant Developmental Biology, Max Planck Institute for Plant Breeding Research, Carl-von-Linne-Weg 10, Cologne 50829, Germany; Department of Plant Developmental Biology, Max Planck Institute for Plant Breeding Research, Carl-von-Linne-Weg 10, Cologne 50829, Germany

**Keywords:** plant adaptation, population genetics, demographic history, drought adaptation

## Abstract

Understanding how species adapt to new environments is a central goal in evolutionary biology, and a topical question in climate change research. Here, we sequenced the genomes of 426 individuals of the perennial, Arctic-alpine herb *Arabis alpina* to study demography and adaptation, with a focus on populations in Northern Spain, that experience warm and dry summers. Our inference supports a scenario in which *A. alpina* colonized Northern Spain in a range expansion event that started near the Alps around 216 thousand years ago (kya). During the last glacial episode (115 to 12 kya), this expansion proceeded westward, and effective population sizes were large across Europe, likely due to a larger suitable habitat for *A. alpina*. These ancient demographic events gave rise to a highly diverged genetic lineage in Northern Spain. In the present interglacial (between 12 kya and present), populations became increasingly fragmented, and lost genetic diversity across Europe. Furthermore, we detected signatures of selection at genes associated with responses to abiotic stress, including drought stress, and regulation of growth, for instance at *SC5D* and *NAC055*, which reflects the climatic changes since the last glacial period. Notably, an ancient polymorphism at the gene *FRL1* emerged as a candidate for conferring variation in flowering behavior, and for contributing to adaptation to drought. Our study suggests that the combination of ancestral variation in flowering behavior, and positive selection on new mutations involved in drought responses, underlies the evolution of a new trait syndrome, and adaptation to climate change.

## Introduction

Understanding how species shifted and expanded their range through time, and whether and how they can adapt to new environments are central goals of evolutionary biology, and topical questions in climate change research (Waldvogel et al. 2020; Aguirre-Liguori et al. 2021; Hancock et al. 2025). In the current Quaternary period (2.6 million years ago to present), species experienced repeated glacial–interglacial cycles that influenced their distribution range and pattern of genomic variation (Hewitt 2000; Petit et al. 2003). As species move around the landscape, they can encounter new environmental conditions, they can adapt, and colonize new habitats (Davis and Shaw 2001; Luqman et al. 2023). Depending on the genetic architecture of the traits under selection, population genetic models describe how adaptation can happen through a rapid increase in population frequency of a beneficial allele (a selective sweep) from single or recurrent *de novo* mutations, or from standing genetic variation, or through infinitesimal allele frequency shifts at many loci (Stephan 2016). However, a particularly challenging scenario is when multiple environmental conditions change simultaneously, which can result in selective pressures acting on several traits. For instance, current climate change is characterized by simultaneous changes in average temperature, seasonality, precipitation, and in climatic extremes such as heatwaves, storms, and droughts (Calvin et al. 2023).

In this scenario, adaptation may require the evolution of new combinations of trait values (new trait syndromes), which may be considerably more complex than a selective sweep. If adaptation proceeds from new genetic variants that influence variation at multiple traits, the waiting time may be long. Alternatively, adaptation might proceed from standing genetic variation. In this case, the covariance structure among traits, shaped by the past history of a population, may align or misalign with multivariate selection, which can respectively increase or constrain the potential for adaptation (Schluter 1996; Etterson and Shaw 2001; Kirkpatrick 2009; Lovell et al. 2013). With multivariate selective pressures, it is unclear which dynamics would lead to adaptation in natural populations.

An interesting case of adaptive trait covariance, is when species segregate for alternative seasonal strategies (Moran et al. 2016). For instance, in annual plants like *Arabidopsis thaliana*, traits affecting life history, such as the timing of germination (partly regulated by seed dormancy) and of flowering onset, growth rates, and stress responses underlie local adaptation and often co-vary (Ludlow 1989; Donohue 2002; Mckay et al. 2003; Alonso-Blanco et al. 2009; Debieu et al. 2013; Lovell et al. 2013; Kooyers 2015; Glander et al. 2018; Vasseur et al. 2018; Takou et al. 2019). Correlations among these traits create alternative, seasonal life-history strategies, such as the winter annual and the spring (or summer) annual habits, which segregate within species (Laibach 1951; Rédei 1970).

In winter annuals, early flowering is inhibited by the action of *FLOWERING LOCUS C (FLC)*, which is upregulated by *FRIGIDA (FRI)*, until flowering is induced by a prolonged period of cold temperatures (vernalization) (Andrés and Coupland 2012).

The spring annual habit is associated with multiple nonsense mutations at *FRI*, and in rare cases at *FLC*, which impair the vernalization pathway and accelerate flowering (Johanson et al. 2000; Stinchcombe et al. 2004; Méndez-Vigo et al. 2011; Zhang and Jiménez-Gómez 2020; Fulgione et al. 2022). This habit tends to occur in disturbed, ruderal landscapes, and in Mediterranean climates, where the ability to set seeds before the onset of drought or disturbance enables an escape strategy (Ludlow 1989; Mckay et al. 2003; Franks et al. 2007; Kooyers 2015).

The adaptive value of seasonal strategies differs in perennial plants, which account for about 90% of all plants (The Angiosperm Phylogeny Group 2016). In perennials, seasonal strategies can derive from life-history trade-offs, for instance among vegetative growth, survival, and reproductive effort in the current or future growing seasons (Stearns 1989; Reich 2014). A growth-survival trade-off is for instance well established in perennial grasses with a seasonal endo-dormant phase (Gillespie and Volaire 2017; Volaire 2018). In the grass *Dactilis glomerata*, individual plants that are winter- and summer-dormant have higher survival with winter frost and summer drought, respectively (Bristiel et al. 2018). In the ryegrass *Lolium perenne*, some individuals have high growth rates and are sensitive to drought and frost stress, others are drought-tolerant and have low summer growth rates, and others are frost-tolerant and have low winter growth rates (Keep et al. 2021). Growth-survival trade offs have been further described along altitudinal (Benavides et al. 2015), and latitudinal clines (Koehler et al. 2012) in trees. A trade-off between vegetative growth and reproductive effort has been described for instance in the genus *Fragaria*, in which a deletion in the *GA20OX* gene regulates the fate of axillary meristems between vegetative growth through the production of runners, and flowering (Tenreira et al. 2017). In many perennials, the regulation of flowering varies within species (Friedman 2020), however its adaptive value differs from annuals, in which early flowering followed by senescence can directly translate into an escape strategy.

Here, we study the perennial, Arctic-alpine herb *Arabis alpina*, and in particular the populations from Northern Spain, which are locally adapted on the basis of common garden experiments (Toräng et al. 2015). These populations were identified as outliers in flowering behavior across Europe, since all individual plants (accessions) were unable to flower without vernalization, and flowered for a short time after vernalization (Wunder et al. 2023). This flowering behavior was hypothesized to contribute to adaptation to summer drought (Wunder et al. 2023). *A. alpina* is distributed across the alpine and amphi-Atlantic regions of Europe, the Middle East and Africa, and is thought to have colonized Europe 0.3 to 1.4 million years ago, and Scandinavia in the current interglacial (Koch et al. 2006; Ehrich et al. 2007; Ansell et al. 2011; Karl et al. 2012; Laenen et al. 2018). However, the history of colonization of Spain, and of adaptation to the local conditions are not yet resolved. In this study, we aim to understand the adaptive value of seasonal strategies and of flowering behavior in a perennial plant. To this end, we collected and sequenced the genomes of 211 accessions from Northern Spain, and of 215 from related populations in the French Alps for comparison. We characterized genomic diversity and population structure, we reconstructed the demographic history of colonization of Northern Spain, and we identified genomic signatures of adaptation.

## Results

### Plant material and SNP calling

**Figure 1:**
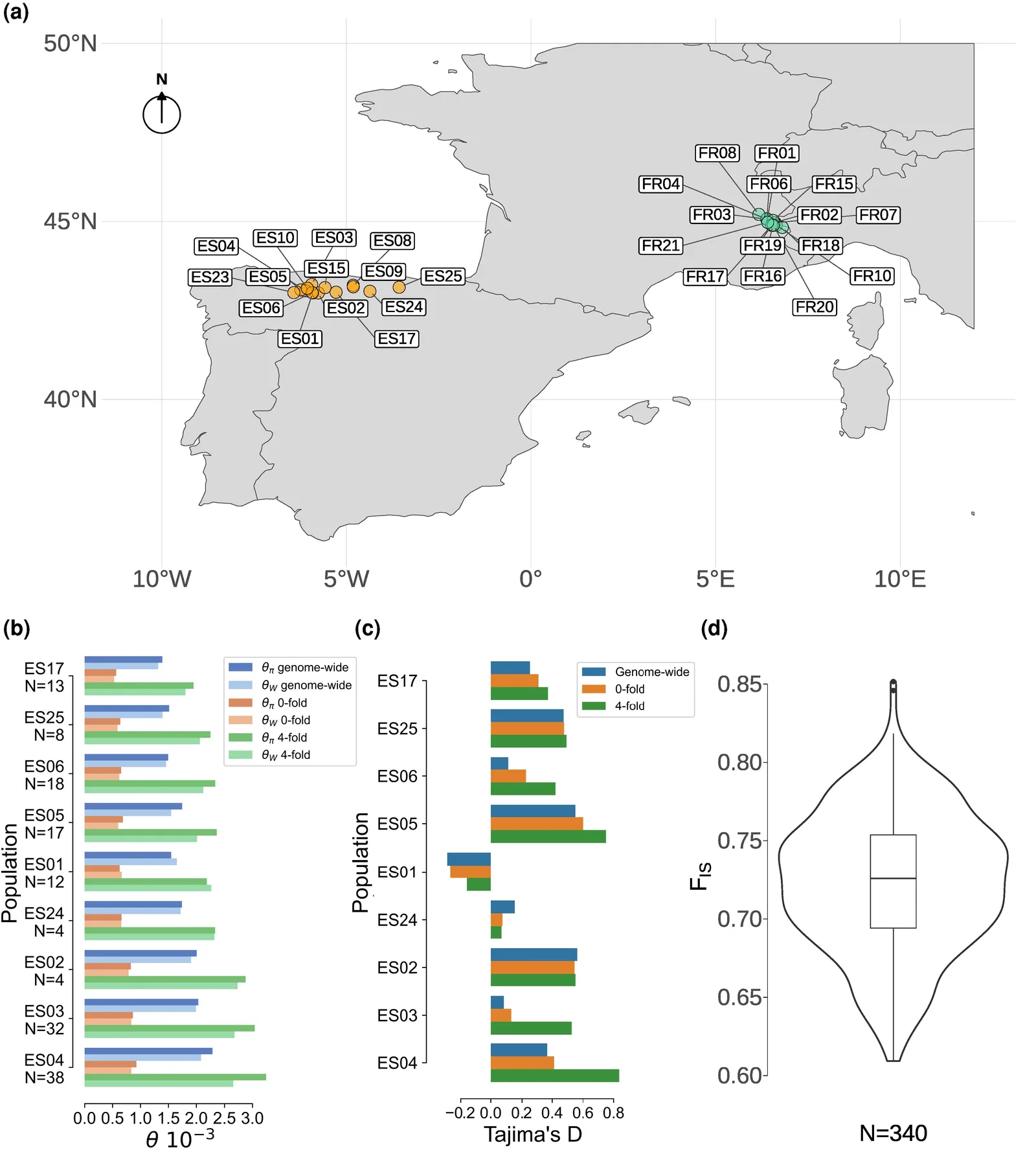
Map of the sampling sites and genomic summary statistics estimated from unrelated samples. a) Map of the sampling sites in Northern Spain (yellow) and in the French Alps (green). Each point represents one population. b) Estimators of the population mutation rate ($\theta_{\pi }$ and $\theta_{W}$) for populations with a sample size N≥4 (*N* is indicated below the population IDs). Estimators are reported genome-wide, and for 0-fold and 4-fold degenerate sites. Populations are sorted by $\theta_{W}$ in ascending order. c) Tajima’s D estimated genome-wide, and for 0-fold and 4-fold degenerate sites, for populations with a sample size N≥4. d) Global inbreeding coefficient ($F_{IS}$), estimated for populations with a sample size N≥10 and calculated in bins of 10 k SNPs.

We sequenced the whole genomes of 211 individual *A. alpina* plants collected in 14 populations in the Cantabrian Mountains (Northern Spain), and of 215 individual plants collected in 15 populations in the French Alps (online supplementary material, Table S1; Fig. 1a). A subset of these accessions were sourced from previous studies (Wunder et al. 2023; Tjeng et al. 2025). The average genome-wide sequencing depth was 19.7× (range across individuals: 14.8×–26.0×). We identified over 7.2 million and 5.8 million high quality SNPs, with less than 30% missing genotypes in the populations from the Cantabrian Mountains and from the Alps, respectively. Using a newly developed approach, ParaMask (Tjeng et al. 2025), we identified a total of 48.6 Mbp and 65.3 Mbp of multicopy genomic regions out of the total 311.6 Mbp of assembled genome for the populations from the Cantabrian Mountains and from the Alps. Additionally, we created a 63.6 Mbp mappability mask using SNPable. After excluding these regions for a total of 125.9 Mbp, we retained over 4.6 million SNPs for the samples from the Cantabrian Mountains and over 3.2 million SNPs for the samples from the Alps. After further filtering steps that are specific for some of the analyses (described in Materials and Methods), we used 916,101 SNPs for the neighbor-joining tree and the principal coordinate analysis (PCoA), 629,967 SNPs for ADMIXTURE, and 2,967,704 SNPs for demographic inference. Depending on the test set used in selection scans with *3P-CLR*, we used between 2.3 and 2.5 million SNPs.

### Populations in the Cantabrian Mountains (Northern Spain) are genetically differentiated from each other

In order to understand the general pattern of genetic variation in the populations from the Cantabrian Mountains, wecharacterized genetic diversity and population structure. Genetic diversity measured at 4-fold degenerate sites with Watterson’s estimator of theta ($\theta_{W}$) ranged from $1.81\times 10^{-3}$ in population ES17 to $2.73\times 10^{-3}$ in population ES02, and the average pairwise differences ($\theta_{\pi }$) ranged from $1.95\times 10^{-3}$ in population ES17 to $3.25\times 10^{-3}$ in population ES04 (Fig. 1b). Within each population, diversity was higher at 4-fold degenerate sites, and lower at 0-fold degenerate sites than genome-wide, consistent with higher conservation at functional sites. Tajima’s D was positive in almost all populations, and between 0.069 in ES24 and 0.837 in ES04 at 4-fold degenerate sites, which indicates a slight excess of intermediate-frequency variants (Fig. 1c). Population ES01 was the only exception with a negative Tajima’s D of −0.154, suggesting a recent population expansion. Tajima’s D at 4-fold degenerate sites was slightly more positive than at 0-fold degenerate sites in several, but not in all populations. The mean inbreeding coefficient ($F_{IS}$) across bins of 10,000 SNPs was 0.722, with a standard deviation of 0.047 (Fig. 1d). Considering that these populations are self-compatible (Toräng et al. 2017; Laenen et al. 2018), this corresponds to an outcrossing rate of 16.1% if inbreeding is only due to selfing, or higher than that if other causes of inbreeding also play a role, for instance population structure.

The average density of genetic differences per pair of samples was 1.59-fold higher across populations than within populations (on average $3.11\times 10^{-3}$ across populations and $1.96\times 10^{-3}$ within populations; online supplementary material, Fig. S1a). The distribution within populations was unimodal, and had a tail of low-density pairwise differences due to closer relatedness among individuals. The distribution among populations, on the other hand, was multimodal with three main peaks of pairwise differences. The mode at $2.81\times 10^{-3}$ differences included pairs of individuals from Western locations. The mode at $3.50\times 10^{-3}$ differences included pairs with one individual from a Western population, and one from a Central-Eastern location, excluding the easternmost population, ES25. The mode at $4.00\times 10^{-3}$ differences included all sample pairs with one individual from ES25. Consistent with a high selfing rate, most individuals had low heterozygosity ($\mathrm{median}=1.68\times 10^{-4}$), and few had heterozygosity in the range of the pairwise differences within populations (up to $2.2\times 10^{-3}$), likely due to rare outcrossing events (online supplementary material, Fig. S1b). Genetic distances were correlated with geographic distances (Mantel correlation=0.786, $p\text{-value}\;<1\times 10^{-4}$), even after controlling for environmental distances (Partial Mantel correlation=0.754, $p\text{-value}\;<1\times 10^{-4}$). The correlation between genetic and environmental distances was also significant but weaker (Mantel correlation=0.485, $p\text{-value}\;<1\times 10^{-4}$), and its strength decreased further after controlling for geographic distances (Partial Mantel correlation=0.366, $p\text{-value}\;<1\times 10^{-4}$; online supplementary material, Fig. S1c; Table S2).

**Figure 2:**
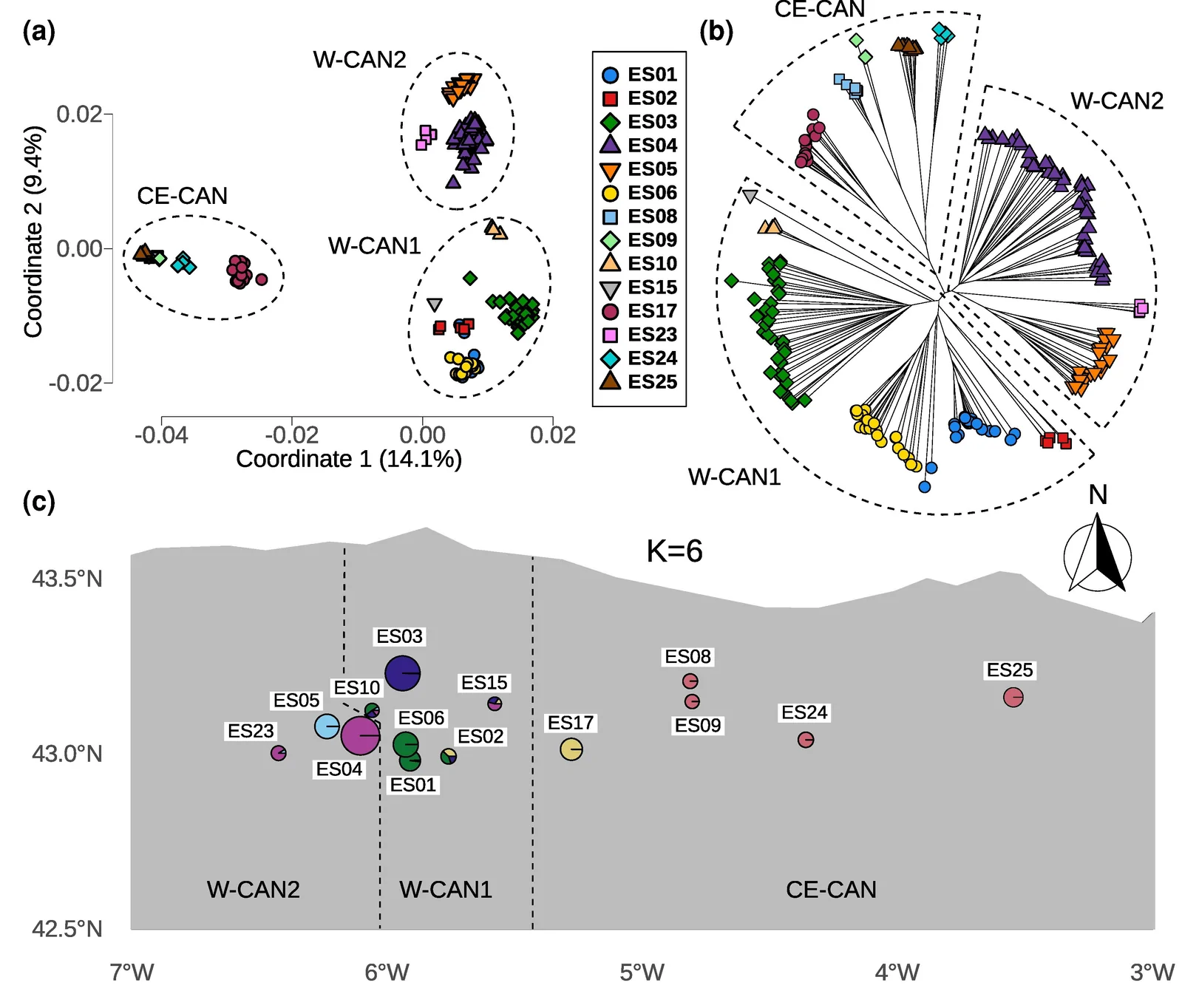
Analysis of genetic clustering among *A. alpina* populations from the Cantabrian Mountains. The three major structure groups Central-Eastern Cantabrian Mountains (CE-CAN), Western Cantabrian Mountains 1 (W-CAN1), and Western Cantabrian Mountains 2 (W-CAN2) are highlighted by dashed lines. a) Principal coordinate analysis (PCoA) based on genetic distances (1-IBS, identity by state). Samples are arranged in the first two coordinates, which capture the greatest variation of the original distances. Labels indicate the percentage of variation explained by each axis. Shapes and colors denote different populations. b) Unrooted neighbor-joining (NJ) tree. Shapes and colors denote different populations, as in panel (a). c) Geographic distribution of shared genetic ancestry inferred with ADMIXTURE. The number of ancestry groups is set to K=6, based on cross-validation error. The pie charts represent the proportions of genetic ancestry, and circle diameters are proportional to sample size.

Clustering of the genomic data with a principal coordinate analysis (PCoA) based on a distance matrix identified three main genetic clusters (Fig. 2a; online supplementary material, Fig. S2). The first coordinate of the PCoA separated Central-Eastern populations (CE-CAN), including samples from locality Puebla de Lillo (ES17), Picos de Europa (ES08 and ES09), and Eastern locations (ES24 and ES25). The second coordinate differentiated two groups within the Western region (W-CAN1 and W-CAN2), one including localities between Angliru and Valle de Riotuerto (ES01, ES02, ES03, ES06, ES10, ES15), and the other including localities between Leitariegos and La Cueva (ES04, ES05, ES23). Similarly, a neighbor-joining tree separated the Central-Eastern populations with a long internal branch, and the two groups of Western populations with shorter internal branches (Fig. 2b). In both analyses, genetic clustering aligns with the geographic location of sample collection, suggesting clear differentiation among populations. We analyzed hierarchical population structure with ADMIXTURE, after sub-sampling the data set to 173 unrelated individuals (Materials and Methods). This analysis identified six ancestry groups based on cross-validation (CV) errors across 10 replicate runs (mean CV: 0.2534), and similarly low CV for up to nine groups (*t*-test: p-value=0.2916; online supplementary material, Fig. S3). Across runs, populations from CE-CAN were assigned to the same ancestry group, despite a relatively large geographical distance between populations (at most around 65 km; Fig. 2c). Genetic ancestry was private to most other populations, with the exception of populations ES23 - ES04, and ES01 - ES06, respectively within W-CAN1 and W-CAN2, which shared genetic ancestry (Fig. 2c). Overall, the analysis of genetic diversity and population structure revealed clear differentiation among populations in the Cantabrian Mountains, and in particular among Central-Eastern locations and two genetic clusters in the Western area.

### Demographic inference suggests that the Cantabrian Mountains were colonized during the last glacial period in a range expansion from east to west

**Figure 3:**
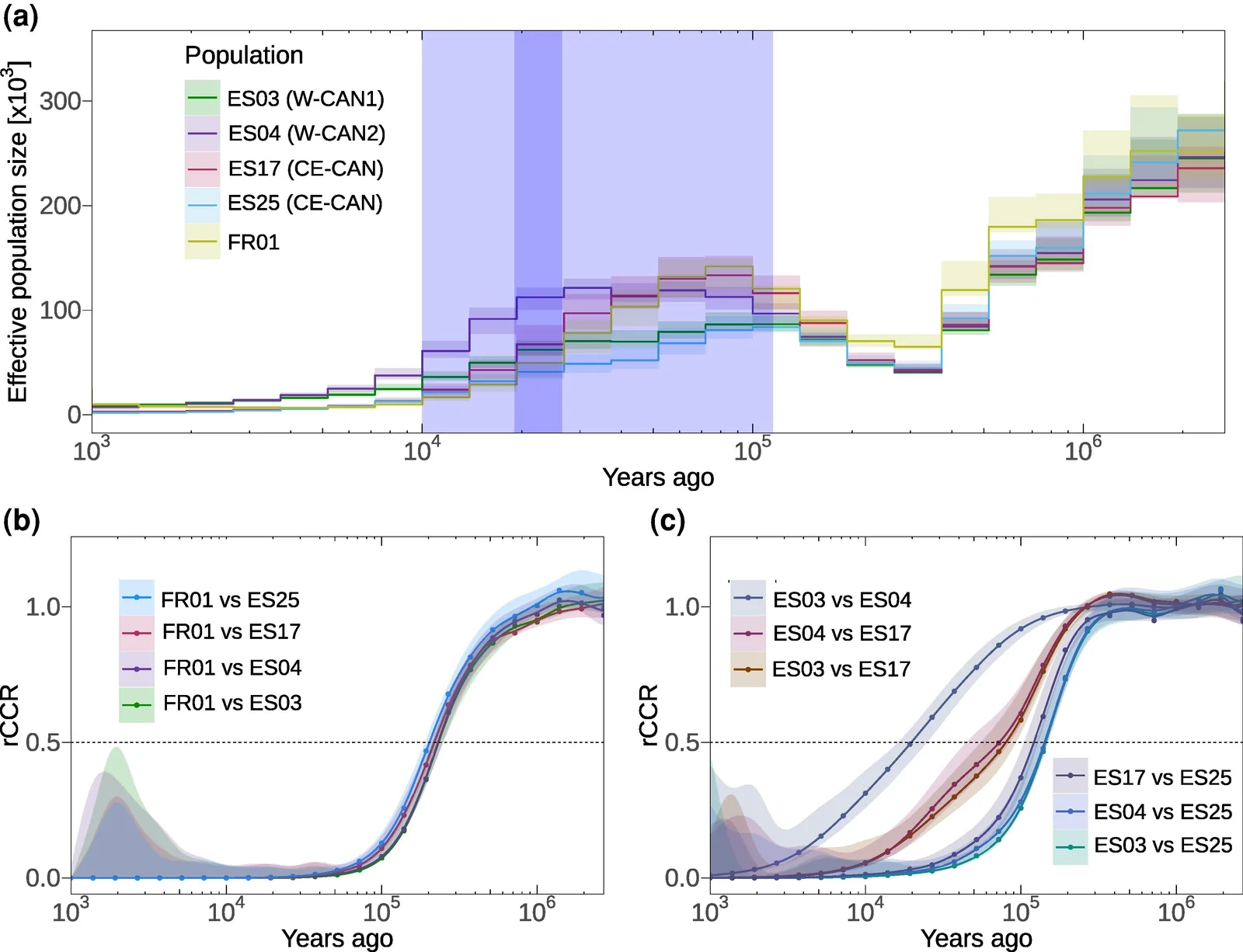
Estimates of effective population size as a function of time, and of split times among populations based on the relative cross coalescent rates (rCCR). Lines represent point estimates, and shaded colors represent the 95% confidence intervals based on 100 bootstrap replicates. Four representative populations are shown (ES03, ES04, ES17, and ES25), and the French population FR01 is used as an outgroup. Split times for all population pairs can be found in online supplementary material, Table S3. a) Effective population size across time. The plot area shaded in light blue represents the last glacial episode, and the area shaded in dark blue represents the last glacial maximum. b) rCCR between populations from the Alps and from the Cantabrian Mountains. A cutoff of rCCR=0.5, indicated by the horizontal line, is used to infer split times. c) rCCR among populations from the Cantabrian Mountains. A cutoff of rCCR=0.5, indicated by the horizontal line, is used to infer split times.

We reconstructed the demographic history of populations from the Cantabrian Mountains and from the French Alps using the hidden Markov model (HMM) based method Relate (Speidel et al. 2019). Consistent across populations, this inference revealed a period of low effective population size between around 400 and 200 thousand years ago (kya) (Fig. 3a). This was followed by a period of large effective population size that started between 100 and 200 kya, and lasted until between 10 and 20 kya. This period approximately coincides with the last glacial period, between 115 and 11.7 kya, but it may also have been influenced by the penultimate glacial period, between 194 and 135 kya (Dahl-Jensen et al. 2013). Subsequently, effective population sizes declined until present, in a period that roughly coincides with the current interglacial, between the last glacial maximum (LGM), around 21 kya, and present (Clark et al. 2009).

To infer the time in the past when populations split from each other, we used the relative cross coalescent rate (rCCR) statistic, and a threshold of 0.5 (Schiffels and Durbin 2014). Split times between populations in the Cantabrian Mountains and FR01, which we used as an outgroup from the French Alps, ranged between 198 and 230 kya (Fig. 3b). This period approximately coincides with the start of the penultimate glacial period, around 194 kya. Split times among populations from the Cantabrian Mountains ranged between 6 and 150 kya (Fig. 3c; online supplementary material, Table S3), in a period that mostly coincides, or just predates the last glacial period. In comparisons between populations from CE-CAN and W-CAN1 or W-CAN2, split times were 1.1 to 8.4 (mean: 3.8) times older than the splits among W-CAN groups. This pattern might result from more recent split times, or more recent gene flow among Western compared to Eastern groups. Split times within ancestry groups ranged between 6 and 34 kya in W-CAN1, between 10 and 20 kya in W-CAN2, and between 23 and 140 kya in CE-CAN. These recent split times within ancestry groups suggest that fragmentation among populations mostly happened in the current interglacial, since the LGM. Split times were positively correlated with the longitude of the easternmost population across population pairs (Pearson correlation coefficient based on $10^{6}$ permutations: r=0.836, $p\text{-value}=2.70\cdot 10^{-5}$; online supplementary material, Fig. S4), and not with the longitude of the westernmost population across pairs (r=0.140, p-value=0.47), which suggests that the Cantabrian Mountains were colonized from east to west (details in online Supplementary material). We note that the relative order of events inferred in these analyses is more robust to assumptions (e.g. on the mutation rate and generation time, described in online Supplementary material) than the absolute time estimates.

### Detection of genomic regions with signatures of positive selection

Our reconstruction of the history of *A. alpina* in the Cantabrian Mountains revealed a decrease in genomic diversity concomitant with a change in climatic conditions in the current interglacial (Fig. 3a). Furthermore, the sites in Northern Spain are more arid in summer compared to other collection sites across Europe, as a result of warmer temperatures and lower precipitation (online supplementary material, Fig. S5; Table S4, S5, and S6). Summer aridity in Spanish sites was also supported by data loggers recording soil temperature and humidity that we installed in close proximity to natural plants in a subset of the collection sites (online supplementary material, Fig. S6). To understand whether and how *A. alpina* adapted to the local conditions, we screened the genomes for signatures of positive selection using *3P-CLR* (Racimo 2016). This method can account for structured populations, and detect selective sweeps that happened in the two terminal branches that lead to each of two related focus populations, and sweeps that happened in the branch that is ancestral to both, compared to an outgroup. For each genetic cluster, we first estimated drift rates, which are rates of allele frequency change due to genetic drift that depend on divergence times and on effective population size, $N_{e}$, and are equivalent to scaled F3 statistics (Racimo 2016). Drift rates were generally consistent with the results from demographic inference, however they were negative between W-CAN and CE-CAN excluding population ES17 (more detail in online Supplementary material). Negative drift rates suggested admixture between CE-CAN (excluding ES17) and populations in the French Alps, which would violate assumptions of *3P-CLR* if CE-CAN was used as a focus population, but can be tolerated if used as outgroup. As a consequence of this, we used as focus clusters W-CAN1, W-CAN2, and ES17, and we repeated the analysis with populations from the French Alps, or from CE-CAN excluding ES17 as outgroups (details in online Supplementary material; the sets of populations used are shown in online supplementary material, Table S7). In these analyses, we used the upper 0.5% quantile of empirical CLR scores as a threshold to focus on the genomic regions with the strongest evidence for selection. Although *3P-CLR* accounts for the main effect of demography by using estimated drift rates, we simulated the demographic history of the studied populations for comparison (online supplementary material, Fig. S7). Across replicates, sets, and branches, the empirical threshold was generally conservative and in line with the 0.5% to 1% quantiles in the simulations, and it was never less conservative than the 5% quantile (online supplementary material, Table S8; details in online Supplementary material). Furthermore, the empirical data was enriched in high *3P-CLR* scores compared with neutral simulations in most tested branches, consistent with the importance of selection in the history of the studied populations (online supplementary material, Fig. S8).

**Figure 4:**
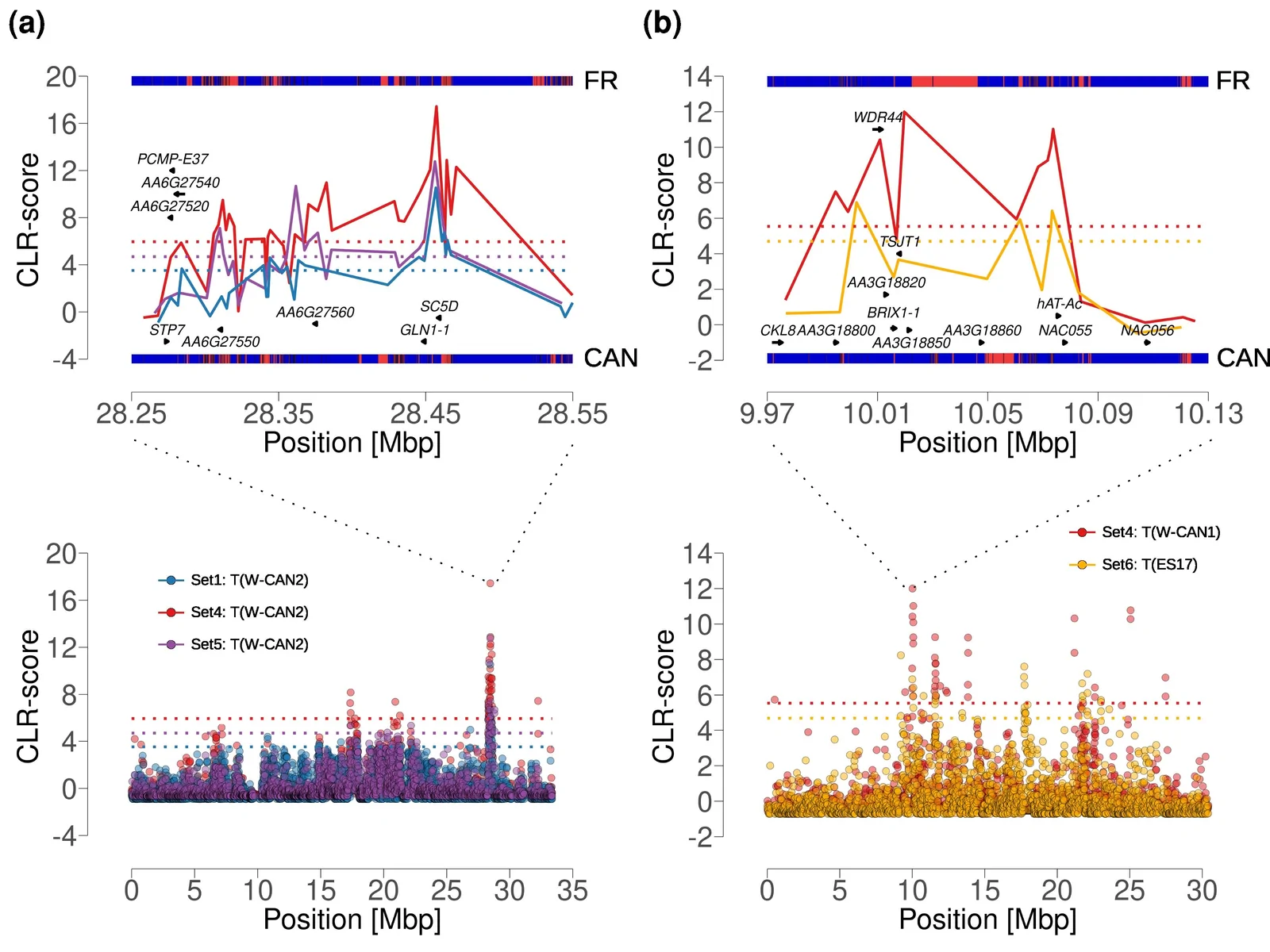
Standardized composite likelihood ratio scores at the *SC5D* (**a**) and *NAC055* (**b**) loci, shown zoomed in (top) and across the chromosome (bottom). Genes are indicated by arrows and labeled with their *A. thaliana* ortholog or, if no ortholog is known, by their *A. alpina* gene identifier. Dotted lines show the 0.5% most extreme CLR scores for each analysis. Segments in the top panels show multicopy (red) and single-copy (blue) regions in France (top segment) and in the Cantabrian Mountains (bottom segment) inferred by ParaMask. The sets in the legend represent different combinations of populations (explained in online supplementary material, Table S3). a) Scores for the terminal branch of W-CAN2 and for different group sets in the *3P-CLR* analysis (blue – Set1; red – Set4; purple – Set5). b) Scores for the terminal branch of W-CAN1 (red – Set4) and for ES17 (yellow – Set6). The DNA transposon (hAT-Ac) upstream of the gene is also indicated.

Across all combinations of focus populations and outgroups, the top CLR peak was on the terminal branch leading to W-CAN2, and overlapped the gene *SC5D* (Fig. 4a). *SC5D* also overlapped the top peak based on cross-population extended haplotype homozygosity (*XP-EHH*; online supplementary material, Fig. S9). *SC5D* encodes a C-5 sterol desaturase, and a premature stop codon in *A. thaliana* impairs brassinosteroid biosynthesis, and underlies the dwarf mutant *dwf7* (Choe et al. 1999). The top five CLR peaks on the terminal branches that led to W-CAN1, W-CAN2, and ES17 overlapped genes with functions in lipid biosynthesis and membrane fluidity (in the endoplasmic reticulum and chloroplast), cell-wall modification and scaffolding, and the synthesis and turnover of surface-secreted metabolites (online supplementary material, Table S9). Genes within the upper 0.5% tail of CLR scores were enriched in GO categories associated with energy generation, solute transport, core metabolism, and abiotic stress responses (p-value≤0.5%; online supplementary material, Table S10). On the branch ancestral to W-CAN1, W-CAN2 and ES17, the top five CLR peaks overlapped genes involved in growth control, protein turnover, membrane assembly and metabolism, and defense mechanisms (online supplementary material, Table S9). The top 0.5% CLR peaks on the ancestral branch were enriched for genes related to the negative regulation of growth and positive regulation of transcription (online supplementary material, Table S10). However we note that this specific ancestral branch did not have an enrichment of high CLR scores compared to simulations (online supplementary material, Fig. S8). Because several enriched GO categories were related to general metabolic functions, we screened the LUCAS survey 2018 (Fernandez et al. 2022) for differences in edaphic conditions between sites in Cantabrian Mountains and in the Alps. However, the soil data collected within 10 km of our collection sites were limited to very low sample sizes, and did not allow us to draw conclusions about these differences (online Supplementary material). The analyses with CE-CAN as outgroup are discussed in online Supplementary material.

To find candidate genes potentially involved in adaptation to drought, we screened the genes in the upper 0.5% quantile of CLR scores for drought-related GO terms (response to water deprivation and heat; GO:0009414, GO:0009408). We found 16 candidate genes associated with drought-related GO terms, of which 11 were found on terminal branches, and five on the branch ancestral to W-CAN1, W-CAN2, and ES17 (online supplementary material, Table S11). Among these, we found transcription factors that are partially responsive to ABA (e.g.: *NAC055*, *ZHD11*, *MYB94*), stress induced enzymes (*ASPG1*, *PAL2*), proteins that stabilize RNA (*RH25*, *RH26*), and chaperones (*HSP15.7*, *CPN10*, *FKBP62*). At the NAC transcription factor *NAC055* (Fig. 4b) we identified an inframe 3bp insertion at residue 251, which translates into the insertion of a phenylalanine (details in online Supplementary material). Furthermore, we found a transposable element (TE) insertion 892 bp upstream of the transcription start site in all and only the individuals that lacked the 3bp insertion. The 3bp insertion was fixed in populations ES17 and ES05, which have the two most arid climates in July ($AI_{ES17}=0.250$; $AI_{ES05}=0.286$; online supplementary material, Table S6), and the aridity index in July was a highly significant predictor of allele dosage at the 3bp insertion (binomial regression with four principal coordinates from genome-wide structure as covariates; $\text{Wald}\;p\text{-value}=6.26\times 10^{-5}$; online supplementary material, Fig. S10; more detail in online Supplementary material). The 3bp insertion and the TE are candidate genetic variants that might change the function of the gene and underlie adaptation to drought.

*A. alpina* accessions from the Cantabrian Mountains were previously identified as outliers for flowering behavior across Europe, in particular in the response to vernalization (Wunder et al. 2023), which we confirmed here in a controlled greenhouse experiment (online supplementary material, Fig. S11). In order to identify adaptive genetic variants potentially involved in the response to vernalization, we inspected the upper 0.5% CLR tails of all sets and branches. We identified three candidate genes potentially associated with vernalization responses, *VERNALIZATION INDEPENDENCE 4* (*VIP4*) in the terminal branch leading to W-CAN2, *VIN3-LIKE PROTEIN 1* (*VIL1*) in the terminal branch leading to ES17, and *FRIGIDA INTERACTING PROTEIN 1* (*FIP1*) in the terminal branch leading to W-CAN2 (online supplementary material, Table S12; online supplementary material, Fig. S12). *VIP4* is a positive regulator of *FLC* expression, and mutants flower even earlier than *FLC* null alleles (Zhang and Van Nocker 2002). At *VIP4*, we identified two non-conservative missense mutations (E212V) and (D184H) located in a disordered region of the protein. Notably, these substitutions change an acidic glutamic acid (E) and an aspartic acid (D) residues, which are conserved in *Arabidopsis* (UniProtKB:Q9FNQ0), *Brassica napus* (EnsemblPlants GeneID:Bra035940), and in *Arabis montbretiana* to a hydrophobic and a positively charged residues, respectively. The two mutations were nearly fixed in W-CAN2 (at 96.6% and 97.8% frequency, respectively), they were segregating in W-CAN1 (at 64.2%, and 66.9% frequency, respectively), and were absent in both CE-CAN and French populations. *VIL1* interacts with *VIN3* to epigenetically silence *FLC* expression (Sung et al. 2006). At *VIL1*, we identified a deletion of residues Asp472–Asn473 within a disordered region upstream of the VIN3-interacting domain. This variant segregated at higher frequency in W-CAN2 (54.9%) than in W-CAN1 (7.4%), and in France (5.9%), and was absent in CE-CAN. *FIP1* encodes a known interactor of *FRI*, however its mutant phenotype in *A. thaliana* flowers at the same time as wild type (Sung et al. 2006).

### Identification of genes with a different selection regime in the Cantabrian Mountains compared to the Alps

To detect differences in the mode and strength of selection between populations from the Cantabrian Mountains and from the Alps, we compared the rate ratios of non-synonymous to synonymous substitutions ($d_{N}/d_{S}$) and polymorphisms ($p_{N}/p_{S}$) across all 28,745 annotated genes, and using *A. montbretiana* as the outgroup. This test can, in principle, identify genes that differ between the two regions in signatures of local adaptation, as well as of purifying, linked, or other modes of selection. For this, we used a logistic regression for correlated data (i.e. generalized estimating equations, GEE). This test accounts for mutations that originated in the ancestral population, which can contribute to polymorphism and substitutions in both the Cantabrian Mountains and in the Alps. By comparing rate ratios in the two regions to the genome-wide average with a Wald test, we accounted for the baseline level of conservation of each gene (i.e. the strength of purifying selection), and for the main effect of demography (see online Supplementary material for details).

Three genes significantly differed in substitution rates after FDR correction for multiple hypothesis testing, *TPS9*, *OXS2*, and *PSD* (Table 1). The three genes had lower $d_{N}/d_{S}$ in populations from the Cantabrian Mountains than in populations from the Alps relative to the genome-wide average, and were generally associated with abiotic stress responses, and with the regulation of developmental processes and of flowering.

**Table 1:** Genes with significantly different rate ratios of non-synonymous to synonymous substitutions or polymorphism in populations from the Cantabrian Mountains and from the Alps.

	Ortholog		Cantabrian Mountains			Alps					
*A. alpina* ID	Gene name	ID	$D_{N}$	$D_{S}$	$d_{N}/d_{S}$	$D_{N}$	$D_{S}$	$d_{N}/d_{S}$	logit coeff	*p*-value	FDR *p*-value
AA1G30250	*TPS9*	AT1G23870	5	61	0.022	5	43	0.032	−0.349674	0.000010	0.022613
AA4G34590	*C3H30*	AT2G41900	3	35	0.025	3	16	0.055	−0.782759	0.000037	0.042277
AA2G20390	*PSD*	AT1G72560	5	50	0.028	5	35	0.041	−0.356675	0.000055	0.042572

			$P_{N}$	$P_{S}$	$p_{N}/p_{S}$	$P_{N}$	$P_{S}$	$p_{N}/p_{S}$			
AA1G15460	*At4g11900*	AT4G11900	109	53	0.555	106	53	0.540	0.027909	$8.34\times 10^{-07}$	0.004747
AA5G10450	*MEE39*	AT3G46330	25	37	0.191	26	30	0.245	−0.248941	$3.47\times 10^{-06}$	0.009084
AA4G03560	*MHC9.2*	AT3G21340	39	26	0.420	46	16	0.805	−0.650588	$4.79\times 10^{-06}$	0.009084

*A. alpina ID*, *gene name*, and *ID* show gene identifiers in *A. alpina* and *A. thaliana*. Shown are the number of derived non-synonymous ($D_{N}$) and synonymous substitutions ($D_{S}$), and their rate ratios ($d_{N}$/$d_{S}$), and the number of derived non-synonymous ($P_{N}$) and synonymous polymorphisms ($P_{S}$) and their rate ratios ($p_{N}$/$p_{S}$), in populations from the Cantabrian Mountains and from the Alps, sorted by p-value. The *logit coef* represents the logit coefficient from the GEE, *p-value* and *FDR p-value* are from the Wald test before and after correction.

In *A. thaliana*, *TPS9* is likely involved in trehalose biosynthesis and regulates responses to salt and cold stress as well as flowering time in long days (Tian et al. 2021; Vishal et al. 2024; Zhong et al. 2024). *OXS2* encodes a zinc-finger transcription factor involved in oxidative and salt stress responses (Blanvillain et al. 2011; Jing et al. 2019). *PSD* encodes a karyopherin-family nuclear transport receptor required for proper shoot apical meristem development and organ differentiation (Hunter et al. 2003; Li and Chen 2003).

In the analysis of polymorphism rates, we detected three genes that significantly differed after FDR correction, the ortholog of *At4g11900* in *A. thaliana*, *MEE39*, and *MHC9.2* (Table 1). In the populations from Cantabrian Mountains, $p_{N}/p_{S}$ was higher at *At4g11900* and lower at *MEE39* and *MHC9.2* relative to the genome-wide average difference with populations from the Alps. All three genes encode receptor-like serine/threonine-protein kinases, which are primarily involved in stress- and defense- related signaling pathways.

The functions of *At4g11900* and *MHC9.2* have not been characterized. *MEE39* is a Leucine-rich repeat receptor-like serine/threonine-protein kinase that mediates maternal effects, and that is required for the correct development of the endosperm and of the embryo (Pagnussat et al. 2005).

We further tested single genes for differences in the ratios of the average density of pairwise differences (a measure of genetic diversity) at non-synonymous and at synonymous sites ($\theta_{\pi_{n}}/\theta_{\pi_{s}}$) between populations from Cantabrian Mountains and from the Alps. To account for the genome-wide effects of demography, we compared single genes with the genome-wide average difference, using a Z-test (details in online Supplementary material). The genes with significant differences after FDR correction (p-value<0.005) were 1033. These genes were enriched for GO categories generally related to the regulation of photosynthesis, potentially in response to stress, for instance including organellar RNA metabolism, photosynthetic machinery assembly, and isoprenoid and sterol metabolism (online supplementary material, Table S13). These pathways provide precursors for chlorophyll phytol side chains, carotenoids, sterols, and several isoprenoid-derived hormones (via the plastidial MEP pathway, and sterol metabolism Vranová et al. 2013). Response to Cadmium was the only enriched category with a specific biological function. The significant genes involved in this category were related to the mitigation of stress caused by metals, potentially acting on photosynthesis-related functions, rather than to metal transport.

Because none of these candidate genes were involved in the vernalization response, which is divergent in accessions from the Cantabrian Mountains (Wunder et al. 2023), we screened the genes that were significant before FDR correction. We note that these genes lack a definitive signature of selection, and we mention them only as potential candidates. Among these genes, *VIP5* and *UBP26* differed in substitution rates, *CDC5* and *FRL1* differed in polymorphism rates, and *AA4G13300*, a paralog of *FRL1*, differed in diversity ratios (online supplementary material, Table S14). *VIP5*, *UBP26*, and *CDC5* can have indirect effects on the vernalization response through their general involvement in epigenetic modifications (*VIP5*, *UBP26*) and in splicing (*CDC5*). *FRL1*, on the other hand, was the only candidate directly involved in the core vernalization pathway.

**Figure 5:**
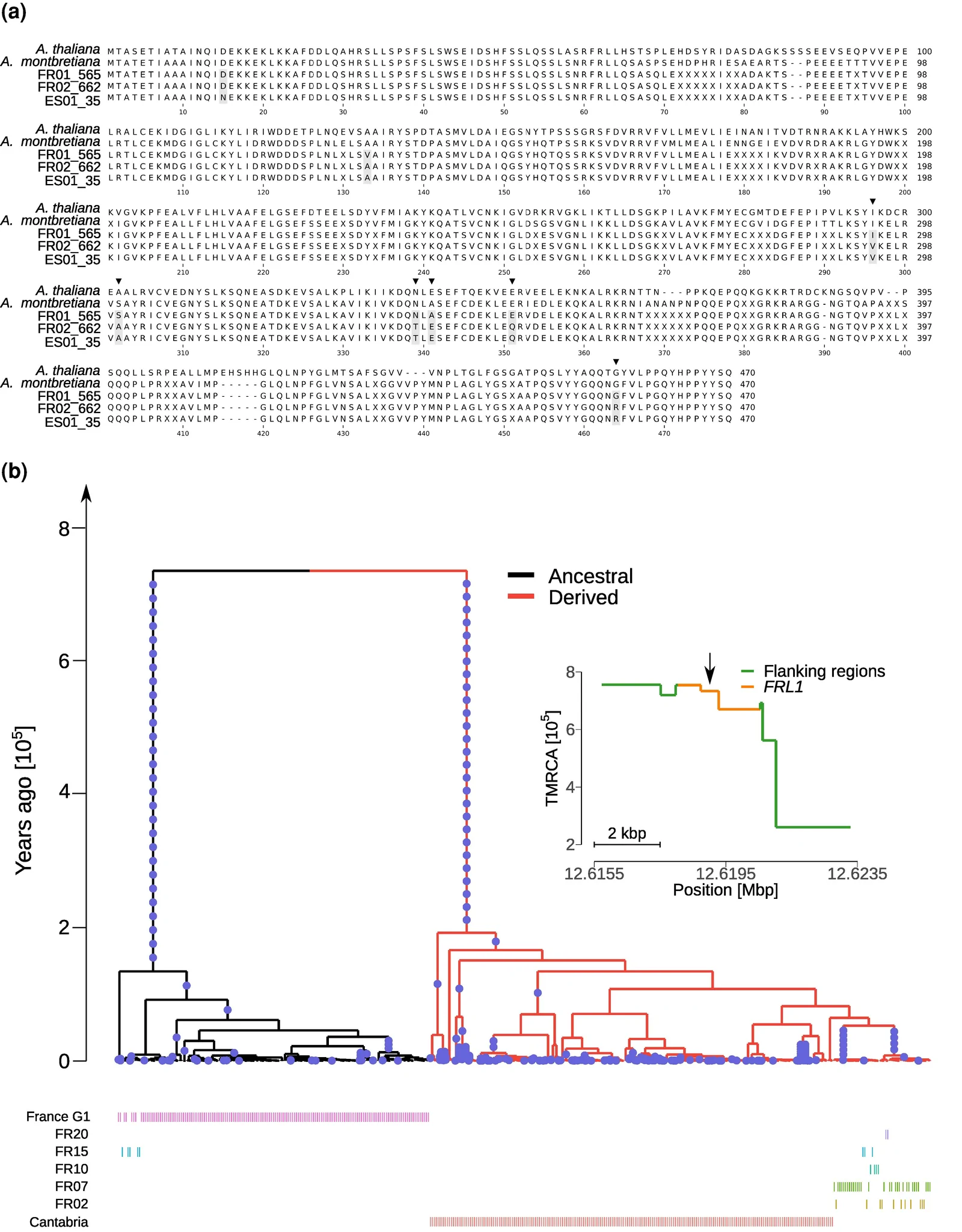
Genetic variation at the *FRL1* locus. a) Alignment of the FRL1 protein sequence from *A. thaliana*, *A. montbretiana*, and *A. alpina* from the Alps and from the Cantabrian Mountains. The six amino acid differences marked with black triangles are fixed derived in populations from the Cantabrian Mountains, and the two amino acid differences highlighted in green are segregating. b) The genealogy at the *FRL1* locus that spans five of the six non-synonymous mutations. Red branches indicate the lineages that carry the derived alleles at these five mutations, and black branches represent lineages that carry the ancestral alleles. France G1 (group 1) includes populations FR01, FR03, FR04, FR06, FR08, FR16, FR17, FR18, FR19, and FR21. The inset shows the time to the most recent common ancestor (tmrca) distribution across genealogies at the *FRL1* locus. The tmrca of the genealogy depicted here is marked by an arrow.

In *A. thaliana*, FRL1 interacts with FRI as part of the FRI-C protein complex which contributes to the activation of *FLC* expression (Choi et al. 2011), until the plant experiences a cold treatment (vernalization), and FRI-C forms nuclear condensates (Zhu et al. 2021). *FRL1* had 21 and 15 non-synonymous substitutions respectively in the populations from the Cantabrian Mountains and from the Alps, compared to the outgroup *A. montbretiana*, and was therefore highly diverged. Near the C-terminus of the gene, we found a cluster of six non-synonymous mutations that were fixed derived in the Cantabrian Mountains compared to *A. montbretiana* (Fig. 5a). These mutations were linked into a haplotype that also segregated in the Alps at 23.7% frequency (online supplementary material, Table S15). In order to better understand the history of this polymorphism, we extracted from Relate the genealogies at and around *FRL1*. A single genealogy overlapped five of the six non-synonymous mutations (Fig. 5b; online supplementary material, Table S16), and a second genealogy overlapped the remaining one (online supplementary material, Fig. S13). In the genealogy that overlapped most of this cluster of mutations, divergence between haplotypes dated back to the TMRCA and was therefore rather ancient, although not more than the genome-wide TMRCA distribution (TMRCA=734kya, empirical p-value=0.443). This genealogy was unusually balanced in comparison with genome-wide genealogies (Colless Index=2,933, empiricalp-value=0.037), which implies that dichotomous splits separated lineages into subsets of similar size. The span of this genealogy (the number of bp between recombination events) was not particularly unusual (span=544 bp, empiricalp-value=0.169), however similarly balanced root splits extended across eleven consecutive neighboring genealogies with 97.7% agreement across 11.6 kbp. To evaluate whether the length of the full balanced haplotype (the 11 genealogies concatenated) was unusual, we screened genome-wide for similarly balanced root-split segments (details in online Supplementary material). Among 63,189 similarly balanced segments, only 17 were longer than the focal haplotype segment ($\text{empirical }p\text{-value}=2.74\times 10^{-4}$), which might be due to a common history across a functional unit that extends across the *FRL1* locus.

Finally, we assessed whether this polymorphism was also found in published data of seven other *Arabis* species, in *A. thaliana*, and in diverged outcrossing populations of *A. alpina* (online supplementary material, Table S15). The seven *Arabis* species and *A. thaliana* shared the *FRL1* allele that is common in the Alps at four out of the six positions. The remaining two positions varied among these species, and matched in some cases the allele found in the Alps, and in others the allele found in Cantabrian Mountains. This can be either due to shared ancestral variation, or to repeated mutations. In outcrossing *A. alpina*, both haplotypes segregated as in the populations studied here.

Consistent with an effect of the *FRL1* polymorphism on flowering regulation, accessions with the haplotype fixed in the populations from Cantabrian Mountains and segregating in the Alps had a 72% lower hazard of flowering compared with accessions with the alternative haplotype (Cox proportional-hazards model with region of origin as covariate, z=−7.41, $p\text{-value}=1.27\times 10^{-13}$). The difference was even greater (96% difference in hazard) without region of origin as covariate (z=−18.73, $p\text{-value}=2.0\times 10^{-16}$; online supplementary material, Fig. S14).

## Discussion

Plants with an Arctic-alpine distribution are challenged by abiotic factors, such as extreme temperatures, low water availability, and short favorable seasons, and can reveal the dynamics of adaptation to abiotic stress. Moreover, these species experience habitat loss and fragmentation due to climate change, and studying their evolution and genomic diversity can reveal possible responses to a warming climate. In this study, we focused on the perennial, Arctic-alpine herb *A. alpina* and in particular on populations from the Cantabrian Mountains in Northern Spain, where the growing season is longer, warmer, and drier than in other regions of the range. Furthermore, these populations have been previously identified as outliers for flowering phenology, because they have a strong requirement for vernalization, and they flower for a short duration after vernalization (Wunder et al. 2023). In order to understand the evolutionary history of these populations and the adaptive value of flowering behavior, we characterized genomic diversity, we reconstructed their demographic history, and we analyzed genomic signatures of adaptation in comparison to outgroup populations from the Alps.

### *A. alpina* populations from the Cantabrian Mountains are a diverged genetic lineage, and demographic inference suggests a colonization during the last glacial period

The analysis of genomic diversity revealed strong differentiation between populations from the Cantabrian Mountains and from the French Alps, which was as ancient as 216 kya (198–230 kya) in our inference. This divergence coincides with the start of the penultimate glacial period between 194 and 135 kya, and it predates the most ancient splits species wide in the model plant *A. thaliana* (around 90 kya), and its colonization of Eurasia (around 20–40 kya) (Durvasula et al. 2017). Therefore, in *A. alpina*, the Iberian Peninsula harbors a highly diverged genetic group, similar to the so-called Iberian relicts in *A. thaliana* (The 1001 Genomes Consortium 2016). However, the relicts in *A. thaliana* co-occur with other ancestry groups of different origin in Iberia (Castilla et al. 2020), while in *A. alpina* we identified this single genetic group in the Cantabrian Mountains. Our demographic reconstruction suggests a strong influence of the last glacial episode, between approximately 115 and 12 kya, on historic effective population sizes in *A. alpina*. During this period, the Alps were covered by an ice shield (Ivy-Ochs et al. 2009) and the Iberian peninsula was a refugium for many species like bear, hedgehog, grasshoppers, and oak (Hewitt 2000; Petit et al. 2002). In populations from the Cantabrian Mountains and from the Alps, this glacial episode coincides with the signature of a widespread increase in effective population sizes. This suggests that the suitable habitat for *A. alpina* was larger at that time, and likely located at lower altitude, which may have aided the colonization of Northern Spain. Different from our results here, effects of glacial–interglacial cycles on genetic diversity pattern were not detected across seven tree species (Milesi et al. 2024).

Within the Cantabrian Mountains, we detected a pronounced pattern of isolation-by-distance, and marked differentiation among three genetic groups, found respectively in Central-Eastern Cantabrian Mountains (CE-CAN), and in Western Cantabrian Mountains around locality Angliru (W-CAN1), and around locality La Cueva (W-CAN2). Split times among populations aligned in an east-to-west gradient, with older splits for population pairs that include more eastern populations. Split times ranged between 150 kya and present, mostly overlapping the last glacial episode and the current interglacial. This suggests a colonization of the Cantabrian Mountains, or a range expansion, that started from a region around the Alps around 200 kya, and progressed westward in Northern Spain through the last glacial period. This pattern is also consistent with more recent gene flow among Western compared to Eastern groups, although we found little evidence of gene flow in our analyses of population structure. Our demographic reconstruction cannot rule out that these populations might have inhabited other regions at the time they split from each other, and then shifted their range to the Cantabrian Mountains more recently, however the clear east-to-west cline in split times suggests a single range expansion wave. In CE-CAN, negative drift rates suggested admixture with populations from the Alps. Between these geographic regions, *A. alpina* is also found in the Pyrenees (not sampled in this study), and these populations might have acted as a stepping stone in the colonization of the Cantabrian Mountains. Future sampling of populations in the Pyrenees will be crucial to resolve admixture between the Alps and CE-CAN, and more generally, the history of the species across Europe. Finally, our results support recent split times between populations in close proximity, and a widespread decline in effective population size in the recent past. These signatures suggest that populations became increasingly fragmented since the LGM, in coincidence with increasing average temperatures across Europe in the present interglacial.

### Candidate genes with signatures of positive selection have diverse physiological functions and may be involved in adaptation to abiotic stress

The demographic model that results from our inference suggests that the colonization of the Cantabrian Mountains happened during a glacial phase, when climatic conditions were likely colder and moister at lower altitude in Northern Spain, compared to present. In the current interglacial, since the last glacial maximum, temperatures have been rising on average, and especially in the Cantabrian Mountains the growing season has become longer and warmer, and summer has become dryer, compared to the rest of the European range. Because *A. alpina* is adapted to Arctic-alpine environments, the increasing temperatures in the current interglacial may have resulted in novel selective pressures. These new challenging conditions might have driven the evolution of new combinations of traits that together increased fitness in warm and dry conditions.

Enriched GO terms among genes in the top 0.5% CLR peaks and the candidate genes with the strongest selection signatures revealed three major functional associations: hormone regulation (e.g. *SC5D*, *GA20OX4*; GO:0010358, GO:0010075), RNA metabolism and splicing (e.g. *BRR2C*, *SDN2*; GO:0000381, GO:0045944), and lipid- and cell wall component metabolism (e.g. *KAS2*, *CASP*, *HPGT3*; GO:0001676, GO:0035652, GO:0042538). This suggests that broad physiological pathways that might affect multiple plant traits are involved in adaptation. Furthermore, some ontologies were associated with selection signatures only on the ancestral, or on the terminal branches, and others were associated with both. The GO terms enriched on the ancestral branches included developmental processes (GO:0010075, GO:0046580, GO:0010358) and nutrient acquisition (GO:0071281, GO:0031670), different from the terminal branches on which multiple enriched GOs were involved in energy metabolism (GO:0001676, GO:0042775, GO:0009150). Both the ancestral and terminal branches were enriched for GO terms associated with abiotic stress responses. On the ancestral branches, these included responses to reactive oxygen species (ROS) (GO:0071452, GO:0046283), and on the terminal branches they included responses to hyperosmotic stress (GO:0042538), calcium-mediated signaling (GO:0050848, GO:1901019), which plays a key role in rapid stress responses, and long-chain fatty acid metabolism (GO:0001676), which is linked to cuticle biosynthesis. Additionally, enriched GO terms related to RNA metabolism included transcriptional initiation (GO:0051123, GO:0045944) on the ancestral branches, and mRNA splicing processes (GO:0000381) on the terminal branches. These results suggest that positive selection on the ancestral branches may have affected growth related phenotypes, nutrient responses, and adaptation to different light intensities, which might have been driven by shifts in altitude during glacial/interglacial cycles, and during the initial colonization of the Cantabrian Mountains. More recent selection in populations from the Cantabrian Mountains, however, likely involved adaptation to water deficiency.

Sixteen genes with selection signatures were directly associated with drought-related GO terms (response to water deprivation and heat). Among these, eleven were on terminal branches, and five on the ancestral branch, suggesting that selection on drought responses was recent. The most prominent candidate gene was *NAC055*, at which we found a 3bp insertion and a TE, which might change the function of the gene, and which are candidate genetic variants for adaptation to drought. The insertion was within a sequence motif at the C-terminus of the gene, which is shared by at least two other stress responsive NAC transcription factors (Tran et al. 2004), and which has been demonstrated to possess transactivation activity (Bu et al. 2008). Interestingly, *NAC055* and another candidate gene, *ZHD11*, are both induced by drought, salinity, and ABA, and overexpression lines have increased drought tolerance (Tran et al. 2004, 2007). Furthermore, they have been shown to cooperatively bind to and induce transcription of the gene *EARLY RESPONSIVE TO DEHYDRATION STRESS 1* (*ERD1*), which is downstream in the ABA independent drought response pathway (Tran et al. 2007). This suggests that positive selection may target specific transcription factor hubs at different regulatory nodes.

Finally, selection signatures were associated with many more genes with various functions, which suggests that selection was multivariate, and that various other traits may have been involved in adaptation. All the genes and genetic variants mentioned here are candidates that might have been involved in adaptation to climatic changes, however the causative mutations and their adaptive value remain to be validated.

### A strong vernalization requirement and short flowering duration contribute to adaptation to drought in a perennial plant

The adaptive value of flowering regulation is established for annual plants, but it may differ in perennials that can revert to vegetative growth and do not necessarily senesce after flowering (Wang et al. 2009). This can be linked to a trade-off between maximizing the reproductive output in the current growing season and maximizing survival until the next growing season and future reproduction. The reversion to vegetative growth is mediated by cycles in the expression of genes that can regulate both the time of flowering onset and the time of flowering arrest, such as *PEP1*, *MAF8.1*, and *MAF8.3* (Wang et al. 2009; Madrid et al. 2021), as well as genes cycling in opposition, such as *COOLAIR* and *VIN3* (Castaings et al. 2014). These pleiotropic effects contribute to the negative correlation between the time of flowering onset and flowering duration which has been observed across plants (Hendry and Day 2005), and specifically in *A. alpina* (Wunder et al. 2023). Accessions from Cantabrian Mountains have a strong requirement for vernalization to flower, and they flower for a short duration after vernalization, compared to other European populations that largely vary in flowering behavior (Wunder et al. 2023). Because early flowering is a derived trait in annual *A. thaliana* that can evolve through loss-of-function mutations at core genes involved in the vernalization pathway (Johanson et al. 2000; Stinchcombe et al. 2004; Méndez-Vigo et al. 2011; Zhang and Jiménez-Gómez 2020; Fulgione et al. 2022), it is possible that a vernalization requirement was present in ancestral populations in Brassicaceae. In this scenario, the strong vernalization requirement might be ancestral in *A. alpina*, and might have remained conserved in populations from Cantabrian Mountains through stronger stabilizing selection than in other European regions. Positive selection may have further contributed to the evolution of this trait, by strengthening the ancestral vernalization requirement. Consistent with selection on flowering regulation, three genes in the vernalization pathway that regulate *FLC* expression, *VIP4*, *VIL1*, and *FRL1*, emerged as candidates to regulate flowering in the Cantabrian Mountains.

At *FRL1* we detected ancient divergence between two alternative haplotypes with a cluster of six non-synonymous mutations near the C-terminus of the gene, although we did not detect a classic signature of selection based on *3P-CLR*. One of the haplotypes was fixed in Cantabrian Mountains and it segregated at low frequency in the Alps, which suggests a local, soft sweep from standing genetic variation in the Cantabrian Mountains. In *A. thaliana*, *frl1* mutants flower very early, similar to *fri* mutants (Michaels et al. 2004). FRL1 is thought to stabilize the FRI-C complex, and it binds to FRI at its N-terminus, and to SUF4 (Choi et al. 2011), possibly at its C-terminus. *SUF4* binds to the promoter of *FLC* and likely has a crucial role on regulating *FLC* expression and flowering. After a cold treatment, FRI-C forms nuclear condensates and is sequestered from the *FLC* promoter, and flowering is enabled (Zhu et al. 2021). Because we did not observe any high-impact mutation at *FRL1*, we hypothesize that the two alternative alleles are functional, and that they may differ in their effect of stabilizing FRI-C. Consistent with an effect of *FRL1* on flowering regulation, the two haplotypes were significantly associated with flowering time in our greenhouse experiment, independent from the region of origin of each accession. Overall, the polymorphism at *FRL1* was ancient, its origin largely predated the colonization of Cantabrian Mountains (at most as ancient as the split from populations in the Alps, 198-230 kya), and the genealogies associated with it were more balanced than expected, with long internal branches and short external branches. These results suggest that variation in flowering behavior evolved under balancing selection in perennial *A. alpina*, perhaps because of spatially or temporally varying selection, mediated by alternative, functional alleles at *FRL1*. In contrast, in *A. thaliana* and other annual species, the adaptive value of flowering regulation is often associated with positive selection for early flowering mediated by nonsense mutations at *FRI*, and rarely at *FLC*, which confer an escape strategy (Johanson et al. 2000; Stinchcombe et al. 2004; Méndez-Vigo et al. 2011; Austen et al. 2017; Zhang and Jiménez-Gómez 2020; Fulgione et al. 2022). Alternatively, it is possible that the polymorphism at *FRL1* originated through introgression of one of the haplotypes from a related species. Similar cases of adaptive introgression have been reported in the genus *Arabidopsis* (Arnold et al. 2016; Baduel et al. 2018), however the genus *Arabis* is not considered a species complex with rampant evidence of introgression among species. Furthermore, we did not find the two haplotypes in seven related *Arabis* species or in *A. thaliana*, but both were present in outcrossing *A. alpina* in South-Eastern Europe. This clade is partially reproductively isolated from the populations studied here, and is considered a different species by some authors (Petrén et al. 2023). This observation is consistent with an ancient, balanced polymorphism at *FRL1*, however it does not rule out an origin through introgression.

Here, we argue that a strong vernalization requirement and a short duration of flowering align into a strategy characterized by flowering avoidance during summer. In the first growing season after germination, plants with a vernalization requirement are unlikely to flower, especially when the growing season is short, as in Arctic-alpine environments. In the following years, all plants readily flower after winter, and plants with a short flowering duration arrest flowering before mid- to late summer. We note that genotype by environment (GxE) interactions may affect these scenarios, however the short duration of flowering of accessions from the Cantabrian Mountains was previously confirmed also for plants growing *in situ* (Wunder et al. 2023), and during our visits at the field sites. Overall, variation in flowering behavior in a perennial plant results in alternative seasonal strategies that mainly differ in whether plants flower in mid- to late summer or not, both in the first growing season and in the following years. When summers are long, warm, and relatively dry, as in Cantabrian Mountains, avoiding processes that require high water consumption, like flowering, may be close to a dehydration escape or avoidance strategy (Volaire 2018). Counter-intuitively, while the principal escape strategy in annuals is associated with early flowering, the closest counterpart in a perennial is associated with late flowering and with a strong vernalization requirement.

### Concluding remarks

The study of plants with an Arctic-alpine distribution can contribute to our understanding of how plants adapt to abiotic stress, and can reveal possible responses to a warming climate. Our study reveals ancient divergence in the perennial, Arctic-alpine herb *A. alpina* from the Cantabrian Mountains in Northern Spain. The inference of the demographic history of these populations suggests an east-to-west range expansion that started around 200 kya from the Alps and proceeded with the colonization of Northern Spain during the last glacial period. Since the LGM, in the current interglacial, we find evidence of population fragmentation, possibly mediated by warming temperatures. This might have resulted in new selective pressures for the evolution of a drought-related trait syndrome, especially in the Cantabrian Mountains where the climate is warm and dry in summer. Among the genes with a signature of selection involved in drought responses and growth, we identified *SC5D* and *NAC055* as the strongest candidates for future molecular validation. Furthermore, we identified an ancient polymorphism at the gene *FRL1* which may contribute to variation in vernalization requirement and flowering duration. We argue here, that the combination of a strong vernalization requirement and a short duration of flowering in *A. alpina* populations from the Cantabrian Mountains results in drought escape, by avoiding increased water stress due to flowering during the warm, dry summer. Conversely, plants from other regions of the range vary between early, late, and no onset of flowering, possibly because of relaxation of purifying selection, or because of spatially or temporally varying selection. Resolving the adaptive value of variation in flowering behavior in these populations is an avenue for future research. Overall, our study suggests that the combination of two different evolutionary processes, positive selection at drought response genes and the maintenance of ancestral variation in flowering phenology, is a possible avenue for the evolution of new, synergistic trait combinations, or new trait syndromes.

## Materials and methods

### Plant material, short reads sequencing and variant calling

We collected cuttings and seeds from 211 individual *A. alpina* plants across 14 populations in the Cantabrian Mountains in Northern Spain, and from 215 individual plants across 15 populations in the French Alps (online supplementary material, Table S1). Some of these accessions were sourced from previous studies (Wunder et al. 2023; Tjeng et al. 2025). We extracted DNA and sequenced whole genomes with 150 bp paired end short reads nano ball sequencing at the Beijing Genomics Institute (BGI). More details on DNA extraction and sequencing can be found in the online Supplementary material.

SNP calling was performed using the GATK pipeline v4.2.0 (Depristo et al. 2011). Read groups were assigned, adapters were soft clipped, duplicates were marked and reads were aligned to the reference genome *Arabis alpina v5.1* (Jiao et al. 2017). GATK haplotypecaller was used to call SNPs and short indels by performing local realignments. To avoid biases in variant frequencies, genotypes were called for each sample separately. Genotypes with genome-wide average depth of 10× or less were excluded from the analyses. SNPs with depth less than 5× and genotype quality less than 30 were set to missing values. Indels were removed and only biallelic sites were retained. We identified and filtered multicopy genomic regions in the samples from the Cantabrian Mountains and from the Alps, after filtering sites with more than 30% missing values, using default settings in ParaMask (Tjeng et al. 2025). Furthermore, we retained only uniquely mapping genomic regions inferred with SNPable (https://lh3lh3.users.sourceforge.net/snpable.shtml), using K-mer length of 150 and intermediate stringency (r=0.5). Finally, we improved the annotation of the reference genome, and predicted the effects of genetic variants (described in online Supplementary material).

### Genomic diversity and population structure analyses

We obtained a set of unrelated individuals by applying a greedy search algorithm to the empirical distribution of pairwise differences, in comparison to expected values assuming known relatedness status (detail in online Supplementary material). We calculated $\theta_{\pi }$, $\theta_{W}$, Tajima’s D, and $F_{IS}$ using custom scripts available in GitHub (details in online Supplementary material). We only used populations with sample sizes greater than four, because low sample numbers result in noisy estimates. To assess correlations between genetic, geographic, and environmental distances we used Mantel and partial Mantel tests with 9,999 permutations (details in online Supplementary material). For the principal coordinate analysis (PCoA), we performed ordination of 1 - IBS distances in a 2-dimensional space using the metric scaling implemented in PLINK v1.9.0 (Chang et al. 2015). We inferred the proportion of shared genetic ancestry for two to 20 ancestry groups using ADMIXTURE v1.3.0 (Alexander et al. 2009), after pruning singleton SNPs, SNPs with more than 10% missing genotypes, SNPs in linkage disequilibrium ($r^{2}>0.2$), and related individuals. The best number of ancestry groups was chosen based on the lowest average cross-validation (CV) error among 10 replicates (details in online Supplementary material).

### Demographic inference

To reconstruct genome-wide genealogies and calculate coalescent rates, we used the software Relate v.1.2.2 (Speidel et al. 2019). We inferred the ancestral states for genome-wide SNPs on the basis of an alignment between the *A. alpina* reference genome, and the genome of the outgroup species *A. montbretiana* (described in online Supplementary material). Relate can analyze populations with variable sample sizes, therefore we only discarded population ES15 which had a single sample. We imputed and phased genomic data in two steps (described in online Supplementary material), and we pruned annotated genes. We accounted for missing data and preserved the SNP densities by adjusting the distances between SNPs, and we accounted for inbreeding by selecting randomly one haplotype per individual. We assumed a mutation rate of $7\times 10^{-9}$ (Ossowski et al. 2010), a generation time of 1.5 years per generation (Laenen et al. 2018), an initial effective population size of $2\times 10^{5}$ (based on estimates of genomic diversity, $\theta_{W}$), and we used a scaled recombination map from crosses (online Supplementary material). Confidence intervals were constructed from 100 bootstraps, by randomly re-sampling 5 Mbp regions, and constructing pseudo chromosomes of the same size as the actual chromosomes. For the inference of split times, we used a threshold of 0.5 for the relative cross-coalescent rate, after fitting a piecewise cubic polynomial function to the point estimates and 95% confidence intervals on a logarithmic time scale using the smooth.spline function from the stats v4.0.4 package in R. To test an east-to-west colonization of the Cantabrian Mountains we modeled split times in dependence of the longitude of each population pair, and we accounted for non-independence among pairs using permutations (online Supplementary material).

### Genomic scans for selection signatures

We identified signatures of positive selection with *3P-CLR* (Racimo 2016). As focus clades, we used different combinations of the three major clusters from the Cantabrian Mountains, W-CAN1, W-CAN2, and ES17 (as a non-admixed representative of CE-CAN; details in online Supplementary material). We repeated the analyses with populations from the Alps, or from CE-CAN (excluding ES17) as outgroups (details in online Supplementary material). For each combination of clades, we excluded sites with more than 10% missing values within groups. We calculated drift rates on the basis of F3 statistics after pruning multicopy regions which are known to bias these estimates (Tjeng et al. 2025), regions that do not map uniquely inferred with SNPable, and regions two kbp up- and down-stream of genes. We calculated CLR scores in genomic windows of 0.00025 Morgans, which corresponds to a median window size of 111.1 kbp. Because the CLR scores are calculated locally and do not bias the analysis genome-wide, we included genes and multicopy regions in the analyses, and we manually assessed the main CLR peaks. As suggested for *3P-CLR*, SNPs with extreme frequencies in the outgroup (minor allele frequency <0.01, singletons, and (n-1)-tons) were excluded. The number of sampled SNPs was set to 100, and the minimum distance between focal SNPs to 20. CLR scores where normalized by mean and standard deviation for every branch and set of samples. The genes that overlapped the upper 0.5% tail of CLR scores in every branch were used for gene ontology enrichment analyses, and the genes that overlapped the top five peaks of every group of branches were manually analyzed. Selection scans with *XP-EHH* are described in online Supplementary material. We did not use selection scans based on population differentiation (e.g. $F_{ST}$), because *A. alpina* populations are highly diverged, which implies high variance in $F_{ST}$ across the genome. We did not use genotype-environment associations because these approaches are confounded by long-range LD due to strong population structure.

### Inference of synonymous and non-synonymous divergence, polymorphism, and diversity

We quantified the rate ratio of non-synonymous to synonymous substitutions ($d_{N}/d_{S}$), and polymorphisms ($p_{N}/p_{S}$), and the ratios of non-synonymous to synonymous average pairwise differences ($\theta_{\pi_{n}}/\theta_{\pi_{s}}$) across all 28,745 annotated genes in the genome using custom scripts available in GitHub. We defined as substitutions and polymorphisms respectively the mutations that were fixed derived, or segregating, in all individuals in a group (211 individuals for the Cantabrian Mountains, 215 for the Alps), compared to the outgroup *A. montbretiana*. To calculate the number of n-fold degenerate sites, we used degenotate (Mirchandani et al. 2024). To test for differences in $d_{N}/d_{S}$ and $p_{N}/p_{S}$ between populations in the Cantabrian Mountains and in the Alps, we used a logistic regression for correlated data (with a Generalized Estimating Equations model, GEE). To test for differences in $\theta_{\pi_{n}}/\theta_{\pi_{s}}$, we used a bootstrap approach. More details can be found in online Supplementary material.

### Gene ontology (GO) enrichment analyses

The enrichment of GO categories were tested using topGO (Alexa et al. 2006) with the elim algorithm and Fisher’s exact test. We only considered GO categories that included a minimum of two genes and more than one locus, and an enrichment with a minimum significance level of 0.5% without multiple hypothesis testing.

## Author contributions

A.F., G.C. and B.T. conceived and designed the study. B.T. performed most of the analyses. M.M. developed and performed the analyses of non-synonymous and synonymous variation. H.B.G. performed the greenhouse experiment. J.Z. annotated the reference genome. C.A.B., A.D.L., B.T. and J.W. collected the plants, A.D.L. and B.T. propagated them and extracted DNA for sequencing. B.T. and A.F. wrote the manuscript, with contributions from all authors.

## Supplementary Material

msag212_Supplementary_Data

## Data Availability

All code used in data analyses and visualization is available in GitHub at: https://github.com/Fulgione-group/Spanish_Arabis_Alpina. The sequencing dataset supporting the conclusions of this article is available in the European Nucleotide Archive (ENA) under the accession numbers PRJEB73825 and PRJEB96335. The new annotation of the reference genome is available at http://www.arabis-alpina.org/. All data supporting conclusions that are not included in the online Supplementary material, the scripts for analyses, and the vcf file are available in Zenodo at: https://zenodo.org/records/21170632.
