# Supplementary material for: Adaptation to seasonal drought in Arabis alpina is linked to the demographic history and climatic changes since the last glacial maximum
Source: Mol Biol Evol. 2026 Aug 22;43(9):msag212. doi: 10.1093/molbev/msag212 (PMC13551622; doi:10.1093/molbev/msag212)
Supplement: Supplementary file 1 [file msag212_supplementary_data.pdf]

Supplementary Materials

Adaptation to seasonal drought in *Arabis alpina* is linked to the demographic history and climatic changes since the last glacial maximum

Bastiaan Tjeng[1], Mehak Mehak[1], Helene Bråten Grindeland[1], Jasmin Zohren[1], Andrea Dalla Libera[1], Jörg Wunder[2], Carlos Alonso-Blanco[3], George Coupland[1] & Andrea Fulgione[1,*]

August 17, 2026

[1]: Max Planck Institute for Plant Breeding Research, Carl-von-Linne-Weg 10, 50829, Cologne, Germany

[2]: Nunhems Netherlands BV, Napoleonsweg 152, 6083 AB Nunhem, The Netherlands

[3]: Centro Nacional de Biotecnologia (CNB), Consejo Superior de Investigaciones Científicas (CSIC), Madrid 28049, Spain

***Corresponding author:** Andrea Fulgione, e-mail: fulgione@mpipz.mpg.de

Supplementary Text

Plant material, short reads sequencing and variant calling

We collected cuttings and seeds from 211 individual *A. alpina* plants across 14 populations in the Cantabrian Mountains in Northern Spain, and from 215 individual plants across 15 populations in the French Alps (Supplementary Table S1). Some of these accessions were sourced from previous studies (Wunder et al. 2023; Tjeng et al. 2025). We rooted the cuttings in the greenhouse, and we extracted DNA from 100 mg of young leaves and meristems, following the DNeasy Plant Mini Kit protocol with minor adjustments. Whole-genome sequencing with short reads was performed at the Beijing Genomics Institute (BGI) for all samples. DNA concentration was quantified by fluorometer or microplate reader (e.g. qubit fluorometer, invitrogen). Sample integrity and purity were detected by agarose gel electrophoresis (concentration of agarose gel: 1%, voltage: 150 V, electrophoresis time: 40 minutes). 1 μg of genomic DNA was randomly fragmented with covaris. The DNA fragments were selected by magnetic beads to an average size of 200-400 bp. Fragments were end repaired and then 3' adenylated. Adaptors were ligated to the ends of these 3' adenylated fragments. Fragments were amplified with PCR using the adaptors from the previous step. PCR products were purified by the magnetic beads. The double stranded PCR products were heat denatured and circularized by the splint oligo sequence. The single strand circle DNA (ssCir DNA) were formatted as the final library. The library was amplified with phi29 to make DNA nanoball (DNB). The DNBs were load into the patterned nanoarray and paired-end 150 base pairs reads were generated with combinatorial probe-anchor synthesis (cPAS).

Analysis of genetic diversity and population structure

To obtain a set of unrelated individuals, we calculated the average pairwise difference for any second-degree relationship (C_{pd}). This calculation was based on the average within population pairwise differences ($\overline{pd}$) and mean heterozygosity ($\overline{het}$), assuming an outcrossing rate of $t = 0.2$, which reflects conservative estimates from mother-offspring relationships reported by Toräng et al. (2017). Considering a grandmother-grandchild relationship, with either two generations of selfing, one generation of selfing followed by outcrossing (or vice versa), or two generations of outcrossing, the formula is as

follows:

$$C_{pd} = (1-t)^2 \cdot 0.5 \cdot \overline{het} + 2t(1-t) \cdot \big(0.25 \cdot \overline{het} + 0.5 \cdot \overline{pd}\big) + t^2 \cdot \big(0.125 \cdot \overline{het} + 0.75 \cdot \overline{pd}\big)$$

We then applied a greedy search algorithm to retain pairs of individuals with pairwise differences higher than that estimate.

To estimate diversity and F_{IS}, we used the set of unrelated individuals, and we did not prune for linkage. To calculate F_{IS}, we used populations with sample sizes (≥ 10) to avoid high error rates in allele frequency estimates, and we calculated Nei's Ho and Hs per site, across populations (Nei 1987). We then calculated F_{IS} genome-wide and in windows of 10,000 SNPs as $1 - \sum Ho / \sum Hs$. To account for missing values, we randomly projected the number of genotypes down to 90% of the total sample size per population.

To assess correlations between genetic distances, geographic distances, and environmental distances we used Mantel and partial Mantel tests implemented in the R package vegan. We calculated genetic distances as pairwise differences among individuals and geographic distances by using the haversine method implemented in the Python package haversine. We calculated environmental distances as Canberra distances between individuals based on 93 climatic variables. To test for IBD, we used genetic distance as the response matrix, geographic distance as the predictor matrix, and environmental distance as the conditioning matrix. To test for IBE, we used environmental distance as the predictor matrix and geographic distance as the conditioning matrix. The Mantel statistic was based on Pearson's correlations with 9,999 permutations.

In the ADMIXTURE analysis, the most representative replicate for each number of ancestry groups, and the best alignment of clusters between the most representative replicates was chosen after post processing the Q-matrices with pong (Behr et al. 2016).

Identification of ancestral states based on a whole-genome alignment of the outgroup *Arabis montbretiana* to the *A. alpina* reference genome

We aligned the *Arabis montbretiana* genome, accessed at NCBI with accession number PRJNA258048, to the *A. alpina* reference genome *v*5.1 (Jiao et al. 2017) using lastz aligner *v*1.04.15 (Harris 2007),

kentools $v302$ (Nassar et al. 2023) and GenomeAlignmentTools (Sharma and Hiller 2017; Osipova et al. 2019). Each chromosome of the *A. montbretiana* reference was split in ten segments, and each segment was aligned to the *A. alpina* reference. We used lastz parameters $-gap = 400, 30$, $-gappedthresh = 3000$, and $-inner = 2200$. The alignments of each segment were chained using axtChain from kentools with parameter $-linearGap = medium$. To fill missing alignments in repetitive regions we used the RepeatFiller script from GenomeAlignmentTools with lastz parameters $O = 400$, $E = 30$, $L = 3000$, $T = 1$ and $H = 2200$. The patchChain script in GenomeAlignmentTools was used to perform highly sensitive local realignment after chaining. Segments before and after realignment were merged, chained again and netted. The axt alignment files were converted into a fasta file with the sequence coordinates of the *A. alpina* reference genome.

Phasing genomic data for demographic inference

For demographic analyses, we phased genomic data in two steps. First, we prephased haplotypes using information of sequencing read pairs in the bam files with WhatsHap $v.1.1$ and including phase-set block tags (- *-tag=PS*; Martin et al. 2023). Next, the data set was divided into two subsets, one for the Cantabrian Mountains populations and one for the populations from the Alps. For each subset we pruned sites with more than 20% missing genotypes, and multicopy regions identified with ParaMask (Tjeng et al. 2025). We then jointly phased the data, and imputed missing genotypes on each sample set using the HMM-based approach implemented in shapeit $v.4.2$, while keeping existing phase-set blocks from WhatsHap with an error prior of 10^{-4} (- *-use-PS 0.0001*; Delaneau et al. 2019). In shapeit, we used a recombination map from an F2 crossing population between individuals from population FR01 (Wötzel 2016). Recombination rates were scaled to $(1 - F_{IS})$ to adjust for inbreeding (Nordborg 2000), using the average F_{IS} that we inferred in previous analyses. We then merged the subsets and pruned annotated genes and their flanking regions (2kbp).

Estimating phasing switch discordance rates between heterozygote calls based on short reads and on long reads (PacBio Hifi)

We assessed the accuracy of our phasing procedure by comparing phasing results from short reads and from long reads sequencing for two accessions (ES03-14 and ES04-14, sequenced with PacBio Hifi long reads and processed as in (Tjeng et al. 2025)). The rationale was that long reads would capture linked alleles at greater genomic distances than short reads, and therefore provide a meaningful benchmark. First, we mapped the long reads to the reference genome using minimap2 $v2.30 - r1287$ and default parameters (Li 2018). For each accession, we only kept primary alignments, and we called SNPs from the aligned long reads with bcftools $v1.15.1$ *mpileup* and *call* commands, and with default settings. We excluded SNPs that overlapped multicopy regions or low mappability regions, SNPs with quality < 30 or sequencing depth < 5, we polarized SNPs to the outgroup *A. montbretiana*, and we phased heterozygote SNPs with WhatsHap $v.1.1$ (Martin et al. 2023). Finally, we used the *compare* command in WhatsHap, to quantify the differences in phasing (switch rates) between short reads and long reads sequencing for the same accession, across genome-wide heterozygote sites.

For the accession ES03-14, we identified 32,998 and 181,341 heterozygote sites from short and long reads, respectively. For the accession ES04-14, we identified 139,981 and 445,408 heterozygote sites from short and long reads, respectively. We note that the differences in the numbers of heterozygote sites are likely due to our stricter filtering on short reads, which includes a filter for sites with $\leq 20\%$ missing genotypes based on individual genotype quality, and to differences in the two methods for SNP calling. However, both long and short read data suggested lower heterozygosity in ES03-14 compared to ES04-14. Respectively for ES03-14 and ES04-14, we estimated that genome-wide, 371 and 1,380 SNPs were phased differently in short reads and in long reads, out of a total of 28,918 and 169,958 heterozygous SNPs (1.28% and 0.81%, respectively). Considering that the true associations among alleles are unknown, and that heterozygosity is low due to inbreeding, these values indicate strong agreement between methods and suggest good phasing accuracy.

The Cantabrian Mountains are characterized by warmer temperatures year-around, and lower precipitation in summer, compared to other collection sites in Europe

Because our reconstruction of the history of *A. alpina* in the Cantabrian Mountains is consistent with an important role of glacial and interglacial periods, we characterized the climatic conditions at collection sites across Europe. Monthly temperature and precipitation records were obtained from `https://www.worldclim.org/` at a resolution of 30" for the period 1970–2000 for all collection sites in the Cantabrian Mountains and in the Alps, as well as for sites in Scandinavia described in Wunder et al. (2023). The monthly Aridity Index at the collection sites was sourced from Zomer et al. (2022). The average monthly temperatures at the sites in the Cantabrian Mountains were above freezing year-around (Supplementary Fig. S5a). In contrast, average monthly temperature in the Alps and in Scandinavia was 5.4°C and 8.8°C lower than in the Cantabrian Mountains, and it was below freezing from November to April and from October to April, respectively. Annual precipitation was highest in the Alps (mean: 1322.0 mm), followed by the Cantabrian Mountains (mean: 1151.1 mm), and lowest in Scandinavia (mean: 890.8 mm). Moreover, the seasonal course of precipitation largely differed among regions (Supplementary Fig. S5b). In Scandinavia, precipitation peaked in summer (July to October), while in the Alps and in the Cantabrian Mountains, precipitation was at a minimum in summer (June to September). As a result of warmer temperatures year-around, and lower precipitation in summer, the sites in Northern Spain are more arid in summer, especially between mid-June and September (Supplementary Fig. S5c).

Comparison of edaphic conditions between the French Alps and the Cantabrian Mountains.

For every population site in the French Alps and in Cantabrian Mountains, we obtained topsoil data from the nearest sampling location in the LUCAS survey 2018 (Fernandez et al. 2022) and retained only soil data from sites located within 10 km of our sampling sites. This resulted in ten unique sampling sites for the populations in Cantabrian Mountains, and two unique sampling sites for the populations in the Alps. The two sampling sites in the Alps had a pH of 6.8, whereas pH values at

the sites in Cantabrian Mountains ranged from 3.8 to 6.3 (mean = 4.8), indicating more acidic soils. Heavy metal data were only available at the NUTS2 level, which corresponds to administrative regions spanning on average approximately 15,000 km^2, and therefore did not provide a suitable proxy for local soil conditions at the population sites. Nevertheless, the Cantabrian Mountains are characterized by extensive carbonate-hosted Zn-Pb mineralization and numerous iron deposits, and have a long history of mining that dates back to Roman times, which can create highly differential local edaphic environments (Morellón et al. 2025; Gómez-Lende et al. 2025).

At four populations in the Cantabrian Mountains (ES17, ES03, ES06, and ES04), we installed soil moisture loggers (Odyssey Xtreem MPSMT) to measure soil water saturation and soil temperature over a period between 2021 and 2023. Probes were buried at a depth of 5–10 cm, at a distance from *A. alpina* plants of at most 1–2 m, completely covered by soil, and programmed to record measurements every two hours. Raw sensor values were converted to soil water saturation percentages using Dataflow Xpert software and a linear calibration assuming a silt loam soil texture. Silt loam was selected because it was the most common soil texture among nearby sampling locations according to the LUCAS 2018 soil survey. The calibration offset was adjusted so that measured values did not exceed 100% saturation.

All monitored populations experienced warm and dry conditions during summer, particularly from mid-July to late August (Supplementary Fig. S6). Across all sites, 2022 was generally drier than 2023. Among the four populations, ES17 experienced the longest and most pronounced seasonal drought, with soil moisture beginning to decline as early as May and soil water saturation falling below the estimated wilting point (23%) in both years (Supplementary Fig. S6a). The wilting point was calculated for a silt loam soil from volumetric water content at wilting point and soil porosity following Saxton and Rawls (2006). Soil water saturation at ES04 also fell below the wilting point during 2022, but not during 2023 (Supplementary Fig. S6d). In contrast, soil water saturation remained above the wilting point throughout both years at ES03 and ES06 (Supplementary Fig. S6b–c).

Improvement of the annotation of the *A. alpina* reference genome

We constructed an NCBI assembly BLAST database using RMBlast *v*2.10.0+ and performed a *de novo* search for repetitive elements with RepeatModeler *v*2.0.1 (Flynn et al. 2020). This library was

fed into RepeatMasker $v4.1.2 - p1$ (http://www.repeatmasker.org) to generate a GFF file with alignments of the query and the matching repeats. The repeat GFF file and a generic TE library distributed with the MAKER pipeline (Cantarel et al. 2008) were used for internal repeat masking during the structural genome annotation. This was performed with MAKER $v3.01.03$ with the proteomes of *A. alpina* (UniProt identifier: UP000029120) and *A. thaliana* (UniProt identifier: UP000006548), as well as a previous annotation file of *A. alpina* (Jiao et al. 2017) supplied as evidence files. The gene predictor Augustus was used as part of the pipeline, with the *A. thaliana* data set as HMM training set. Additionally, we employed the deep neural network gene prediction software Helixer $v0.3.0$ (Stiehler et al. 2021; Holst et al. 2023). Post-processing of the gene annotations was conducted with the AGAT suite $v1.0.0$ (Dainat 2023), which resulted in manually curated GFF3 files. To further enhance the annotation, we integrated homology information using Orthorbb $v2.2$ from GenomeAnnotation (https://github.com/darencard/GenomeAnnotation) with default parameters. Functional annotation, including gene ontologies, was performed using EggNOG $v2.1.9$ with default settings (Huerta-Cepas et al. 2019; Cantalapiedra et al. 2021).

To predict the effects of genetic variants, we first constructed a novel database for the reference genome by utilizing the new genome annotation GTF file. Then, we annotated a VCF file that included outgroup data from the whole-genome alignment of *A. montbretiana* for SNPs, and outgroup data from short reads of *A. montbretiana* for indels, using the newly created database described above, and the default settings in SNPeff $v5.2.a$ (Cingolani et al. 2012).

Assessing the correlation between split times and longitude

In order to assess the direction of colonization of Cantabrian Mountains, we calculated correlation coefficients between split times among population pairs and different measures of geographic location. Populations in Cantabrian Mountains are mostly spread along longitude (Fig. 1a), have a clear signal of isolation by distance, and are related to populations from the Alps. As a consequence of this, our main hypothesis was that colonization might have happened in a single range expansion wave from east to west. If this was the case, we would expect subsequent population splits from the east to the west, specifically as a function of the longitude of the easternmost population in each pair of populations.

For instance, the most ancient split would separate the easternmost population from all others, and it would therefore be independent from the location of the westernmost population in each pair, and linked to the location of the easternmost population. The second most ancient split would separate the population located just west of the easternmost from all others to the west of it, and it would therefore be independent from the location of the westernmost population in each pair, but linked with the location of the second-to-easternmost population. The same rationale applies to all other population splits.

To quantify these relationships, we calculated the Pearson correlation coefficient between split times and the longitude of the easternmost population in each population pair, with the longitude of the westernmost population, and with the longitude of the midpoint. To account for the fact that pairwise comparisons are not independent from each other, we performed 10^6 permutations of the population coordinates, while keeping the same coordinates for a given population across all pairs. Empirical p-values were estimated as the proportion of permutations that resulted in a Pearson correlation coefficient greater or equal to the empirical estimate.

As expected under an east-to-west colonization scenario, the correlation was strongest between split times and the longitude of the easternmost population ($r = 0.836$, p-value=$2.70 \cdot 10^{-5}$), it was not significant with the longitude of the westernmost population ($r = 0.140$, p-value=0.47), and it was significant but weaker with the midpoint ($r = 0.687$, p-value=$2.56 \cdot 10^{-4}$). This last residual correlation is expected, since the midpoint retains part of the information on the longitude of the easternmost population, which is, in this scenario, the real driver of the correlation.

Selection scans with *3P-CLR*

For the *3P-CLR* analysis, we used six combinations of populations, each consisting of two subject populations and one outgroup (Supplementary Table S7). As subject populations, we used the three Cantabrian Mountains structure groups, combined in all possible pairs (three combinations in total), and used the French population as the outgroup. However, the resulting negative drift rate estimates between CE-CAN and the W-CAN1 or W-CAN2 groups (–0.066 and –0.063, respectively) indicated potential admixture, violating the tree like assumptions of the method. To address this, we replaced

the pooled CE-CAN group with only the westernmost CE-CAN population, ES17, which is genetically closest to the W-CAN groups and also the most distinct within CE-CAN. This adjustment resulted in positive drift rate estimates between ES17 and both W-CAN groups (Supplementary Table S7). Given the high divergence between Cantabrian Mountains and French populations, we also performed additional *3P-CLR* analyses using CE-CAN as the outgroup. Specifically, we used the full CE-CAN group, including ES17, as outgroup for the analyses focused on W-CAN1 and W-CAN2, and we used CE-CAN excluding ES17 as the outgroup when analyzing combinations that included W-CAN1 or W-CAN2 with ES17 as subject populations.

Across sets, we detected a weak negative correlation between the CLR scores and the extent of overlap with multicopy regions. For instance, correlation coefficients ranged between -0.036 and -0.066 for set2 and for multicopy regions detected in the populations from Cantabrian Mountains, and between -0.026 and -0.053 for multicopy regions detected in populations from the Alps. This indicates that multicopy regions tend to reduce, and not to enhance signatures of positive selection in this data set, and are therefore unlikely to result in false positives. This is consistent with previous results that reported that multicopy regions generally have the effect of homogenizing genetic variation between populations (Tjeng et al. 2025).

In the analyses with CE-CAN as outgroup (excluding ES17), in the terminal branches that lead to W-CAN1, W-CAN2, and ES17, the top five CLR peaks overlapped genes with roles in sterol metabolism, RNA processing, abiotic stress responses, hormone- and light- mediated growth responses, and translational capacity (Supplementary Table S9). The upper 0.5% CLR peaks overlapped genes enriched in membrane trafficking and secretion, lipid and pigment metabolism, proteostasis, calcium signaling, and RNA splicing (Supplementary Table S10). On the branch ancestral to W-CAN1, W-CAN2 and ES17, the top five CLR peaks overlapped genes linked to hormone transport and metabolism, ion homeostasis, defense, abiotic stress responses, and meiotic recombination (Supplementary Table S9). The top 0.5% CLR peaks on the ancestral branch were significantly enriched in genes related to responses to environmental cues, including nutrient and oxidative stress, as well as developmental processes (Supplementary Table S10).

Selection scans with XP-EHH

To infer signatures of recent positive selection, we performed a cross-population extended haplotype homozygosity analysis (*XP-EHH*). We used phased but unpolarized SNPs, excluding multicopy regions because they may lead to erroneous phasing. One haplotype was randomly selected per individual to avoid bias due to recent inbreeding from plant propagation. *XP-EHH* scores, defined as the log-ratio of integrated haplotype homozygosity (*iHH*) between populations, were calculated for the three pairwise comparisons among WC-CAN1, WC-CAN2, and CE-CAN using selscan $v1.2.0$ with $--max-gap500000$ to prevent integration over large physical gaps and $--trunc-ok$ to allow truncation at chromosome tails (Szpiech and Hernandez 2014). We defined SNPs within the most extreme 0.5% tails of the *XP-EHH* distribution as candidate SNPs. We then compared the overlap of these SNPs with the genes located within the highest *3P-CLR* peaks located on the terminal branches of W-CAN1 and W-CAN2 (Supplementary Table S9).

The top *XP-EHH* peak (*XP-EHH* = 2.57) overlapped the top *3P-CLR* peak, and the *SC5D* locus. At this locus, integrated haplotype homozygosity was up to 13.76-fold higher in W-CAN2 than in CE-CAN (Supplementary Fig. S9). We further found overlap between the two selection scans at the *BRRC2* (*XP-EHH* = 1.15) and *VIP4* (*XP-EHH* = 1.27) loci on the terminal W-CAN2 branch and at the *CYP57* locus on the terminal W-CAN1 branch (*XP-EHH* = 1.11). At one *3P-CLR* peak comprising *AA7G12830*, *SDN2*, two *DET1* paralogs, *APX*, *AA7G12880*, *NLE1*, and *LAX2*, phased SNPs were unavailable, which prevented the assessment of the overlap between *XP-EHH* and *3P-CLR*. At the *TWD40-2* locus and the *3P-CLR* peak comprising *PCR4*, *CASP*, *IRL2*, and *ASPG1*, we did not find an overlap between extreme *XP-EHH* and *3P-CLR* scores.

Comparison between *3P-CLR* scores in empirical data and in simulations

In the *3P-CLR* analyses, we used the upper 0.5% empirical quantile of CLR scores as a threshold to focus on the genomic regions with the strongest evidence for selection. We evaluated this empirical threshold by calculating *3P-CLR* scores on genetic variation simulated assuming the demographic history of the studied populations inferred with Relate (Supplementary Fig. S7). We note that *3P-CLR* already accounts for the main effect of demography by using estimated drift rates to model neutral

allele frequency changes through time, however these simulations can account for more complexities in the demographic history. Specifically, we simulated a 50 Mb genome in 80 replicates with the coalescent simulator msprime $v1.2.0$ (Baumdicker et al. 2022), assuming a demographic model that included all populations that we used in the empirical *3P-CLR* analysis (Supplementary Fig. S7). Effective population sizes and split times were set to the median values estimated with Relate among populations within each group (see section on demographic inference). Changes in effective population size through time were modeled as piecewise exponential changes between time periods set by our demographic analysis in Relate. We used the same mutation rate that we used for demographic inference (7×10^{-9}; (Ossowski et al. 2010)), and a recombination rate of 1.01×10^{-8} per bp and per generation, which corresponds to our estimated genome-wide recombination rate scaled by $1 - F_{IS}$. We calculated *3P-CLR* scores for each replicate of the simulations, each set of populations (the same six sets described in Supplementary Table S7), and each branch, using the same parameters as in our empirical *3P-CLR* analysis. Note that the simulations are neutral (i.e., genetic variation evolves with no selection), but they account for the demographic history, therefore the distribution of *3P-CLR* scores from the simulations can be used as a null distribution for the *3P-CLR* analysis on empirical data.

To test our empirical CLR threshold, we compared raw and standardized *3P-CLR* scores in empirical data and from the simulations (Supplementary Table S8). When focusing on the raw *3P-CLR* scores, the 0.5% upper quantile in empirical data was in most cases in line with the 0.5% upper quantile in the simulations. In particular, the empirical threshold was always more conservative (higher *3P-CLR* score) than the 1% upper quantile in the simulated data, except for the terminal branches of population ES17 in sets 5 and 6, in which it was still more conservative than the 5% upper quantile in the simulations. When focusing on the standardized *3P-CLR* scores, which should be more robust to inaccuracies in model specifications, the 0.5% upper quantile in empirical data was also in most cases in line with the 0.5% upper quantile in the simulations. In particular, the empirical threshold was always more conservative than the 1% upper quantile of the standardized simulated *3P-CLR* scores, except for the ancestral branches in sets 1, 2, and 3, in which it was still more conservative then the 5% upper quantile, and close to the 1% upper quantile. In conclusion, comparisons to simulations that

account for demography indicate that our empirical threshold is largely in line with a conservative threshold between 0.5% and 1% under neutrality, and never less conservative than a 5% threshold.

Finally, we compared the quantiles of the standarized *3P-CLR* scores in empirical data and in simulations (Supplementary Fig. S8). In most tested branches, empirical data had an enrichment of high *3P-CLR* scores (had a heavy tail at high scores) in comparison with neutral simulations, except for the ancestral branch in sets with the populations from the Alps as outgroup (sets 1, 2 and 3), and for the terminal branch of W-CAN1 in a single set (set4, one out of four sets that included this group). This enrichment shows that empirical data has a stronger evidence for selection compared to neutral simulations, in most branches analyzed. The lack of a signal on the ancestral branch between Cantabrian Mountains and the Alps is likely due to ancient divergence, which is known to result in reduced sensitivity of the test (Racimo 2016).

Correlations between allele dosage at the 3bp insertion at *NAC055* and the aridity index

After we identified a 3bp insertion at *NAC055* as a candidate genetic variant with a selection signature and possibly related to drought responses, we tested whether this variant was correlated with the aridity index at the collection site. We repeated the analysis on the full data set (n=211), and after excluding populations ES02, ES10, and ES15 (n = 200), which were collected at sites with micro climates very distinct from the surrounding climate. In particular, ES02 was on a roadside and shaded by buildings in an otherwise warm and dry environment, ES10 was inside a cave, likely moister and cooler than the surroundings, and ES15 was on a roadside and near buildings in a canyon near a small river.

The aridity index in July was a highly significant predictor of allele dosage at the 3bp insertion, based on a binomial regression with four principal coordinates from genome-wide structure as covariates (on the whole data set, Wald p-value $= 6.26 \times 10^{-5}$; excluding populations with putatively distinct microclimate, Wald p-value $= 5.14 \times 10^{-4}$; Supplementary Fig. S10). Similarly, the allele dosage at the 3bp insertion was a highly significant predictor of the aridity index in July, based on a linear regression with four principal coordinates from genome-wide structure as covariates (on the whole data set, Wald p-value $= 4.48 \times 10^{-9}$; excluding populations with putatively distinct microclimate, Wald p-value =

5.78×10^{-15}).

Inference of non-synonymous and synonymous divergence, polymorphism, and diversity

We analyzed the rate ratios of non-synonymous to synonymous substitutions (d_N/d_S) and polymorphism (p_N/p_S) in the populations from the Cantabrian Mountains in comparison with the rate ratios in the populations from the Alps, using a logistic regression for correlated data (with a Generalized Estimating Equations model, GEE). This approach is commonly used to account for correlations among dichotomous observations, e.g. for longitudinal data in which repeated observations on the same individual can be correlated. In our case, this approach accounts for the mutations that originated in the population ancestral to Cantabrian Mountains and to the Alps, and that can contribute to polymorphism and to divergence in both regions. In other words, when a mutation originated earlier than the split, and is present in both regions, the test considers the two data points, one in Cantabrian Mountains and one in the Alps, as two correlated observations of the same mutation. In particular, we used the region of origin as binary independent variable (encoded as an indicator variable that takes value 0 for Cantabrian Mountains, and 1 for the Alps), and the type of the mutation as binary dependent variable (non-synonymous mutations encoded as 0, and synonymous mutations encoded as 1). The standard logistic function is then:

$$Y = P(synonymous) = \frac{e^{\beta_0+\beta_1 x}}{1+e^{\beta_0+\beta_1 x}} \tag{1}$$

And the odds are defined as:

$$Odds = \frac{p(synonymous)}{1-p(synonymous)} = e^{\beta_0+\beta_1 x} \tag{2}$$

Where Y is a measure of the probability of observing a synonymous mutation as a function of x, the indicator variable for the region of origin. β_0 and β_1 are the vertical intercept and the slope of the log-odds as a function of x, respectively. β_1 provides information about how the odds to observe a synonymous differ as a function of the region of origin (Cantabrian Mountains or the Alps), for each

gene. Specifically, β_1 is the logit coefficient, or the log of the odds ratio of observing a synonymous mutation in the Alps, in comparison to observing a synonymous mutations in the Cantabrian Mountains. Importantly, the odds of a synonymous mutation is a statistic closely related to the d_N/d_S or p_N/p_S ratios, if we assume that observed frequencies can be used to estimate probabilities.

For the d_N/d_S and p_N/p_S analyses we included filters for minimum SNP counts. In particular, in the p_N/p_S analysis we eliminated all genes with 10 or less SNPs within each region (Cantabrian Mountains and Alps), and genes that had less than three SNPs for any of the two functional categories (synonymous and non-synonymous). Furthermore, we required genes to have more than 10 SNPs in total that are private to one or to the other region (i.e., the sum of private variation in Cantabrian Mountains and in the Alps needs to be larger than 10). In the d_N/d_S analysis, we eliminated all genes with 5 or less SNPs within each region (Cantabrian Mountains and Alps), and genes that had less than three SNPs for any of the two functional categories (synonymous and non-synonymous). Furthermore, we required genes to have more than 5 SNPs in total that are private to one or to the other region (i.e., the sum of private variation in Cantabrian Mountains and in the Alps needs to be larger than 5).

In order to account for the effects of demography on the test statistics, we used an estimate of genome-wide odds ratios as a benchmark for the test. For this, we averaged the odds ratios (e^{β_1}) across all genes to obtain a genome-wide odds ratio. We then converted the genome-wide odds ratio into a genome-wide logit coefficient. To identify genes with significantly different rate ratios in Cantabrian Mountains and in the Alps, we tested for a difference between the logit coefficient of each gene, and the genome-wide logit coefficient (which is mostly influenced by demography), using the Wald test.

To test for differences in non-synonymous to synonymous diversity between populations from Cantabrian Mountains and from the Alps, we measured for each gene the average density of pairwise differences at non-synonymous (θ_{π_n}) and at synonymous sites (θ_{π_s}). For each gene, we calculated θ_π at every non-synonymous and synonymous variant site, separately for Cantabrian mountains and for the Alps, obtaining four distributions (the four combinations of non-synonymous and synonymous sites, in Cantabrian mountains and in the Alps). We eliminated genes with fewer than two SNPs in each region, genes with zero synonymous SNPs in any region, and genes with zero non-synonymous SNPs in both regions. From these distributions, we randomly sampled with replacement the same number

of genetic variants observed at the specific gene in the empirical data, and we repeated this bootstrap 100 times. For every one of these bootstraps, we calculated the difference between Cantabrian mountains and the Alps, in the ratio of non-synonymous to synonymous pairwise differences, $\theta_{\pi_n}/\theta_{\pi_s}$. The genome-wide average of this statistic is mostly influenced by the demographic history, and in particular by long-term effective population size, and it was 0.990. Single genes may deviate from the general pattern due to differences in the strength or mode of selection. Therefore, we tested each gene for a difference in the statistic between the Cantabrian Mountains and the Alps, in comparison with the genome-wide average difference, using a Z-test.

Across all these analyses, we used the Benjamini Hochberg FDR procedure to correct for multiple hypothesis testing.

Assessing haplotype lengths at *FRL1* and genome-wide

The two genealogies that overlapped the six non-synonymous mutations at *FRL1* were separated by the root split into two haplotype groups of 163 and 272 samples. This division extended into neighbouring genealogies, with 97.7% agreement between consecutive root splits, covering a total of eleven genealogies across 11,592 bp. To evaluate whether this haplotype length was unusual, we screened genome-wide for similarly balanced root-split segments. These were defined as segments with at least 152 descendants on the smaller side of the root split and at least 95% agreement between consecutive root splits. Before this scan, we excluded any tree overlapping low-density regions, defined as intervals with at least 1,000 bp between neighbouring SNPs and an adjusted-distance/physical-distance ratio below 0.01. Among 63,189 similarly balanced segments, only 2.74×10^{-4} were longer than the focal haplotype segment.

Genotyping variation at *FRL1* in public data sets

In order to test whether the two haplotypes at *FRL1* are found in other populations or species, we applied a targeted genotyping approach to a public data set (Gutiérrez-Valencia et al. 2022). For this, read groups were assigned, adapter sequences were soft-clipped, duplicate reads were marked, and reads were aligned to the *Arabis alpina* v5.1 reference genome (Jiao et al. 2017). Genotypes at the six

non-synonymous SNPs that distinguish the two *FRL1* haplotypes were called using the *mpileup* and *call* commands of bcftools with a minimum mapping quality threshold of 30 (`-q 30`). Genotypes were treated as missing if the difference between the second-best and best phred-scaled genotype likelihood (PL) scores was less than 30.

Assessing variation in flowering behavior in greenhouse conditions

We grew 71 accessions from the Cantabrian Mountains and 55 from the Alps in a controlled greenhouse experiment with and without vernalization to characterize their flowering behavior. In the experiment without vernalization, the accessions were grown in four replicates with a randomized block design, with one replicate per block, and with a photoperiod of 16 h of light, and 8 h of darkness (16/8). In the experiment with vernalization, the accessions were grown in 3 replicates for 7 weeks in greenhouse conditions, then they were shifted to four degrees for 12 weeks, and then shifted again to greenhouse conditions with a 16/8 photoperiod. In both experiments, seeds were stratified in cold and moist conditions for five days, then planted in 9x9 cm pots with a standard soil mix. Germination time was uniform across accessions. The time of flowering onset (flowering time) was scored as the number of days between sowing and the opening of the first flower, and was scored twice per week. In the experiment without vernalization, plants that had not flowered by the end of the experiment at 250 days after sowing, were considered non-flowering. Plants that died before flowering were excluded from the experiment. Accessions for which one replicate died were included in the analyses, and for no accession more than one replicate died in the experiment.

Consistent with previous results (Wunder et al. 2023), most of the accessions from the Cantabrian Mountains were unable to flower without vernalization in our experiment (65 accessions out of 71, or 91.6%, did not flower in any replicate). A single accession flowered in all four replicates, and five additional accessions flowered in at least one replicate, and they all flowered very late (on average 194.2 days from sowing). In comparison, all of the 55 accessions from the Alps flowered in at least one replicate, and most flowered in all replicates (51 accessions out of 55, or 92.7%). Accessions from the Alps varied widely between early and late onset of flowering (range: 48 to 247 days; on average after 115.8 days excluding non-flowering plants). After vernalization, all replicates of all accessions

from the Cantabrian Mountains and from the Alps flowered, and did so mostly within 20-30 days from vernalization.

Assessing the relationship between the haplotype at *FRL1* and flowering time

We tested for an association between the six non-synonymous substitutions at the C-terminus of *FRL1* (the haplotype at *FRL1*) and flowering time without vernalization scored in the greenhouse experiment on all the 126 accessions from populations from the Cantabrian Mountains and from the Alps. For this, we fitted a Cox proportional-hazards model using the function *coxph* implemented in the R package 'survival'. We used the haplotype at *FRL1* as the predictor variable and the time of flowering onset as the outcome variable. We repeated the analysis with and without the region of origin (the Alps vs the Cantabrian Mountains) as covariate.

Accessions with the *FRL1* haplotype fixed in the populations from the Cantabrian Mountains had a 96% lower hazard of flowering compared to the accessions with the alternative haplotype (z = -18.73, p-value = 2.0×10^{-16}). After including the region of origin as covariate, accessions with the haplotype fixed in the Cantabrian populations had a 72% lower hazard of flowering compared to the accessions with the alternative haplotype (z = -7.41, p-value = 1.27×10^{-13}).

Supplementary Tables

Table S1: *Arabis alpina* accessions used in this study and their metadata, including sample ID, country, location, coordinates, collection date, and Source (CL = Coupland lab; FL = Fulgione Lab; CAB = Carlos Alonso-Blanco). The sample ID is composed of the population ID, accession number, a letter indicating collection as cuttings (C) or seeds (S), and the number of generations selfed before sequencing.

ID	Country	Location	Coordinates	Collection date	Source
ES01-001-C-00	Spain	Léon - Puerto de la Cubilla	N42.9931°, W5.9100°	8/9/2020	FL
ES01-001-S-00	Spain	Léon - Puerto de la Cubilla	N42.9931°, W5.9103°	8/9/2020	FL
ES01-004-C-00	Spain	Léon - Puerto de la Cubilla	N42.9935°, W5.9102°	8/9/2020	FL
ES01-005-C-00	Spain	Léon - Puerto de la Cubilla	N42.9929°, W5.9094°	8/9/2020	FL
ES01-007-C-00	Spain	Léon - Puerto de la Cubilla	N42.9932°, W5.9103°	8/9/2020	FL
ES01-009-C-00	Spain	Léon - Puerto de la Cubilla	N42.9927°, W5.9101°	8/9/2020	FL
ES01-010-C-00	Spain	Léon - Puerto de la Cubilla	N42.9928°, W5.9102°	8/9/2020	FL
ES01-011-C-00	Spain	Léon - Puerto de la Cubilla	N42.9927°, W5.9103°	8/9/2020	FL
ES01-012-C-00	Spain	Léon - Puerto de la Cubilla	N42.9926°, W5.9107°	8/9/2020	FL
ES01-014-S-00	Spain	Léon - Puerto de la Cubilla	N42.9927°, W5.91155°	8/9/2020	FL
ES01-015-S-00	Spain	Léon - Puerto de la Cubilla	N42.9927°, W5.9115°	8/9/2020	FL
ES01-016-S-00	Spain	Léon - Puerto de la Cubilla	N42.9937°, W5.9123°	8/9/2020	FL
ES01-017-C-00	Spain	Léon - Puerto de la Cubilla	N42.9933°, W5.9110°	8/9/2020	FL
ES01-018-C-00	Spain	Léon - Puerto de la Cubilla	N42.9934°, W5.9109°	8/9/2020	FL
ES01-019-C-00	Spain	Léon - Puerto de la Cubilla	N42.9901°, W5.9059°	8/9/2020	FL
ES01-020-S-00	Spain	Léon - Puerto de la Cubilla	N42.9901°, W5.9144°	8/9/2020	FL
ES01-046-S-01	Spain	Asturias - Puerto de la Cubilla	N42.9936°, W5.9089°	9/13/2010	CL
ES01-049-S-01	Spain	Asturias - Puerto de la Cubilla	N42.9936°, W5.9089°	3/27/2015	CL
ES01-059-S-01	Spain	Asturias - Puerto de la Cubilla	N42.9936°, W5.9089°	8/6/2013	CL
ES01-065-S-01	Spain	Asturias - Puerto de la Cubilla	N42.9936°, W5.9089°	4/1/2015	CL
ES01-074-S-01	Spain	Asturias - Puerto de la Cubilla	N42.9936°, W5.9089°	9/1/2012	CL
ES01-084-S-01	Spain	Asturias - Puerto de la Cubilla	N42.9936°, W5.9089°	9/25/2014	CL
ES01-103-S-01	Spain	Asturias - Puerto de la Cubilla	N42.9936°, W5.9089°	4/1/2015	CL
ES01-108-S-01	Spain	Asturias - Puerto de la Cubilla	N42.9918°, W5.9081°	9/2/2012	CL
ES02-001-S-00	Spain	Asturias - Puerto de Pajares	N42.9923°, W5.7604°	8/8/2020	FL
ES02-002-S-00	Spain	Asturias - Puerto de Pajares	N42.9921°, W5.7605°	8/8/2020	FL
ES02-003-S-01	Spain	Asturias - Puerto de Pajares	N42.9924°, W5.7597°	9/2/2012	CL
ES02-004-S-00	Spain	Asturias - Puerto de Pajares	N42.9932°, W5.7450°	8/8/2020	FL
ES02-005-C-00	Spain	Asturias - Puerto de Pajares	N42.9930°, W5.7440°	8/8/2020	FL
ES03-001-S-00	Spain	Asturias - Angliru	N43.2276°, W5.9367°	8/7/2020	FL
ES03-002-C-00	Spain	Asturias - Angliru	N43.2279°, W5.9373°	8/7/2020	FL
ES03-003-C-00	Spain	Asturias - Angliru	N43.2293°, W5.9379°	8/7/2020	FL
ES03-005-C-00	Spain	Asturias - Angliru	N43.2298°, W5.9385°	8/7/2020	FL
ES03-006-C-00	Spain	Asturias - Angliru	N43.2298°, W5.9385°	8/7/2020	FL
ES03-007-C-00	Spain	Asturias - Angliru	N43.2299°, W5.9410°	8/7/2020	FL
ES03-008-C-00	Spain	Asturias - Angliru	N43.2300°, W5.9412°	8/7/2020	FL
ES03-038-S-01	Spain	Asturias - Angliru	N43.2298°, W5.9390°	3/27/2015	CL
ES03-009-C-00	Spain	Asturias - Angliru	N43.2298°, W5.9423°	8/7/2020	FL
ES03-010-C-00	Spain	Asturias - Angliru	N43.2304°, W5.9438°	8/7/2020	FL
ES03-011-C-00	Spain	Asturias - Angliru	N43.2303°, W5.9440°	8/7/2020	FL
ES03-035-S-01	Spain	Asturias - Angliru	N43.2298°, W5.9390°	9/6/2012	CL
ES03-012-C-00	Spain	Asturias - Angliru	N43.2305°, W5.9443°	8/7/2020	FL
ES03-014-C-00	Spain	Asturias - Angliru	N43.2310°, W5.9443°	8/7/2020	FL
ES03-015-C-00	Spain	Asturias - Angliru	N43.2319°, W5.94504°	8/7/2020	FL
ES03-016-C-00	Spain	Asturias - Angliru	N43.2319°, W5.9450°	8/7/2020	FL
ES03-037-S-01	Spain	Asturias - Angliru	N43.2298°, W5.9390°	2/20/2014	CL
ES03-017-C-00	Spain	Asturias - Angliru	N43.2218°, W5.9429°	8/7/2020	FL
ES03-018-C-00	Spain	Asturias - Angliru	N43.2219°, W5.9430°	8/7/2020	FL
ES03-036-S-01	Spain	Asturias - Angliru	N43.2298°, W5.9390°	7/3/2014	CL
ES03-019-C-00	Spain	Asturias - Angliru	N43.2220°, W5.9431°	8/7/2020	FL
ES03-020-C-00	Spain	Asturias - Angliru	N43.2298°, W5.9390°	8/7/2020	FL
ES03-021-C-00	Spain	Asturias - Angliru	N43.2231°, W5.9446°	8/7/2020	FL
ES03-022-C-00	Spain	Asturias - Angliru	N43.2233°, W5.9437°	8/7/2020	FL
ES03-023-C-00	Spain	Asturias - Angliru	N43.2234°, W5.9435°	8/7/2020	FL
ES03-024-S-01	Spain	Asturias - Angliru	N43.2298°, W5.9390°	9/25/2014	CL
ES03-026-S-00	Spain	Asturias - Angliru	N43.2281°, W5.9345°	8/7/2020	FL

ES03-028-C-00	Spain	Asturias - Angliru	N43.2315°, W5.9373°	8/7/2020	FL
ES03-029-C-00	Spain	Asturias - Angliru	N43.2317°, W5.9373°	8/7/2020	FL
ES03-030-S-00	Spain	Asturias - Angliru	N43.2316°, W5.9370°	8/7/2020	FL
ES03-031-C-00	Spain	Asturias - Angliru	N43.2292°, W5.9385°	9/8/2021	FL
ES03-032-C-00	Spain	Asturias - Angliru	N43.2291°, W5.9386°	9/8/2021	FL
ES03-033-C-00	Spain	Asturias - Angliru	N43.2288°, W5.9371°	9/8/2021	FL
ES03-047-S-01	Spain	Asturias - Angliru	N43.2298°, W5.9390°	2/20/2014	CL
ES03-048-S-01	Spain	Asturias - Angliru	N43.2298°, W5.9390°	2/20/2014	CL
ES03-055-S-01	Spain	Asturias - Angliru	N43.2298°, W5.9390°	2/20/2014	CL
ES03-034-S-01	Spain	Asturias - Angliru	N43.2298°, W5.9390°	2/20/2014	CL
ES04-001-C-00	Spain	Asturias - Lagos de Saliencia	N43.0559°, W6.1016°	8/10/2020	FL
ES04-002-C-00	Spain	Asturias - Lagos de Saliencia	N43.0518°, W6.1036°	8/10/2020	FL
ES04-003-C-00	Spain	Asturias - Lagos de Saliencia	N43.0517°, W6.1036°	8/10/2020	FL
ES04-004-C-00	Spain	Asturias - Lagos de Saliencia	N43.0517°, W6.1037°	8/10/2020	FL
ES04-005-C-00	Spain	Asturias - Lagos de Saliencia	N43.0533°, W6.1014°	8/10/2020	FL
ES04-006-C-00	Spain	Asturias - Lagos de Saliencia	N43.0533°, W6.1014°	8/10/2020	FL
ES04-008-C-00	Spain	Asturias - Lagos de Saliencia	N43.0519°, W6.1040°	8/10/2020	FL
ES04-009-C-00	Spain	Asturias - Lagos de Saliencia	N43.0519°, W6.1041°	8/10/2020	FL
ES04-010-C-00	Spain	Asturias - Lagos de Saliencia	N43.0519°, W6.1038°	8/10/2020	FL
ES04-011-C-00	Spain	Asturias - Lagos de Saliencia	N43.0512°, W6.1052°	8/10/2020	FL
ES04-012-C-00	Spain	Asturias - Lagos de Saliencia	N43.0512°, W6.1053°	8/10/2020	FL
ES04-013-C-00	Spain	Asturias - Lagos de Saliencia	N43.0513°, W6.1053°	8/10/2020	FL
ES04-014-C-00	Spain	Asturias - Lagos de Saliencia	N43.0514°, W6.1052°	8/10/2020	FL
ES04-015-C-00	Spain	Asturias - Lagos de Saliencia	N43.0514°, W6.1051°	8/10/2020	FL
ES04-016-C-00	Spain	Asturias - Lagos de Saliencia	N43.0516°, W6.1051°	8/10/2020	FL
ES04-017-C-00	Spain	Asturias - Lagos de Saliencia	N43.0513°, W6.1041°	8/10/2020	FL
ES04-018-C-00	Spain	Asturias - Lagos de Saliencia	N43.0513°, W6.1040°	8/10/2020	FL
ES04-019-C-00	Spain	Asturias - Lagos de Saliencia	N43.0512°, W6.1040°	8/10/2020	FL
ES04-020-C-00	Spain	Asturias - Lagos de Saliencia	N43.0514°, W6.1053°	8/10/2020	FL
ES04-021-S-00	Spain	Asturias - Lagos de Saliencia	N43.0512°, W6.1025°	8/10/2020	FL
ES04-022-C-00	Spain	Asturias - Lagos de Saliencia	N43.0512°, W6.1025°	8/10/2020	FL
ES04-041-S-01	Spain	Asturias - Lagos de Saliencia	N43.0513°, W6.1042°	8/5/2013	CL
ES04-024-S-00	Spain	Asturias - Lagos de Saliencia	N43.0511°, W6.1026°	8/10/2020	FL
ES04-025-C-00	Spain	Asturias - Lagos de Saliencia	N43.0511°, W6.1023°	8/10/2020	FL
ES04-026-C-00	Spain	Asturias - Lagos de Saliencia	N43.0509°, W6.1024°	8/10/2020	FL
ES04-027-C-00	Spain	Asturias - Lagos de Saliencia	N43.0510°, W6.1023°	8/10/2020	FL
ES04-028-C-00	Spain	Asturias - Lagos de Saliencia	N43.0510°, W6.1022°	8/10/2020	FL
ES04-029-C-00	Spain	Asturias - Lagos de Saliencia	N43.0510°, W6.1026°	8/10/2020	FL
ES04-042-S-01	Spain	Asturias - Lagos de Saliencia	N43.0513°, W6.1042°	2/20/2014	CL
ES04-030-C-00	Spain	Asturias - Lagos de Saliencia	N43.0512°, W6.1038°	8/10/2020	FL
ES04-031-C-00	Spain	Asturias - Lagos de Saliencia	N43.0511°, W6.1039°	8/10/2020	FL
ES04-032-C-00	Spain	Asturias - Lagos de Saliencia	N43.0511°, W6.1037°	8/10/2020	FL
ES04-033-C-00	Spain	Asturias - Lagos de Saliencia	N43.0510°, W6.1040°	8/10/2020	FL
ES04-043-S-01	Spain	Asturias - Lagos de Saliencia	N43.0513°, W6.1042°	3/27/2015	CL
ES04-034-C-00	Spain	Asturias - Lagos de Saliencia	N43.0510°, W6.1038°	8/10/2020	FL
ES04-035-C-00	Spain	Asturias - Lagos de Saliencia	N43.0514°, W6.1053°	9/7/2021	FL
ES04-036-C-00	Spain	Asturias - Lagos de Saliencia	N43.0513°, W6.1015°	9/7/2021	FL
ES04-037-C-00	Spain	Asturias - Lagos de Saliencia	N43.0498°, W6.1026°	9/7/2021	FL
ES04-057-S-01	Spain	Asturias - Lagos de Saliencia	N43.0513°, W6.1042°	nan	CL
ES04-038-C-00	Spain	Asturias - Lagos de Saliencia	N43.0519°, W6.1087°	9/7/2021	FL
ES04-039-C-00	Spain	Asturias - Lagos de Saliencia	N43.0519°, W6.1087°	9/7/2021	FL
ES04-045-S-01	Spain	Asturias - Lagos de Saliencia	N43.0513°, W6.1042°	2/20/2014	CL
ES04-046-S-01	Spain	Asturias - Lagos de Saliencia	N43.0513°, W6.1042°	2/20/2014	CL
ES04-050-S-01	Spain	Asturias - Lagos de Saliencia	N43.0513°, W6.1042°	2/20/2014	CL
ES04-051-S-01	Spain	Asturias - Lagos de Saliencia	N43.0513°, W6.1042°	9/4/2012	CL
ES04-052-S-01	Spain	Asturias - Lagos de Saliencia	N43.0513°, W6.1042°	4/20/2015	CL
ES04-064-S-01	Spain	Asturias - Lagos de Saliencia	N43.0513°, W6.1042°	9/5/2012	CL
ES04-072-S-01	Spain	Asturias - Lagos de Saliencia	N43.0513°, W6.1042°	8/5/2013	CL
ES05-001-C-00	Spain	Asturias - El Coto	N43.0828°, W6.2359°	8/11/2020	FL
ES05-002-S-00	Spain	Asturias - El Coto	N43.0826°, W6.2363°	8/11/2020	FL
ES05-003-C-00	Spain	Asturias - El Coto	N43.0833°, W6.2378°	8/11/2020	FL
ES05-004-C-00	Spain	Asturias - El Coto	N43.0833°, W6.2378°	8/11/2020	FL
ES05-005-C-00	Spain	Asturias - El Coto	N43.0835°, W6.2380°	8/11/2020	FL
ES05-006-C-00	Spain	Asturias - El Coto	N43.0800°, W6.2348°	8/11/2020	FL
ES05-007-C-00	Spain	Asturias - El Coto	N43.0800°, W6.2348°	8/11/2020	FL
ES05-009-S-00	Spain	Asturias - El Coto	N43.0779°, W6.2342°	8/11/2020	FL
ES05-010-C-00	Spain	Asturias - El Coto	N43.0775°, W6.2339°	8/11/2020	FL

ES05-011-S-00	Spain	Asturias - El Coto	N43.0828°, W6.2365°	8/11/2020	FL
ES05-012-S-00	Spain	Asturias - El Coto	N43.0828°, W6.2364°	8/11/2020	FL
ES05-013-S-00	Spain	Asturias - El Coto	N43.0828°, W6.2363°	8/11/2020	FL
ES05-014-S-00	Spain	Asturias - El Coto	N43.0830°, W6.2371°	8/11/2020	FL
ES05-016-S-00	Spain	Asturias - El Coto	N43.0832°, W6.2377°	8/11/2020	FL
ES05-017-C-00	Spain	Asturias - El Coto	N43.0834°, W6.2380°	8/11/2020	FL
ES05-018-C-00	Spain	Asturias - El Coto	N43.0835°, W6.2387°	8/11/2020	FL
ES05-019-C-00	Spain	Asturias - El Coto	N43.0799°, W6.2347°	8/11/2020	FL
ES05-020-C-00	Spain	Asturias - El Coto	N43.0798°, W6.2346°	8/11/2020	FL
ES05-021-C-00	Spain	Asturias - El Coto	N43.0779°, W6.2343°	8/11/2020	FL
ES05-022-C-00	Spain	Asturias - El Coto	N43.0779°, W6.2344°	8/11/2020	FL
ES05-023-S-00	Spain	Asturias - El Coto	N43.0780°, W6.2346°	8/11/2020	FL
ES06-001-S-01	Spain	Asturias - Puerto de la Cubilla	N43.0007°, W5.9269°	9/10/2014	CL
ES06-002-S-01	Spain	Asturias - Puerto de la Cubilla	N43.0007°, W5.9269°	7/2/2014	CL
ES06-003-C-00	Spain	Asturias - Puerto de la Cubilla	N42.9993°, W5.9280°	8/9/2020	FL
ES06-004-S-00	Spain	Asturias - Puerto de la Cubilla	N42.9995°, W5.9282°	8/9/2020	FL
ES06-005-S-00	Spain	Asturias - Puerto de la Cubilla	N42.9995°, W5.9282°	8/9/2020	FL
ES06-006-C-00	Spain	Asturias - Puerto de la Cubilla	N42.9998°, W5.9283°	8/9/2020	FL
ES06-007-C-00	Spain	Asturias - Puerto de la Cubilla	N43.0001°, W5.9288°	8/9/2020	FL
ES06-008-C-00	Spain	Asturias - Puerto de la Cubilla	N43.0003°, W5.9288°	8/9/2020	FL
ES06-008-S-01	Spain	Asturias - Puerto de la Cubilla	N43.0007°, W5.9269°	9/18/2014	CL
ES06-009-C-00	Spain	Asturias - Puerto de la Cubilla	N43.0004°, W5.9289°	8/9/2020	FL
ES06-010-S-01	Spain	Asturias - Puerto de la Cubilla	N43.0007°, W5.9269°	6/20/2014	CL
ES06-011-C-00	Spain	Asturias - Puerto de la Cubilla	N43.0026°, W5.9283°	8/9/2020	FL
ES06-012-S-00	Spain	Asturias - Puerto de la Cubilla	N42.9995°, W5.92821°	8/9/2020	FL
ES06-015-C-00	Spain	Asturias - Puerto de la Cubilla	N43.0008°, W5.9274°	9/6/2021	FL
ES06-016-C-00	Spain	Asturias - Puerto de la Cubilla	N43.0007°, W5.9278°	9/6/2021	FL
ES06-017-C-00	Spain	Asturias - Puerto de la Cubilla	N43.0008°, W5.9287°	9/6/2021	FL
ES06-018-C-00	Spain	Asturias - Puerto de la Cubilla	N43.0009°, W5.9285°	9/6/2021	FL
ES06-020-S-01	Spain	Asturias - Puerto de la Cubilla	N43.0007°, W5.9269°	nan	CL
ES06-021-C-00	Spain	Asturias - Puerto de la Cubilla	N43.0017°, W5.9307°	9/6/2021	FL
ES06-022-C-00	Spain	Asturias - Puerto de la Cubilla	N43.0014°, W5.9311°	9/6/2021	FL
ES06-023-C-00	Spain	Asturias - Puerto de la Cubilla	N43.0013°, W5.9312°	9/6/2021	FL
ES06-026-C-00	Spain	Asturias - Puerto de la Cubilla	N42.9989°, W5.9301°	9/6/2021	FL
ES06-041-S-01	Spain	Asturias - Puerto de la Cubilla	N43.0007°, W5.9269°	9/19/2014	CL
ES06-045-S-01	Spain	Asturias - Puerto de la Cubilla	N43.0007°, W5.9269°	7/3/2014	CL
ES06-050-S-01	Spain	Asturias - Puerto de la Cubilla	N43.0007°, W5.9269°	9/25/2014	CL
ES08-001-S-01	Spain	Asturias - Naranjo de Bulnes	N43.2071°, W4.8149°	7/17/2014	CL
ES08-002-S-01	Spain	Asturias - Naranjo de Bulnes	N43.2071°, W4.8149°	7/2/2014	CL
ES08-004-S-01	Spain	Asturias - Naranjo de Bulnes	N43.2071°, W4.8149°	9/25/2014	CL
ES08-005-S-01	Spain	Asturias - Naranjo de Bulnes	N43.2071°, W4.8149°	7/3/2014	CL
ES08-006-S-01	Spain	Asturias - Naranjo de Bulnes	N43.2071°, W4.8149°	9/18/2014	CL
ES08-007-S-01	Spain	Asturias - Naranjo de Bulnes	N43.2071°, W4.8149°	8/10/2009	CL
ES08-008-S-01	Spain	Asturias - Naranjo de Bulnes	N43.2071°, W4.8149°	7/1/2014	CL
ES08-009-S-01	Spain	Asturias - Naranjo de Bulnes	N43.2071°, W4.8149°	8/10/2009	CL
ES08-010-S-01	Spain	Asturias - Naranjo de Bulnes	N43.2071°, W4.8149°	8/10/2009	CL
ES09-001-S-01	Spain	Cantabria - Fuente Dé	N43.1657°, W4.8126°	8/4/2013	CL
ES09-003-S-01	Spain	Cantabria - Fuente Dé	N43.1657°, W4.8126°	7/20/2015	CL
ES09-004-S-01	Spain	Cantabria - Fuente Dé	N43.1490°, W4.8075°	8/4/2013	CL
ES10-001-C-00	Spain	Asturias - Cueva Huerta	N43.1236°, W6.0596°	8/11/2020	FL
ES10-002-C-00	Spain	Asturias - Cueva Huerta	N43.1237°, W6.0586°	8/11/2020	FL
ES10-003-C-00	Spain	Asturias - Cueva Huerta	N43.1237°, W6.0586°	8/11/2020	FL
ES10-004-C-00	Spain	Asturias - Cueva Huerta	N43.1237°, W6.0586°	8/11/2020	FL
ES10-005-S-01	Spain	Asturias - Cueva Huerta	N43.1237°, W6.0586°	8/11/2020	FL
ES15-001-S-00	Spain	Asturias - Entrepeñas	N43.1426°, W5.5792°	8/8/2020	FL
ES17-001-C-00	Spain	Léon - Puebla de Lillo	N43.0098°, W5.2775°	8/12/2020	FL
ES17-002-C-00	Spain	Léon - Puebla de Lillo	N43.0120°, W5.2788°	8/12/2020	FL
ES17-004-C-00	Spain	Léon - Puebla de Lillo	N43.0126°, W5.2792°	8/12/2020	FL
ES17-005-C-00	Spain	Léon - Puebla de Lillo	N43.0126°, W5.2793°	8/12/2020	FL
ES17-006-C-00	Spain	Léon - Puebla de Lillo	N43.0127°, W5.2793°	8/12/2020	FL
ES17-007-C-00	Spain	Léon - Puebla de Lillo	N43.0127°, W5.2794°	8/12/2020	FL
ES17-009-C-00	Spain	Léon - Puebla de Lillo	N43.0095°, W5.2780°	8/12/2020	FL
ES17-010-C-00	Spain	Léon - Puebla de Lillo	N43.0126°, W5.2794°	8/12/2020	FL
ES17-011-C-00	Spain	Léon - Puebla de Lillo	N43.0132°, W5.2794°	8/12/2020	FL
ES17-012-C-00	Spain	Léon - Puebla de Lillo	N43.0094°, W5.2763°	8/12/2020	FL
ES17-014-C-00	Spain	Léon - Puebla de Lillo	N43.0156°, W5.2836°	8/12/2020	FL
ES17-015-C-00	Spain	Léon - Puebla de Lillo	N43.0155°, W5.2835°	8/12/2020	FL

ES17-016-C-00	Spain	Léon - Puebla de Lillo	N43.0155°, W5.2835°	8/12/2020	FL
ES17-017-C-00	Spain	Léon - Puebla de Lillo	N43.0155°, W5.2834°	8/12/2020	FL
ES17-018-C-00	Spain	Léon - Puebla de Lillo	N43.0155°, W5.2834°	8/12/2020	FL
ES23-001-S-00	Spain	Asturias - Puerto de Leitariegos	N43.0016°, W6.4242°	8/15/2020	CAB
ES23-003-S-00	Spain	Asturias - Puerto de Leitariegos	N43.0016°, W6.4242°	8/15/2020	CAB
ES23-005-S-00	Spain	Asturias - Puerto de Leitariegos	N43.0016°, W6.4242°	8/15/2020	CAB
ES23-006-S-00	Spain	Asturias - Puerto de Leitariegos	N43.0016°, W6.4242°	8/15/2020	CAB
ES23-007-S-00	Spain	Asturias - Puerto de Leitariegos	N43.0016°, W6.4242°	8/15/2020	CAB
ES24-001-S-01	Spain	Cantabria - Brañavieja	N43.0397°, W4.3631°	nan	CAB
ES24-002-S-01	Spain	Cantabria - Brañavieja	N43.0397°, W4.3631°	nan	CAB
ES24-004-S-01	Spain	Cantabria - Brañavieja	N43.0397°, W4.3631°	nan	CAB
ES24-005-S-01	Spain	Cantabria - Brañavieja	N43.0397°, W4.3631°	nan	CAB
ES25-001-S-01	Spain	Burgos - Portillo de la Sia	N43.1532°, W3.5699°	nan	CAB
ES25-002-S-01	Spain	Burgos - Portillo de la Sia	N43.1532°, W3.5699°	nan	CAB
ES25-003-S-01	Spain	Burgos - Portillo de la Sia	N43.1532°, W3.5699°	nan	CAB
ES25-004-S-01	Spain	Burgos - Portillo de la Sia	N43.1532°, W3.5699°	nan	CAB
ES25-005-S-01	Spain	Burgos - Portillo de la Sia	N43.1532°, W3.5699°	nan	CAB
ES25-006-S-01	Spain	Burgos - Portillo de la Sia	N43.1532°, W3.5699°	nan	CAB
ES25-007-S-01	Spain	Burgos - Portillo de la Sia	N43.1532°, W3.5699°	nan	CAB
ES25-008-S-01	Spain	Burgos - Portillo de la Sia	N43.1532°, W3.5699°	nan	CAB
ES25-009-S-01	Spain	Burgos - Portillo de la Sia	N43.1532°, W3.5699°	nan	CAB
FR01-001-S-01	France	Savoy - Grand Galibier	N45.0550°, E6.44995°	9/15/2018	CL
FR01-002-S-01	France	Savoy - Grand Galibier	N45.0550°, E6.44995°	9/15/2018	CL
FR01-003-S-01	France	Savoy - Grand Galibier	N45.0559°, E6.44644°	9/15/2018	CL
FR01-004-S-01	France	Savoy - Grand Galibier	N45.0584°, E6.44095°	9/15/2018	CL
FR01-005-S-01	France	Savoy - Grand Galibier	N45.0607°, E6.43910°	9/15/2018	CL
FR01-006-S-01	France	Savoy - Grand Galibier	N45.0636°, E6.43435°	9/15/2018	CL
FR01-007-S-01	France	Savoy - Grand Galibier	N45.0634°, E6.43489°	9/15/2018	CL
FR01-008-S-01	France	Savoy - Grand Galibier	N45.0639°, E6.43389°	9/15/2018	CL
FR01-009-S-01	France	Hautes-Alpes - Col du Galibier	N45.0594°, E6.40532°	9/23/2012	CL
FR01-013-S-01	France	Hautes-Alpes - Col du Galibier	N45.0594°, E6.40532°	9/23/2012	CL
FR01-015-S-01	France	Hautes-Alpes - Col du Galibier	N45.0594°, E6.40532°	nan	CL
FR01-016-S-01	France	Hautes-Alpes - Col du Galibier	N45.0594°, E6.40532°	nan	CL
FR01-018-S-01	France	Hautes-Alpes - Col du Galibier	N45.0594°, E6.40532°	nan	CL
FR01-020-S-01	France	Hautes-Alpes - Col du Galibier	N45.0594°, E6.40532°	nan	CL
FR01-028-S-01	France	Hautes-Alpes - Col du Galibier	N45.0594°, E6.40532°	9/23/2012	CL
FR01-035-S-01	France	Hautes-Alpes - Col du Galibier	N45.0594°, E6.40532°	nan	CL
FR01-036-S-01	France	Hautes-Alpes - Col du Galibier	N45.0594°, E6.40532°	nan	CL
FR01-038-S-01	France	Hautes-Alpes - Col du Galibier	N45.0594°, E6.40532°	nan	CL
FR01-039-S-01	France	Hautes-Alpes - Col du Galibier	N45.0594°, E6.40532°	nan	CL
FR01-040-S-01	France	Hautes-Alpes - Col du Galibier	N45.0594°, E6.40532°	nan	CL
FR01-046-S-01	France	Hautes-Alpes - Col du Galibier	N45.0594°, E6.40532°	nan	CL
FR01-047-S-01	France	Hautes-Alpes - Col du Galibier	N45.0594°, E6.40532°	nan	CL
FR01-055-S-01	France	Hautes-Alpes - Col du Galibier	N45.0594°, E6.40532°	9/23/2012	CL
FR01-062-S-01	France	Hautes-Alpes - Col du Galibier	N45.0594°, E6.40532°	9/23/2012	CL
FR01-070-S-01	France	Hautes-Alpes - Col du Galibier	N45.0594°, E6.40532°	nan	CL
FR01-072-S-01	France	Hautes-Alpes - Col du Galibier	N45.0601°, E6.40347°	9/23/2012	CL
FR01-073-S-01	France	Hautes-Alpes - Col du Galibier	N45.0602°, E6.40344°	9/23/2012	CL
FR01-075-S-01	France	Hautes-Alpes - Col du Galibier	N45.0602°, E6.40344°	nan	CL
FR01-079-S-01	France	Hautes-Alpes - Col du Galibier	N45.0602°, E6.40344°	9/23/2012	CL
FR01-081-S-01	France	Hautes-Alpes - Col du Galibier	N45.0602°, E6.40344°	9/23/2012	CL
FR01-083-S-01	France	Hautes-Alpes - Col du Galibier	N45.0602°, E6.40344°	9/23/2012	CL
FR01-086-S-01	France	Hautes-Alpes - Col du Galibier	N45.0602°, E6.40344°	9/23/2012	CL
FR01-090-S-01	France	Hautes-Alpes - Col du Galibier	N45.0604°, E6.40303°	9/23/2012	CL
FR01-098-S-01	France	Hautes-Alpes - Col du Galibier	N45.0604°, E6.40303°	9/23/2012	CL
FR01-100-S-01	France	Hautes-Alpes - Col du Galibier	N45.0604°, E6.40285°	9/23/2012	CL
FR01-104-S-01	France	Hautes-Alpes - Col du Galibier	N45.0604°, E6.40285°	nan	CL
FR01-105-S-01	France	Hautes-Alpes - Col du Galibier	N45.0604°, E6.40285°	9/23/2012	CL
FR01-111-S-01	France	Hautes-Alpes - Col du Galibier	N45.0604°, E6.40285°	nan	CL
FR01-115-S-01	France	Hautes-Alpes - Col du Galibier	N45.0607°, E6.40171°	9/23/2012	CL
FR01-116-S-01	France	Hautes-Alpes - Col du Galibier	N45.0605°, E6.40154°	9/22/2012	CL
FR01-118-S-01	France	Hautes-Alpes - Col du Galibier	N45.0606°, E6.40130°	9/22/2012	CL
FR01-119-S-01	France	Hautes-Alpes - Col du Galibier	N45.0605°, E6.40128°	nan	CL
FR01-120-S-01	France	Hautes-Alpes - Col du Galibier	N45.0605°, E6.40112°	9/22/2012	CL
FR01-122-S-01	France	Hautes-Alpes - Col du Galibier	N45.0607°, E6.40104°	9/22/2012	CL
FR01-124-S-01	France	Hautes-Alpes - Col du Galibier	N45.0609°, E6.40074°	9/22/2012	CL
FR01-130-S-01	France	Hautes-Alpes - Col du Galibier	N45.0609°, E6.40222°	nan	CL

FR01-131-S-01	France	Hautes-Alpes - Col du Galibier	N45.0609°, E6.40222°	9/22/2012	CL
FR01-133-S-01	France	Hautes-Alpes - Col du Galibier	N45.0609°, E6.40222°	9/22/2012	CL
FR01-135-S-01	France	Hautes-Alpes - Col du Galibier	N45.0609°, E6.40222°	nan	CL
FR01-137-S-01	France	Hautes-Alpes - Col du Galibier	N45.0609°, E6.40222°	9/22/2012	CL
FR01-139-S-01	France	Hautes-Alpes - Col du Galibier	N45.0609°, E6.40222°	9/22/2012	CL
FR01-400-S-01	France	Hautes-Alpes - Col du Galibier	N45.0606°, E6.40083°	9/22/2012	CL
FR01-404-S-01	France	Hautes-Alpes - Col du Galibier	N45.0611°, E6.40030°	10/11/2014	CL
FR01-407-S-01	France	Hautes-Alpes - Col du Galibier	N45.0616°, E6.40013°	9/22/2012	CL
FR01-409-S-01	France	Hautes-Alpes - Col du Galibier	N45.0619°, E6.39974°	9/22/2012	CL
FR02-015-S-01	France	Hautes-Alpes - Col du Granon	N44.9663°, E6.58244°	9/19/2014	CL
FR02-016-S-01	France	Hautes-Alpes - Col du Granon	N44.9660°, E6.58242°	9/19/2012	CL
FR02-018-S-01	France	Hautes-Alpes - Col du Granon	N44.9663°, E6.58244°	8/27/2014	CL
FR02-026-S-01	France	Hautes-Alpes - Col du Granon	N44.9663°, E6.58244°	2/20/2014	CL
FR02-029-S-01	France	Hautes-Alpes - Col du Granon	N44.9663°, E6.58244°	2/20/2014	CL
FR02-033-S-00	France	Hautes-Alpes - Col du Granon	N44.9663°, E6.58244°	8/24/2011	CL
FR02-037-S-01	France	Hautes-Alpes - Col du Granon	N44.9663°, E6.58244°	8/27/2014	CL
FR02-049-S-01	France	Hautes-Alpes - Col du Granon	N44.9663°, E6.58244°	9/19/2014	CL
FR02-071-S-01	France	Hautes-Alpes - Col du Granon	N44.9663°, E6.58244°	5/15/2017	CL
FR02-079-S-01	France	Hautes-Alpes - Col du Granon	N44.9663°, E6.58244°	9/25/2014	CL
FR02-099-S-01	France	Hautes-Alpes - Col du Granon	N44.9660°, E6.58242°	7/20/2012	CL
FR03-165-S-01	France	Savoy - Pic Blanc du Galibier	N45.0639°, E6.38459°	8/14/2012	CL
FR03-175-S-01	France	Savoy - Pic Blanc du Galibier	N45.0639°, E6.38459°	9/17/2012	CL
FR03-176-S-01	France	Savoy - Pic Blanc du Galibier	N45.0639°, E6.38459°	9/17/2012	CL
FR03-178-S-01	France	Savoy - Pic Blanc du Galibier	N45.0639°, E6.38459°	9/17/2012	CL
FR03-180-S-01	France	Savoy - Pic Blanc du Galibier	N45.0639°, E6.38459°	9/17/2012	CL
FR03-181-S-01	France	Savoy - Pic Blanc du Galibier	N45.0641°, E6.38475°	9/17/2012	CL
FR03-182-S-01	France	Savoy - Pic Blanc du Galibier	N45.0639°, E6.38507°	9/17/2012	CL
FR03-307-S-01	France	Savoy - Pic Blanc du Galibier	N45.0642°, E6.38467°	9/17/2012	CL
FR03-308-S-01	France	Savoy - Pic Blanc du Galibier	N45.0643°, E6.38463°	9/22/2013	CL
FR03-309-S-01	France	Savoy - Pic Blanc du Galibier	N45.0643°, E6.38463°	9/22/2013	CL
FR03-311-S-01	France	Savoy - Pic Blanc du Galibier	N45.0643°, E6.38463°	9/17/2012	CL
FR03-312-S-01	France	Savoy - Pic Blanc du Galibier	N45.0643°, E6.38463°	9/17/2012	CL
FR03-313-S-01	France	Savoy - Pic Blanc du Galibier	N45.0643°, E6.38463°	9/17/2012	CL
FR03-315-S-01	France	Savoy - Pic Blanc du Galibier	N45.0642°, E6.38438°	9/22/2013	CL
FR03-316-S-01	France	Savoy - Pic Blanc du Galibier	N45.0642°, E6.38438°	9/17/2012	CL
FR03-317-S-01	France	Savoy - Pic Blanc du Galibier	N45.0642°, E6.38438°	9/17/2012	CL
FR03-318-S-01	France	Savoy - Pic Blanc du Galibier	N45.0642°, E6.38438°	9/17/2012	CL
FR03-319-S-01	France	Savoy - Pic Blanc du Galibier	N45.0642°, E6.38438°	8/14/2012	CL
FR03-320-S-01	France	Savoy - Pic Blanc du Galibier	N45.0642°, E6.38438°	9/17/2012	CL
FR03-323-S-01	France	Savoy - Pic Blanc du Galibier	N45.0641°, E6.38408°	8/14/2012	CL
FR03-326-S-01	France	Savoy - Pic Blanc du Galibier	N45.0640°, E6.38442°	9/17/2012	CL
FR03-327-S-01	France	Savoy - Pic Blanc du Galibier	N45.0640°, E6.38442°	8/14/2012	CL
FR03-328-S-01	France	Savoy - Pic Blanc du Galibier	N45.0640°, E6.38439°	9/22/2013	CL
FR04-253-S-01	France	Savoy - Col du Galibier	N45.0618°, E6.39436°	8/15/2012	CL
FR04-254-S-01	France	Savoy - Col du Galibier	N45.0618°, E6.39436°	8/15/2012	CL
FR04-256-S-01	France	Savoy - Col du Galibier	N45.0618°, E6.39436°	9/18/2012	CL
FR04-258-S-01	France	Savoy - Col du Galibier	N45.0618°, E6.39436°	9/18/2012	CL
FR04-259-S-01	France	Savoy - Col du Galibier	N45.0618°, E6.39434°	9/18/2012	CL
FR04-260-S-01	France	Savoy - Col du Galibier	N45.0618°, E6.39434°	9/18/2012	CL
FR04-261-S-01	France	Savoy - Col du Galibier	N45.0623°, E6.39290°	9/21/2013	CL
FR04-263-S-01	France	Savoy - Col du Galibier	N45.0629°, E6.39099°	9/18/2012	CL
FR04-264-S-01	France	Savoy - Col du Galibier	N45.0630°, E6.39098°	9/18/2012	CL
FR04-265-S-01	France	Savoy - Col du Galibier	N45.0630°, E6.39098°	9/18/2012	CL
FR04-267-S-01	France	Savoy - Col du Galibier	N45.0630°, E6.39098°	9/18/2012	CL
FR04-272-S-01	France	Savoy - Col du Galibier	N45.0630°, E6.39098°	9/18/2012	CL
FR04-274-S-01	France	Savoy - Col du Galibier	N45.0641°, E6.38916°	9/21/2013	CL
FR04-302-S-01	France	Savoy - Col du Galibier	N45.0641°, E6.38897°	8/15/2012	CL
FR04-303-S-01	France	Savoy - Col du Galibier	N45.0641°, E6.38894°	8/15/2012	CL
FR04-304-S-01	France	Savoy - Col du Galibier	N45.0641°, E6.38894°	9/21/2013	CL
FR04-305-S-01	France	Savoy - Col du Galibier	N45.0641°, E6.38890°	9/21/2013	CL
FR04-350-S-01	France	Savoy - Col du Galibier	N45.0641°, E6.38890°	9/18/2012	CL
FR04-351-S-01	France	Savoy - Col du Galibier	N45.0639°, E6.38897°	9/18/2012	CL
FR06-004-S-01	France	Savoy - Vallon du Fond	N45.0767°, E6.40833°	8/13/2012	CL
FR06-009-S-01	France	Savoy - Vallon du Fond	N45.0763°, E6.39617°	9/19/2013	CL
FR06-010-S-01	France	Savoy - Vallon du Fond	N45.0751°, E6.39122°	8/13/2012	CL
FR06-011-S-01	France	Savoy - Vallon du Fond	N45.0751°, E6.39122°	9/19/2013	CL
FR06-012-S-01	France	Savoy - Vallon du Fond	N45.0751°, E6.39124°	9/19/2013	CL

FR06-013-S-01	France	Savoy - Vallon du Fond	N45.0751°, E6.39130°	9/19/2013	CL
FR06-014-S-01	France	Savoy - Vallon du Fond	N45.0750°, E6.39126°	9/19/2013	CL
FR06-016-S-01	France	Savoy - Vallon du Fond	N45.0752°, E6.38694°	9/19/2013	CL
FR06-029-S-01	France	Savoy - Vallon du Fond	N45.0730°, E6.38124°	nan	CL
FR06-031-S-01	France	Savoy - Vallon du Fond	N45.0730°, E6.38116°	nan	CL
FR06-037-S-01	France	Savoy - Vallon du Fond	N45.0755°, E6.40015°	9/19/2013	CL
FR07-002-S-01	France	Hautes-Alpes - Col du Granon	N44.9660°, E6.58242°	7/20/2012	CL
FR07-003-S-01	France	Hautes-Alpes - Col du Granon	N44.9660°, E6.58242°	7/20/2012	CL
FR07-007-S-01	France	Hautes-Alpes - Col du Granon	N44.9660°, E6.58242°	7/20/2012	CL
FR07-008-S-01	France	Hautes-Alpes - Col du Granon	N44.9660°, E6.58242°	7/20/2012	CL
FR07-010-S-01	France	Hautes-Alpes - Col du Granon	N44.9661°, E6.58259°	7/20/2012	CL
FR07-012-S-01	France	Hautes-Alpes - Col du Granon	N44.9661°, E6.58259°	7/20/2012	CL
FR07-027-S-01	France	Hautes-Alpes - Col du Granon	N44.9661°, E6.58259°	8/10/2012	CL
FR07-030-S-01	France	Hautes-Alpes - Col du Granon	N44.9662°, E6.58246°	8/10/2012	CL
FR07-032-S-01	France	Hautes-Alpes - Col du Granon	N44.9663°, E6.58251°	8/10/2012	CL
FR07-038-S-01	France	Hautes-Alpes - Col du Granon	N44.9663°, E6.58249°	8/10/2012	CL
FR07-046-S-01	France	Hautes-Alpes - Col du Granon	N44.9664°, E6.58243°	8/10/2012	CL
FR07-047-S-01	France	Hautes-Alpes - Col du Granon	N44.9664°, E6.58243°	8/10/2012	CL
FR07-078-S-01	France	Hautes-Alpes - Col du Granon	N44.9663°, E6.58249°	8/10/2012	CL
FR07-081-S-01	France	Hautes-Alpes - Col du Granon	N44.9664°, E6.58243°	8/10/2012	CL
FR07-083-S-01	France	Hautes-Alpes - Col du Granon	N44.9664°, E6.58243°	8/10/2012	CL
FR07-085-S-01	France	Hautes-Alpes - Col du Granon	N44.9664°, E6.58243°	8/10/2012	CL
FR07-086-S-01	France	Hautes-Alpes - Col du Granon	N44.9664°, E6.58243°	8/10/2012	CL
FR07-089-S-01	France	Hautes-Alpes - Col du Granon	N44.9664°, E6.58243°	8/10/2012	CL
FR07-091-S-01	France	Hautes-Alpes - Col du Granon	N44.9664°, E6.58243°	7/20/2012	CL
FR07-092-S-01	France	Hautes-Alpes - Col du Granon	N44.9664°, E6.58243°	8/10/2012	CL
FR07-093-S-01	France	Hautes-Alpes - Col du Granon	N44.9664°, E6.58243°	7/20/2012	CL
FR07-094-S-01	France	Hautes-Alpes - Col du Granon	N44.9664°, E6.58243°	8/10/2012	CL
FR07-095-S-01	France	Hautes-Alpes - Col du Granon	N44.9664°, E6.58243°	7/31/2013	CL
FR07-097-S-01	France	Hautes-Alpes - Col du Granon	N44.9664°, E6.58243°	7/20/2012	CL
FR07-099-S-01	France	Hautes-Alpes - Col du Granon	N44.9664°, E6.58243°	7/20/2012	CL
FR07-101-S-01	France	Hautes-Alpes - Col du Granon	N44.9664°, E6.58243°	7/20/2012	CL
FR07-102-S-01	France	Hautes-Alpes - Col du Granon	N44.9693°, E6.58112°	7/20/2012	CL
FR07-103-S-01	France	Hautes-Alpes - Col du Granon	N44.9693°, E6.58112°	7/20/2012	CL
FR07-104-S-01	France	Hautes-Alpes - Col du Granon	N44.9689°, E6.58104°	7/20/2012	CL
FR07-105-S-01	France	Hautes-Alpes - Col du Granon	N44.9692°, E6.58104°	7/20/2012	CL
FR07-106-S-01	France	Hautes-Alpes - Col du Granon	N44.9692°, E6.58104°	7/20/2012	CL
FR08-001-S-01	France	Savoy - Col de la Croix de Fer	N45.2239°, E6.19116°	9/20/2012	CL
FR08-002-S-01	France	Savoy - Col de la Croix de Fer	N45.2174°, E6.18842°	9/20/2012	CL
FR08-003-S-01	France	Savoy - Col de la Croix de Fer	N45.2174°, E6.18842°	8/16/2012	CL
FR08-004-S-01	France	Savoy - Col de la Croix de Fer	N45.2174°, E6.18842°	8/16/2012	CL
FR08-005-S-01	France	Savoy - Col de la Croix de Fer	N45.2174°, E6.18842°	8/16/2012	CL
FR08-006-S-01	France	Savoy - Col de la Croix de Fer	N45.2174°, E6.18842°	9/20/2012	CL
FR08-008-S-01	France	Savoy - Col de la Croix de Fer	N45.2174°, E6.18842°	9/20/2012	CL
FR08-010-S-01	France	Savoy - Col de la Croix de Fer	N45.2174°, E6.18842°	8/16/2012	CL
FR08-011-S-01	France	Savoy - Col de la Croix de Fer	N45.2137°, E6.18544°	9/20/2012	CL
FR08-012-S-01	France	Savoy - Col de la Croix de Fer	N45.2137°, E6.18544°	9/20/2012	CL
FR08-016-S-01	France	Savoy - Col de la Croix de Fer	N45.2137°, E6.18544°	9/20/2012	CL
FR08-020-S-01	France	Savoy - Col de la Croix de Fer	N45.2137°, E6.18544°	9/20/2012	CL
FR08-022-S-01	France	Savoy - Col de la Croix de Fer	N45.2147°, E6.18452°	9/20/2012	CL
FR08-024-S-01	France	Savoy - Col de la Croix de Fer	N45.2147°, E6.18452°	9/20/2012	CL
FR08-027-S-01	France	Savoy - Col de la Croix de Fer	N45.2143°, E6.18429°	9/20/2012	CL
FR08-030-S-01	France	Savoy - Col de la Croix de Fer	N45.2141°, E6.18404°	9/20/2012	CL
FR08-032-S-01	France	Savoy - Col de la Croix de Fer	N45.2141°, E6.18404°	9/20/2012	CL
FR08-036-S-01	France	Savoy - Col de la Croix de Fer	N45.2111°, E6.18240°	9/20/2012	CL
FR08-037-S-01	France	Savoy - Col de la Croix de Fer	N45.2111°, E6.18240°	9/20/2012	CL
FR08-038-S-01	France	Savoy - Col de la Croix de Fer	N45.2111°, E6.18240°	8/16/2012	CL
FR08-039-S-01	France	Savoy - Col de la Croix de Fer	N45.2111°, E6.18240°	9/20/2012	CL
FR08-040-S-01	France	Savoy - Col de la Croix de Fer	N45.2111°, E6.18208°	9/20/2012	CL
FR08-041-S-01	France	Savoy - Col de la Croix de Fer	N45.2111°, E6.18208°	9/21/2012	CL
FR08-042-S-01	France	Savoy - Col de la Croix de Fer	N45.2111°, E6.18208°	9/21/2012	CL
FR08-044-S-01	France	Savoy - Col de la Croix de Fer	N45.1992°, E6.17089°	9/21/2012	CL
FR08-045-S-01	France	Savoy - Col de la Croix de Fer	N45.1990°, E6.17082°	9/21/2012	CL
FR08-047-S-01	France	Savoy - Col de la Croix de Fer	N45.1980°, E6.16993°	9/21/2012	CL
FR08-049-S-01	France	Savoy - Col de la Croix de Fer	N45.1980°, E6.16988°	9/21/2012	CL
FR08-050-S-01	France	Savoy - Col de la Croix de Fer	N45.1980°, E6.16988°	9/21/2012	CL
FR08-051-S-01	France	Savoy - Col de la Croix de Fer	N45.1958°, E6.17311°	9/21/2012	CL

FR08-052-S-01	France	Savoy - Col de la Croix de Fer	N45.1958°, E6.17311°	9/21/2012	CL
FR08-054-S-01	France	Savoy - Col de la Croix de Fer	N45.1958°, E6.17311°	9/21/2012	CL
FR08-055-S-01	France	Savoy - Col de la Croix de Fer	N45.1957°, E6.17295°	9/24/2013	CL
FR08-060-S-01	France	Savoy - Col de la Croix de Fer	N45.1957°, E6.17295°	9/21/2012	CL
FR08-061-S-01	France	Savoy - Col de la Croix de Fer	N45.1714°, E6.16838°	9/24/2013	CL
FR10-001-S-01	France	Hautes-Alpes - Briançon	N44.9004°, E6.6430°	3/7/2010	CL
FR10-003-S-01	France	Hautes-Alpes - Briançon	N44.9004°, E6.6430°	3/7/2010	CL
FR10-004-S-01	France	Hautes-Alpes - Briançon	N44.9004°, E6.6430°	3/7/2010	CL
FR10-005-S-01	France	Hautes-Alpes - Briançon	N44.9004°, E6.6430°	3/7/2010	CL
FR15-325-S-01	France	Savoy - La Valloirette	N45.0882°, E6.43233°	8/13/2012	CL
FR15-326-S-01	France	Savoy - La Valloirette	N45.0882°, E6.43233°	8/13/2012	CL
FR15-327-S-01	France	Savoy - La Valloirette	N45.0882°, E6.43233°	8/13/2012	CL
FR15-328-S-01	France	Savoy - La Valloirette	N45.0882°, E6.43233°	9/19/2013	CL
FR15-329-S-01	France	Savoy - La Valloirette	N45.0882°, E6.43233°	9/19/2013	CL
FR15-330-S-01	France	Savoy - La Valloirette	N45.0811°, E6.42592°	8/13/2012	CL
FR15-331-S-01	France	Savoy - La Valloirette	N45.0811°, E6.42584°	8/13/2012	CL
FR15-332-S-01	France	Savoy - La Valloirette	N45.0814°, E6.42587°	8/13/2012	CL
FR16-001-C-00	France	Hautes-Alpes - Col de la Trancoulette	N44.8917°, E6.55038°	7/11/2021	CL
FR16-002-C-00	France	Hautes-Alpes - Col de la Trancoulette	N44.8917°, E6.55038°	7/11/2021	CL
FR16-004-C-00	France	Hautes-Alpes - Col de la Trancoulette	N44.8917°, E6.55038°	7/11/2021	CL
FR17-001-C-00	France	Hautes-Alpes - Col de la Pisse	N44.9038°, E6.52796°	7/12/2021	CL
FR17-002-C-00	France	Hautes-Alpes - Col de la Pisse	N44.9038°, E6.52796°	7/12/2021	CL
FR17-003-C-00	France	Hautes-Alpes - Col de la Pisse	N44.9038°, E6.52796°	7/12/2021	CL
FR17-004-C-00	France	Hautes-Alpes - Col de la Pisse	N44.9038°, E6.52796°	7/12/2021	CL
FR18-005-C-00	France	Hautes-Alpes - Col des Marseilles	N44.8322°, E6.81208°	7/14/2021	CL
FR19-001-C-00	France	Hautes-Alpes - Cime de la Condamine	N44.8933°, E6.51945°	7/20/2021	CL
FR19-002-C-00	France	Hautes-Alpes - Cime de la Condamine	N44.8933°, E6.51945°	7/20/2021	CL
FR19-003-C-00	France	Hautes-Alpes - Cime de la Condamine	N44.8933°, E6.51945°	7/20/2021	CL
FR20-001-C-00	France	Hautes-Alpes - Les Combes	N44.8944°, E6.56460°	7/21/2021	CL
FR20-004-C-00	France	Hautes-Alpes - Les Combes	N44.8944°, E6.56460°	7/21/2021	CL
FR20-005-C-00	France	Hautes-Alpes - Les Combes	N44.8944°, E6.56460°	7/21/2021	CL
FR21-001-C-00	France	Hautes-Alpes - Col d'Arsine	N44.9749°, E6.41017°	7/22/2021	CL
FR21-002-C-00	France	Hautes-Alpes - Col d'Arsine	N44.9749°, E6.41017°	7/22/2021	CL
FR21-003-C-00	France	Hautes-Alpes - Col d'Arsine	N44.9740°, E6.41544°	7/22/2021	CL
FR21-004-C-00	France	Hautes-Alpes - Col d'Arsine	N44.9740°, E6.41544°	7/22/2021	CL

Table S2: Correlation between pairwise genetic differences and geographic distances, and between pairwise genetic differences and environmental distances using the Mantel test and the Partial Mantel test.

Setup	Distance Matrix	Mantel Test		Partial Mantel Test		
		r	Significance	Conditioning Distance	r	Significance
IBD	geographic distance	0.786	< 0.0001	environmental distance	0.754	< 0.0001
IBE	environmental distance	0.485	< 0.0001	geographic distance	0.366	< 0.0001

Table S3: Split times among population pairs (measured in thousands of years ago, and including all populations from Spain, and FR01 as a representative from the Alps), on the basis of the rCCR analysis with Relate.

Pop	ES01	ES02	ES03	ES04	ES05	ES06	ES08	ES09	ES10	ES17	ES23	ES24	ES25
**ES01**	0	9.2 (7.4 - 12.4)	19.7 (17.2 - 25.2)	17.8 (15.5 - 23.3)	32 (27.1 - 37.9)	6.4 (5.4 - 7.5)	130.6 (109.3 - 136.5)	109 (87.3 - 115.7)	27.5 (21.4 - 36.4)	64.7 (47.4 - 85.2)	37.9 (25.7 - 46.6)	107.4 (89.9 - 114.3)	131.7 (113.3 - 135.3)
**ES02**	9.2 (7.4 - 12.4)	0	13.1 (1.2 - 17)	18.1 (13.2 - 28.3)	29.3 (22.5 - 34.1)	10.4 (7.7 - 13.9)	132.9 (113.3 - 140.2)	109 (86.5 - 119.8)	25.9 (17.9 - 37.7)	48.2 (32.9 - 72)	43 (31.2 - 53.7)	98.2 (73.3 - 111.3)	134.1 (116.3 - 137.7)
**ES03**	19.7 (17.2 - 25.2)	13.1 (1.2 - 17)	0	19.1 (15.6 - 24.1)	21.9 (16.6 - 26.4)	19.1 (15.5 - 24.5)	137.7 (117.4 - 145.3)	119.8 (100.1 - 126.1)	22 (15.2 - 26.8)	78 (53.7 - 90.7)	41.1 (29.8 - 49.1)	115.3 (100 - 122.7)	142.7 (124.9 - 146.6)
**ES04**	17.8 (15.5 - 23.3)	18.1 (13.2 - 28.3)	19.1 (15.6 - 24.1)	0	9.6 (7.3 - 11.5)	19.3 (14.9 - 23.7)	136.5 (116.3 - 145.3)	113.8 (92.6 - 122.9)	25 (18.1 - 33.4)	70.1 (43.4 - 82.2)	15.7 (11.6 - 21.3)	113.3 (91.5 - 124.9)	140.2 (120.5 - 147.9)
**ES05**	32 (27.1 - 37.9)	29.3 (22.5 - 34.1)	21.9 (16.6 - 26.4)	9.6 (7.3 - 11.5)	0	33.5 (27.1 - 38.3)	135.3 (113.3 - 142.7)	110.9 (89.5 - 119.8)	22 (17.3 - 26.5)	68.8 (43 - 80.1)	20.2 (15.7 - 24.8)	112.3 (93.1 - 122.7)	141.4 (120.5 - 147.9)
**ES06**	6.4 (5.4 - 7.5)	10.4 (7.7 - 13.9)	19.1 (15.5 - 24.5)	19.3 (14.9 - 23.7)	33.5 (27.1 - 38.3)	0	130.6 (108.3 - 137.7)	104.4 (82.9 - 112.8)	34.3 (26.8 - 41.8)	52.3 (41.1 - 67)	40.7 (28.8 - 48.7)	98.2 (74.6 - 109.3)	135.3 (117.4 - 138.9)
**ES08**	130.6 (109.3 - 136.5)	132.9 (113.3 - 140.2)	137.7 (117.4 - 145.3)	136.5 (116.3 - 145.3)	135.3 (113.3 - 142.7)	130.6 (108.3 - 137.7)	0	23.1 (18.8 - 28.2)	144.6 (118.8 - 157.5)	89.1 (73.3 - 95.7)	127.1 (107.4 - 137.7)	117.4 (100 - 126)	140.2 (121.6 - 146.6)
**ES09**	109 (87.3 - 115.7)	109 (86.5 - 119.8)	119.8 (100.1 - 126.1)	113.8 (92.6 - 122.9)	110.9 (89.5 - 119.8)	104.4 (82.9 - 112.8)	23.1 (18.8 - 28.2)	0	126.1 (99.2 - 137.4)	54 (40.7 - 63.6)	102.7 (79.4 - 114.8)	74.2 (60.4 - 90.3)	121.8 (105.3 - 127.2)
**ES10**	27.5 (21.4 - 36.4)	25.9 (17.9 - 37.7)	22 (15.2 - 26.8)	25 (18.1 - 33.4)	22 (17.3 - 26.5)	34.3 (26.8 - 41.8)	144.6 (118.8 - 157.5)	126.1 (99.2 - 137.4)	0	82.9 (53.1 - 100.9)	42.1 (30.2 - 48.7)	127.2 (99.2 - 140.9)	149.6 (123.9 - 157.5)
**ES17**	64.7 (47.4 - 85.2)	48.2 (32.9 - 72)	78 (53.7 - 90.7)	70.1 (43.4 - 82.2)	68.8 (43 - 80.1)	52.3 (41.1 - 67)	89.1 (73.3 - 95.7)	54 (40.7 - 63.6)	82.9 (53.1 - 100.9)	0	67 (50.4 - 79.4)	61.9 (48.2 - 78.7)	120.5 (103.6 - 128.3)
**ES23**	37.9 (25.7 - 46.6)	43 (31.2 - 53.7)	41.1 (29.8 - 49.1)	15.7 (11.6 - 21.3)	20.2 (15.7 - 24.8)	40.7 (28.8 - 48.7)	127.1 (107.4 - 137.7)	102.7 (79.4 - 114.8)	42.1 (30.2 - 48.7)	67 (50.4 - 79.4)	0	94 (62.4 - 108.3)	132.9 (116.3 - 138.9)
**ES24**	107.4 (89.9 - 114.3)	98.2 (73.3 - 111.3)	115.3 (100 - 122.7)	113.3 (91.5 - 124.9)	112.3 (93.1 - 122.7)	98.2 (74.6 - 109.3)	117.4 (100 - 126)	74.2 (60.4 - 90.3)	127.2 (99.2 - 140.9)	61.9 (48.2 - 78.7)	94 (62.4 - 108.3)	0	50 (41.5 - 56.6)
**ES25**	131.7 (113.3 - 135.3)	134.1 (116.3 - 137.7)	142.7 (124.9 - 146.6)	140.2 (120.5 - 147.9)	141.4 (120.5 - 147.9)	135.3 (117.4 - 138.9)	140.2 (121.6 - 146.6)	121.8 (105.3 - 127.2)	149.6 (123.9 - 157.5)	120.5 (103.6 - 128.3)	132.9 (116.3 - 138.9)	50 (41.5 - 56.6)	0
**FR01**	228.6 (205.5 - 243.3)	226.6 (1.9 - 239)	230.6 (211 - 245.4)	228.6 (209.2 - 243.3)	226.6 (209.2 - 241.1)	228.6 (209.2 - 241.1)	211 (193.1 - 224.6)	208.9 (193.5 - 218.1)	227.6 (208.9 - 248)	214.8 (196.5 - 226.6)	220.6 (203.6 - 236.9)	198.3 (186.3 - 212.9)	200.1 (188 - 214.8)

Table S4: Population site coordinates and monthly average temperature obtained from https://www.worldclim.org/ for all Spanish and French populations, and for other European populations previously studied by Wunder et al. 2023, which are denoted by an asterisk (*).

Population	Location		Monthly average temperature (°C)											
**ID**	**Country**	**Coordinates**	**Jan**	**Feb**	**Mar**	**Apr**	**May**	**Jun**	**Jul**	**Aug**	**Sep**	**Oct**	**Nov**	**Dec**
ES01	Spain	N42.9931°, W5.91017°	-0.7	-0.2	2.1	3.5	7.1	11.7	15.1	15.1	12.1	7.1	2.8	0.5
ES02	Spain	N42.9925°, W5.75975°	1.2	2.0	4.3	5.6	9.1	13.4	16.4	16.3	13.7	9.0	4.7	2.3
ES03	Spain	N43.2298°, W5.93906°	1.1	1.7	3.7	4.8	8.2	12.4	15.4	15.4	12.9	8.3	4.3	2.2
ES04	Spain	N43.0514°, W6.10427°	-0.8	-0.3	2.1	3.6	7.2	11.7	15.0	15.0	12.1	7.2	2.8	0.4
ES05	Spain	N43.0780°, W6.23478°	2.6	3.3	5.6	6.9	10.1	14.1	16.9	16.9	14.5	10.1	5.9	3.6
ES06	Spain	N43.0007°, W5.92695°	-1.3	-0.8	1.5	3.0	6.6	11.2	14.6	14.7	11.7	6.6	2.3	0.0
ES08	Spain	N43.2072°, W4.81495°	-2.7	-2.2	-0.1	1.2	4.9	9.4	12.8	12.7	9.9	5.1	1.0	-1.3
ES09	Spain	N43.1491°, W4.80754°	1.1	2.0	4.2	5.6	9.0	13.0	16.0	15.9	13.4	8.9	4.8	2.3
ES10	Spain	N43.1238°, W6.05863°	4.0	4.8	7.1	8.4	11.5	15.1	17.6	17.7	15.4	11.4	7.3	4.9
ES15	Spain	N43.1427°, W5.57926°	5.4	6.5	8.6	9.7	12.7	16.1	18.5	18.6	16.5	12.6	8.7	6.3
ES17	Spain	N43.0127°, W5.27930°	1.4	2.5	5.1	6.6	10.1	14.2	17.2	17.1	14.3	9.6	5.3	2.7
ES23	Spain	N43.0016°, W6.42420°	-0.3	0.1	2.4	3.9	7.3	11.8	14.9	14.9	12.2	7.5	3.2	0.9
ES24	Spain	N43.0398°, W4.36312°	-0.5	0.1	2.1	3.6	7.3	11.5	14.7	14.7	12.0	7.3	3.1	0.6
ES25	Spain	N43.1532°, W3.56994°	2.7	3.4	5.0	6.2	9.7	13.2	16.0	16.2	13.9	9.8	5.8	3.7
FR01a	France	N45.0641°, E6.38386°	-7.8	-7.9	-6.9	-5.0	0.0	3.9	7.8	8.7	5.3	1.1	-3.7	-6.3
FR01b	France	N45.0605°, E6.40357°	-7.4	-7.4	-6.2	-4.2	0.7	4.6	8.5	9.3	5.9	1.6	-3.2	-5.9
FR02	France	N44.9663°, E6.58244°	-4.7	-4.5	-2.9	-0.7	4.3	8.0	11.6	12.1	8.6	4.2	-0.8	-3.4
FR03	France	N45.0188°, E5.56947°	-2.9	-2.6	-1.0	1.0	5.9	9.4	12.8	13.0	9.5	5.3	0.6	-1.8
FR04	France	N45.0259°, E5.57166°	-2.5	-2.1	-0.2	1.9	6.8	10.2	13.6	13.7	10.2	6.0	1.2	-1.3
FR06	France	N45.0756°, E6.38655°	-6.8	-6.8	-5.4	-3.3	1.6	5.5	9.2	10.0	6.6	2.3	-2.6	-5.3
FR07	France	N44.9647°, E6.61034°	-5.5	-5.5	-4.3	-2.3	2.7	6.6	10.3	10.9	7.4	3.1	-1.7	-4.2
FR08	France	N45.1994°, E6.17098°	-7.4	-7.4	-6.1	-4.2	0.7	4.6	8.4	9.2	5.9	1.7	-3.2	-6.0
FR10	France	N44.9004°, E6.64300°	-1.5	-0.8	1.8	4.4	9.2	12.7	15.9	15.9	12.2	7.5	2.3	-0.5
FR15	France	N45.0272°, E6.55434°	-4.5	-4.1	-2.1	0.3	5.2	9.0	12.6	12.9	9.4	4.9	-0.5	-3.2
FR16	France	N44.8917°, E6.55038°	-4.9	-4.6	-2.9	-0.7	4.3	8.0	11.6	12.0	8.5	4.1	-0.9	-3.6
FR18	France	N44.8337°, E6.80845°	-5.9	-5.7	-4.0	-1.8	3.2	7.1	10.6	11.1	7.7	3.3	-1.7	-4.5
FR19	France	N44.8934°, E6.51945°	-6.9	-7.1	-6.1	-4.3	0.8	4.7	8.5	9.3	5.8	1.5	-3.2	-5.6
FR20	France	N44.8945°, E6.56460°	-4.6	-4.3	-2.5	-0.3	4.8	8.5	11.9	12.4	8.9	4.5	-0.6	-3.4
FR21	France	N44.9749°, E6.41017°	-7.1	-7.0	-5.6	-3.6	1.2	5.1	8.8	9.5	6.1	1.8	-3.0	-5.7
S1*	Sweden	N68.4136°, E18.31968°	-11.1	-11.1	-9.1	-5.1	0.6	6.3	9.7	7.9	3.2	-2.0	-6.9	-9.6
S2*	Sweden	N68.3608°, E18.71694°	-11.7	-11.4	-8.9	-4.6	1.4	7.0	10.0	8.3	3.6	-1.9	-7.3	-10.0
S3*	Sweden	N63.8541°, E13.94924°	-8.7	-8.4	-5.9	-2.6	3.4	8.2	10.4	9.0	4.6	0.1	-5.0	-7.5
S4*	Sweden	N62.8344°, E11.74453°	-9.4	-8.9	-6.2	-2.3	3.2	8.3	10.8	9.5	5.2	0.5	-5.2	-8.1
S5*	Sweden	N63.2082°, E12.34990°	-9.2	-9.0	-7.1	-3.7	1.9	6.7	9.3	8.1	3.8	-0.7	-5.7	-7.9
N1*	Norway	N70.0000°, E26.00000°	-12.5	-12.1	-9.5	-5.6	-0.4	5.7	9.7	7.9	2.9	-3.0	-8.9	-11.2
IS1*	Iceland	N65.0000°, W18.00000°	-4.7	-5.4	-5.6	-3.7	0.4	4.8	7.6	6.8	2.2	-1.3	-3.6	-4.8
IS2*	Iceland	N64.1000°, W18.37000°	-3.1	-3.6	-3.5	-1.2	2.7	6.5	8.7	8.0	4.6	1.0	-1.6	-3.1
CH1*	Switzerland	N46.6500°, E7.88300°	-0.1	1.0	4.4	7.6	12.2	14.9	17.1	16.8	13.5	9.0	3.6	0.6
CH2*	Switzerland	N47.3560°, E7.77200°	-0.3	0.4	3.4	6.2	10.8	13.8	16.2	15.9	12.5	8.2	3.2	0.6
CH3*	Switzerland	N45.9836°, E7.78424°	-9.7	-9.9	-9.2	-7.5	-2.7	1.0	4.6	5.5	2.5	-1.2	-5.7	-8.0

Table S5: Population site coordinates and monthly average precipitation obtained from https://www.worldclim.org/ for all Spanish and French populations, and for other European populations previously studied by Wunder et al. 2023, which are denoted by an asterisk (*).

**Population**	**Location**		**Monthly average precipitation (mm)**											
**ID**	**Country**	**Coordinates**	**Jan**	**Feb**	**Mar**	**Apr**	**May**	**Jun**	**Jul**	**Aug**	**Sep**	**Oct**	**Nov**	**Dec**
ES01	Spain	N42.9931°, W5.91017°	146	120	104	121	121	77	60	53	77	127	146	154
ES02	Spain	N42.9925°, W5.75975°	114	95	79	98	104	66	53	47	65	103	116	122
ES03	Spain	N43.2298°, W5.93906°	138	116	99	123	123	77	65	59	79	122	139	146
ES04	Spain	N43.0514°, W6.10427°	149	123	106	127	123	77	59	55	79	130	150	158
ES05	Spain	N43.0780°, W6.23478°	121	102	88	107	102	63	49	48	70	119	132	132
ES06	Spain	N43.0007°, W5.92695°	153	127	109	130	125	79	62	55	80	132	152	161
ES08	Spain	N43.2072°, W4.81495°	152	121	98	140	143	83	67	59	79	124	148	161
ES09	Spain	N43.1491°, W4.80754°	102	84	71	97	103	62	55	47	61	93	102	109
ES10	Spain	N43.1238°, W6.05863°	103	90	79	100	92	57	47	44	60	105	113	112
ES15	Spain	N43.1427°, W5.57926°	83	76	67	90	85	51	46	42	51	84	87	88
ES17	Spain	N43.0127°, W5.27930°	92	76	61	82	87	53	45	39	53	83	91	99
ES23	Spain	N43.0016°, W6.42420°	159	132	113	131	122	78	56	54	82	141	160	169
ES24	Spain	N43.0398°, W4.36312°	118	94	78	109	116	69	56	50	66	100	116	127
ES25	Spain	N43.1532°, W3.56994°	106	88	74	112	109	68	57	57	67	95	112	113
FR01a	France	N45.0641°, E6.38386°	142	124	120	117	123	94	70	86	98	133	156	143
FR01b	France	N45.0605°, E6.40357°	143	125	122	121	126	98	72	88	101	137	158	146
FR02	France	N44.9663°, E6.58244°	131	116	109	119	120	96	70	86	98	135	152	138
FR03	France	N45.0188°, E5.56947°	138	117	118	127	141	113	78	97	121	146	167	146
FR04	France	N45.0259°, E5.57166°	129	109	111	118	133	111	76	95	119	140	160	139
FR06	France	N45.0756°, E6.38655°	144	128	123	123	133	104	78	94	105	141	163	149
FR07	France	N44.9647°, E6.61034°	133	117	111	121	119	93	69	84	96	134	152	138
FR08	France	N45.1994°, E6.17098°	152	132	126	124	134	109	79	94	109	145	170	157
FR10	France	N44.9004°, E6.64300°	100	91	81	86	96	86	61	74	87	119	117	110
FR15	France	N45.0272°, E6.55434°	126	114	108	116	116	98	72	86	98	134	148	136
FR16	France	N44.8917°, E6.55038°	129	114	111	117	121	95	68	85	98	135	148	136
FR18	France	N44.8337°, E6.80845°	122	107	104	115	118	89	63	79	92	129	137	125
FR19	France	N44.8934°, E6.51945°	133	113	111	113	117	88	63	80	93	128	145	133
FR20	France	N44.8945°, E6.56460°	127	113	111	117	118	95	68	84	98	135	148	134
FR21	France	N44.9749°, E6.41017°	140	121	115	116	124	94	68	84	98	135	155	144
S1*	Sweden	N68.4136°, E18.31968°	72	59	44	37	42	53	82	83	67	79	64	71
S2*	Sweden	N68.3608°, E18.71694°	50	41	34	28	37	48	76	68	48	56	46	49
S3*	Sweden	N63.8541°, E13.94924°	48	55	51	52	41	94	98	74	108	101	60	34
S4*	Sweden	N62.8344°, E11.74453°	76	63	56	53	53	81	106	97	95	76	71	75
S5*	Sweden	N63.2082°, E12.34990°	73	63	59	58	55	91	111	98	109	92	75	74
N1*	Norway	N70.0000°, E26.00000°	36	30	32	35	37	61	71	63	56	48	40	36
IS1*	Iceland	N65.0000°, W18.00000°	96	92	93	64	61	79	80	102	104	118	92	95
IS2*	Iceland	N64.1000°, W18.37000°	133	135	126	97	100	105	108	141	130	154	128	138
CH1*	Switzerland	N46.6500°, E7.88300°	75	81	76	83	106	130	121	124	83	88	90	84
CH2*	Switzerland	N47.3560°, E7.77200°	108	108	99	110	128	145	130	122	103	114	124	128
CH3*	Switzerland	N45.9836°, E7.78424°	48	44	51	44	46	42	40	47	43	44	51	43

Table S6: Population site coordinates and monthly Airidtiy Index (AI) obtained from Zomer et al. 2022 for all Spanish and French populations, and for other European populations previously studied by Wunder et al. 2023, which are denoted by an asterisk (*).

**Population**	**Location**		**Monthly Aridity Index** (×10000)											
**ID**	**Country**	**Coordinates**	**Jan**	**Feb**	**Mar**	**Apr**	**May**	**Jun**	**Jul**	**Aug**	**Sep**	**Oct**	**Nov**	**Dec**
ES01	Spain	N42.9931°, W5.91017°	39069	31351	20604	16783	11721	5489	3266	3038	6415	19301	38334	44974
ES02	Spain	N42.9925°, W5.75975°	34540	24255	12191	11643	9507	4714	2965	2832	5631	15259	28315	40266
ES03	Spain	N43.2298°, W5.93906°	34983	26143	16637	16843	11989	6017	3990	3809	6702	17530	31947	42497
ES04	Spain	N43.0514°, W6.10427°	43059	37654	22272	18860	12234	5579	3281	3259	6807	19467	39459	58485
ES05	Spain	N43.0780°, W6.23478°	37617	24889	12616	12590	9358	4571	2861	3018	6239	17486	33309	45042
ES06	Spain	N43.0007°, W5.92695°	48476	35678	22999	19296	12494	5627	3410	3166	6719	21057	42618	56296
ES08	Spain	N43.2072°, W4.81495°	41976	35087	21243	26830	16299	6640	4040	3805	7421	20657	41852	61019
ES09	Spain	N43.1491°, W4.80754°	37928	21132	13128	12944	9636	4776	3392	3094	5471	15778	31549	42830
ES10	Spain	N43.1238°, W6.05863°	31085	22061	11073	11257	8124	4254	2945	2952	5620	15411	29481	38093
ES15	Spain	N43.1427°, W5.57926°	22676	16157	8673	9548	7272	3841	2964	2921	4895	12070	20371	26536
ES17	Spain	N43.0127°, W5.27930°	28943	19308	8739	9227	7189	3589	2495	2361	4471	11809	22655	33464
ES23	Spain	N43.0016°, W6.42420°	42761	37921	21949	19039	11901	5645	3138	3204	6987	21356	40455	51877
ES24	Spain	N43.0398°, W4.36312°	32331	22817	15538	15606	11231	5042	3254	3116	5997	14712	27953	41520
ES25	Spain	N43.1532°, W3.56994°	24526	18320	11062	14302	9913	5428	3638	3970	6002	13621	24954	29428
FR01a	France	N45.0641°, E6.38386°	43921	38557	29264	22405	16175	9349	5325	6556	10639	20708	36837	43110
FR01b	France	N45.0605°, E6.40357°	46638	39911	29091	22561	16308	9579	5309	6588	10954	21658	35694	46150
FR02	France	N44.9663°, E6.58244°	48610	30357	20046	17426	12186	7825	4536	5915	9394	18972	34917	41240
FR03	France	N45.0188°, E5.56947°	36040	29607	20274	18684	14027	9528	5224	6738	12148	21347	38508	38145
FR04	France	N45.0259°, E5.57166°	39932	26896	18329	16584	13119	9066	4956	6592	12111	20759	38195	37528
FR06	France	N45.0756°, E6.38655°	54006	41196	27198	22214	16537	9952	5874	7045	11148	21927	39543	46207
FR07	France	N44.9647°, E6.61034°	48752	33299	20967	19488	13146	7873	4667	5908	9392	19824	34164	35640
FR08	France	N45.1994°, E6.17098°	64655	40412	30390	23459	18137	11378	6143	7285	11970	23272	40727	45541
FR10	France	N44.9004°, E6.64300°	26556	20841	11789	9981	7995	6078	3730	4850	7854	16794	28149	31021
FR15	France	N45.0272°, E6.55434°	48672	30052	20481	17524	12118	7985	4657	5982	9567	20205	38294	44825
FR16	France	N44.8917°, E6.55038°	50442	31755	21307	17800	12702	7814	4484	5971	9784	19913	33881	44013
FR18	France	N44.8337°, E6.80845°	53471	32206	21053	18500	12896	7559	4291	5744	9610	19954	31885	45895
FR19	France	N44.8934°, E6.51945°	50081	35356	24829	20276	14244	8172	4482	5872	9758	19568	31307	38029
FR20	France	N44.8945°, E6.56460°	44827	32353	20358	17669	11972	7735	4431	5864	9628	20150	36248	39196
FR21	France	N44.9749°, E6.41017°	65535	41716	25962	20557	15868	8915	5191	6426	10095	21127	38305	47218
S1*	Sweden	N68.4136°, E18.31968°	65535	61744	24642	10874	7577	6045	9006	13653	21350	41844	54955	65535
S2*	Sweden	N68.3608°, E18.71694°	65535	65535	21042	7540	5783	5254	7763	10408	13264	31445	34052	65535
S3*	Sweden	N63.8541°, E13.94924°	45984	42851	18201	13512	5310	9593	10659	11502	30766	54492	65535	51793
S4*	Sweden	N62.8344°, E11.74453°	65535	54838	23518	14853	7100	8405	10145	12637	22165	34409	65535	65535
S5*	Sweden	N63.2082°, E12.34990°	65535	48471	26635	17329	8450	10452	11853	15242	33643	44275	65535	59071
N1*	Norway	N70.0000°, E26.00000°	65535	65535	23837	11166	6875	7417	7632	10641	21166	40755	65535	65535
IS1*	Iceland	N65.0000°, W18.00000°	31207	42509	41963	18100	11500	11449	9209	15516	40368	32150	33402	34834
IS2*	Iceland	N64.1000°, W18.37000°	61807	65535	60162	26885	17156	14528	13731	21632	33460	54871	48815	65469
CH1*	Switzerland	N46.6500°, E7.88300°	30167	22288	11536	9501	9190	10051	8289	10081	9769	16277	27905	42804
CH2*	Switzerland	N47.3560°, E7.77200°	44384	33119	18103	14144	11987	12059	9638	10494	12615	22742	41687	54229
CH3*	Switzerland	N45.9836°, E7.78424°	24323	14805	14095	9960	7368	4902	3789	4469	5883	7703	12698	16507

Table S7: Drift rates scaled by $2 \times Ne$ calculated based on F3 statistics. Populations A and B are focus populations and population C is the outgroup population (F = French populations, CE-CAN = Central-Eastern Cantabrian populations, and E-CAN = Eastern Cantabrian populations). Drift rates from the ancestor of population A and B to population A (drift rate A), to population B (drift rate B) and to population C (drift rate C; through the ancestor of population A, B and C). Sets denote combinations of populations in the analysis.

**Set**	**Population A**	**Population B**	**Population C**	**Drift rate A**	**Drift rate B**	**Drift rate C**
Set1	W-CAN2	W-CAN1	F	0.177	0.166	3.353
Set2	W-CAN2	ES17	F	0.653	0.319	2.778
Set3	W-CAN1	ES17	F	0.638	0.301	0.285
Set4	W-CAN2	W-CAN1	CE-CAN	0.082	0.089	0.414
Set5	W-CAN2	ES17	E-CAN	0.223	0.264	0.408
Set6	W-CAN1	ES17	E-CAN	0.218	0.250	0.419

Table S8: Raw and standardized CLR-scores from empirical data (observed), and from simulations (simulated), corresponding to the upper 5%, 1% and 0.5% quantiles for all tested branches and sets. For reference, among the observed CLR-score thresholds, the one used for the GO enrichment analyses is the standardized 0.5% threshold. The other observed scores are shown for comparison with the simulations. *A* stands for ancestral branch, *T* for terminal branch. The *sets* correspond to the ones described in Supplementary Table S7.

		**Observed CLR-score**						**Simulated CLR-score**					
		**Raw**			**Standardized**			**Raw**			**Standardized**		
**Set**	**Branch**	**5%**	**1%**	**0.5%**	**5%**	**1%**	**0.5%**	**5%**	**1%**	**0.5%**	**5%**	**1%**	**0.5%**
Set1	T(W-CAN2)	63.36	81.74	89.89	2.22	3.13	3.53	66.72	84.18	90.68	2.12	2.93	3.23
Set1	T(W-CAN1)	77.91	98.15	108.00	2.12	2.92	3.31	75.81	97.02	103.94	2.04	2.75	3.02
Set1	A(W-CAN1, W-CAN2)	606.59	687.22	709.14	1.97	2.40	2.52	589.40	691.99	714.12	2.15	2.75	2.89
Set2	T(W-CAN2)	121.28	150.09	161.22	1.95	2.67	2.95	115.64	138.97	145.75	1.78	2.30	2.47
Set2	T(ES17)	62.79	82.00	91.33	2.01	2.95	3.41	71.24	87.34	93.86	1.84	2.56	2.84
Set2	A(W-CAN2, ES17)	540.55	603.45	620.86	1.68	2.05	2.15	545.75	606.91	622.80	1.81	2.20	2.30
Set3	T(W-CAN1)	132.94	160.09	170.39	1.88	2.50	2.73	124.57	148.28	155.45	1.75	2.23	2.40
Set3	T(ES17)	59.62	76.70	85.97	2.01	2.89	3.37	68.82	84.93	91.77	1.85	2.58	2.90
Set3	A(W-CAN1, ES17)	552.92	613.59	633.98	1.66	2.00	2.12	550.88	614.16	630.16	1.84	2.24	2.33
Set4	T(W-CAN2)	32.78	80.29	102.95	1.57	4.54	5.96	35.40	82.88	103.78	1.67	4.62	5.91
Set4	T(W-CAN1)	46.35	94.86	113.19	1.95	4.56	5.54	45.43	102.09	124.18	1.76	4.65	5.78
Set4	A(W-CAN1, W-CAN2)	219.81	359.78	424.74	1.89	3.60	4.39	155.84	216.98	239.75	2.30	3.47	3.91
Set5	T(W-CAN2)	82.73	132.05	155.46	2.19	3.88	4.69	82.54	122.81	137.93	2.18	3.62	4.16
Set5	T(ES17)	60.28	96.35	113.28	2.08	3.77	4.56	94.08	129.90	143.86	1.97	3.11	3.56
Set5	A(W-CAN2, ES17)	231.56	387.12	466.61	1.83	3.62	4.54	215.21	296.32	335.18	1.79	2.88	3.40
Set6	T(W-CAN1)	95.82	141.49	160.16	2.31	3.73	4.32	88.59	130.82	146.66	2.19	3.60	4.13
Set6	T(ES17)	57.99	93.49	110.43	2.13	3.87	4.70	93.06	129.69	144.31	1.99	3.16	3.63
Set6	A(W-CAN1, ES17)	235.48	411.59	486.22	1.76	3.72	4.55	218.10	301.45	341.38	1.80	2.89	3.43

Table S9: Candidate genes that overlapped the highest *3P-CLR* peaks, analyzed with different outgroups. Gene names, *A. alpina* gene ID, *A. thaliana* orthologs, branches with signatures of positive selection (T = terminal, A = ancestral), maximum normalized CLR score, maximum *XP-EHH* score (NT = not tested; NA = no overlapping phased SNPs; asteriscs denote the upper 0.5% tail), molecular function from UniProt entries (Consortium 2023), and outgroup (F = French populations, CE-CAN = Central-Eastern Cantabrian populations, and E-CAN = Eastern Cantabrian populations).

**Gene name**	***A. alpina* ID**	***A. thaliana* ortholog**	**Branch(es)**	**Normalized CLR**	**XP-EHH**	**Function**	**Outgroup**
SC5D	AA6G27580	At3g02580	T(W-CAN2)	10.53	2.57*	Involved in the biosynthesis of precursor of growth-promoting brassinosteroids	F
HPGT3	AA6G26170	At2g25300	T(ES17)	8.16	NT	Essential for AGP glycosylation	F
NA	AA6G26180	At3g58210	T(ES17)	8.16	NT	May be linked to secondary cell wall biosynthesis	F
NA	AA7G10040	NA	T(W-CAN1)	7.49	1.11*	Unknown function	F
CYP57	AA7G10050	At4g33060	T(W-CAN1)	6.81	1.11*	Functions as a molecular chaperone	F
TWD40-2	AA8G45050	At5g24710	T(W-CAN2)	6.95	0.10	Involved in clathrin mediated endocytosis	F
KAS2	AA2G23850	At5g24710	T(ES17)	6.60	NT	Involved in the regulation of fatty acids ratios and cold response	F
ORC3	AA8G20070	At5g16690	A(W-CAN1, W-CAN2, ES17)	3.19	NT	Involved in DNA replication	F
BIR1 (paralog1)	AA8G20080	At5g48380	A(W-CAN1, W-CAN2, ES17)	3.19	NT	Involved in negative regulation of plant immunity	F
GA20OX4	AA2G06360	At1g60980	A(W-CAN1, W-CAN2, ES17)	3.05	NT	Key oxidase enzyme in the biosynthesis of gibberellin	F
KIB4	AA8G20040	At3g03730	A(W-CAN1, W-CAN2, ES17)	2.97	NT	Mediates the ubiquitination and proteasomal degradation of target proteins	F
NA	AA8G20050	NA	A(W-CAN1, W-CAN2, ES17)	2.97	NT	Unknown function	F
BIR1 (paralog2)	AA8G20060	At5g48380	A(W-CAN1, W-CAN2, ES17)	2.97	NT	Involved in negative regulation of plant immunity	F
LTP2	AA2G11850	At2g38530	A(W-CAN1, W-CAN2, ES17)	2.92	NT	May play a role in wax or cutin deposition in the cell walls	F
AKCS9	AA2G11860	At2g38530	A(W-CAN1, W-CAN2, ES17)	2.92	NT	Potential lipid transfer protein	F
NA	AA2G11870	NA	A(W-CAN1, W-CAN2, ES17)	2.92	NT	Unknown function	F
XBAT35	AA3G27210	At3g23280	A(W-CAN1, W-CAN2, ES17)	2.86	NT	Associated with defense and abiotic stress response	F
PRAF1	AA3G27220	At1g76950	A(W-CAN1, W-CAN2, ES17)	2.86	NT	Small transmembrane protein involved in vesicle trafficking	F
NA	AA3G27230	NA	A(W-CAN1, W-CAN2, ES17)	2.86	NT	Unknown function	F
SC5D	AA6G27580	At3g02580	T(W-CAN2)	17.43	NT	Involved in the biosynthesis of precursor of growth-promoting brassinosteroids	(C)E-CAN
BRR2C	AA8G47440	At5g61140	T(W-CAN2)	15.70	1.15*	Involved in spliceosome assembly, activation and disassembly	CE-CAN
PCR4	AA3G22360	At3g18460	T(W-CAN1)	14.63	0.96	May be involved in heavy metals transport	CE-CAN
CASP	AA3G22370	At3g18480	T(W-CAN1)	14.63	0.83	May be involved in intra-Golgi transport	CE-CAN
ILR2	AA3G22380	At5g08620	T(W-CAN1)	14.63	0.83	Probably involved in the metabolism of auxin conjugates	CE-CAN
ASPG1	AA3G22390	At5g08620	T(W-CAN1)	14.63	0.55	Aspartic protease involved in drought avoidance through abscisic acid signaling	CE-CAN
NA	AA7G12830	NA	T(W-CAN2)	14.57	NA	Unknown function	(C)E-CAN
SDN2	AA7G12840	At5g05540	T(W-CAN2)	14.57	NA	Regulates small RNAs turnover	(C)E-CAN
DET1 (paralog1)	AA7G12770	At4g10180	T(W-CAN2)	14.038	NA	Component of light signal transduction machinery	(C)E-CAN
DET1 (paralog2)	AA7G12780	At4g10180	T(W-CAN2)	14.038	NA	Component of light signal transduction machinery	CE-CAN
APX2	AA7G12790	At3g09640	T(W-CAN2)	14.038	NA	Plays a key role in hydrogen peroxide removal	CE-CAN
NA	AA7G12800	NA	T(W-CAN2)	14.038	NA	Unknown function	CE-CAN
NLE1	AA7G12810	At5g52820	T(W-CAN2)	14.038	NA	Involved in growth and developmental regulation	CE-CAN
LAX2	AA6G21520	At2g21050	A(W-CAN1, W-CAN2)	7.82	NT	Mediates auxin uptake	CE-CAN
CSP4	AA6G21530	At2g21060	A(W-CAN1, W-CAN2)	7.82	NT	Chaperone that binds to and unwinds RNA	CE-CAN
VTL5	AA3G25050	At3g25190	A(W-CAN1, W-CAN2, ES17)	7.68	NT	Vacuolar iron transporter	E-CAN
NA	AA8G30270	NA	A(W-CAN1, W-CAN2, ES17)	7.60	NT	Unknown function	E-CAN
NA	AA8G30280	NA	A(W-CAN1, W-CAN2, ES17)	7.60	NT	Unknown function	E-CAN
MLP31	AA1G29520	At1g70840	A(W-CAN1, W-CAN2, ES17)	7.32	NT	Involved in defense and abiotic stress response	E-CAN
BSMT1 (paralog1)	AA1G29530	At3g11480	A(W-CAN1, W-CAN2, ES17)	7.32	NT	Catalyzes the methylation of salicylic acid	E-CAN
BSMT1 (paralog2)	AA1G29540	At3g11480	A(W-CAN1, W-CAN2, ES17)	7.32	NT	Catalyzes the methylation of salicylic acid	E-CAN
MSH4	AA4G18360	At3g11480	A(W-CAN1, W-CAN2, ES17)	7.30	NT	Promotes homologous recombination	E-CAN

Table S10: Top gene ontology (GO) categories associated with genes that overlapped the 0.5% upper tail of *3P-CLR* using different groups of branches (terminal or ancestral) and outgroups (F = French populations, CE-CAN = Central-Eastern Cantabrian populations, and E-CAN = Eastern Cantabrian populations). GO rank, GO ID, GO term, number of annotated genes, number of significant genes, significant by annotated ratio, expected ratio, p-value, and *A. alpina* gene list.

**GO enrichment analysis: terminal branches within Cantabria, with (C)E-CAN as the outgroup**								
**Rank**	**GO.ID**	**Term**	**Annotated**	**Significant**	**Ratio**	**Expected**	**P-value**	***A. alpina* IDs**
1	1902476	chloride transmembrane transport	8	3	0.375	0.10	0.000120	AA8G27720, AA1G19000, AA1G19050
2	0035652	clathrin-coated vesicle cargo loading	2	2	1.000	0.030	0.000170	AA8G32590, AA3G19790, AA3G19790
3	0006582	melanin metabolic process	3	2	0.667	0.04	0.000500	AA3G29310, AA3G28530
4	0045921	positive regulation of exocytosis	32	4	0.125	0.42	0.000750	AA3G28530, AA7G06300
5	0001676	long-chain fatty acid metabolic process	2	2	1.000	0.03	0.000170	AA3G28530, AA4G07770, AA4G07780, AA4G25990
6	0043162	ubiquitin-dependent protein catabolic process	15	3	0.200	0.19	0.000880	AA3G29360, AA3G22810, AA3G18860
7	0050848	regulation of calcium-mediated signaling	4	2	0.500	0.050	0.000990	AA3G28530, AA7G12960
8	0000381	regulation of alternative mRNA splicing, via spliceosome	19	3	0.158	0.25	0.00179	AA4G18110, AA4G18130, AA2G07210
9	0051209	release of sequestered calcium ion into cytosol	7	2	0.286	0.090	0.003370	AA3G28530, AA7G12960
10	1901019	regulation of calcium ion transmembrane transport	7	2	0.286	0.09	0.00337	AA3G28530, AA3G34510
11	0051345	positive regulation of hydrolase activity	2	2	0.060	1.090	0.004750	AA3G29310, AA3G22810, AA8G15710, AA8G32590, AA7G06300
**GO enrichment analysis: ancestral branch, with (C)E-CAN as the outgroup**								
**Rank**	**GO.ID**	**Term**	**Annotated**	**Significant**	**Ratio**	**Expected**	**P-value**	***A. alpina* IDs**
1	0071281	cellular response to iron ion	64	5	0.078	0.31	0.000015	AA3G24350, AA3G25020, AA3G25030, AA3G25040, AA3G25050
2	0071452	cellular response to singlet oxygen	5	2	0.400	0.02	0.000230	AA6G21120, AA8G19800
3	0010358	leaf shaping	12	2	0.167	0.06	0.001500	AA1G29450, AA5G19050
4	0031670	cellular response to nutrient	12	2	0.167	0.06	0.001500	AA6G21120, AA8G19800
5	0010075	regulation of meristem growth	59	3	0.051	0.29	0.002980	AA1G29450, AA5G19050, AA6G21120
6	0046283	anthocyanin-containing compound metabolic process	62	3	0.048	0.30	0.003430	AA3G29350, AA6G21120, AA8G30040
**GO enrichment analysis: terminal branches within Cantabria, with FR as the outgroup**								
**Rank**	**GO.ID**	**Term**	**Annotated**	**Significant**	**Ratio**	**Expected**	**P-value**	***A. alpina* IDs**
1	0042775	mitochondrial ATP synthesis coupled electron transport	36	5	0.1389	0.60	0.000320	AA8G18520, AA3G20490, AA6G12340. AA7G10960, AA5G06640
2	0006857	oligopeptide transport	78	6	0.077	1.31	0.00201	AA3G19710, AA4G25380, AA1G12910, AA8G14650, AA8G17860, AA8G31710
3	0009150	purine ribonucleotide metabolic process	462	17	0.037	7.76	0.002260	AA8G18520, AA1G15040, AA1G23770, AA1G35170, AA3G06510, AA3G20490<br>AA4G33220, AA6G12340, AA7G10960, AA3G19400, AA3G28870, AA3G20560<br>AA3G29930, AA5G06640, AA7G10920, AA7G11030, AA7G11250
4	0042538	hyperosmotic salinity response	84	6	0.071	1.41	0.002920	AA4G25380, AA7G11040, AA7G26010, AA7G26020, AA8G17870, AA2G15990
**GO enrichment analysis: ancestral branch, with FR as the outgroup**								
**Rank**	**GO.ID**	**Term**	**Annotated**	**Significant**	**Ratio**	**Expected**	**P-value**	***A. alpina* gene IDs**
1	0046580	negative regulation of Ras protein signal transduction	15	3	0.200	0.11	0.000150	AA1G15580, AA4G16250, AA4G16260
2	0051123	RNA polymerase II transcriptional preinitiation complex assembly	33	3	0.091	0.23	0.0016	AA1G22980, AA3G23040, AA3G23050
3	0045944	Positive regulation of transcription by RNA polymerase II	156	5	0.032	1.10	0.0016	AA3G23040, AA3G23050, AA4G16250, AA4G16260, AA5G21780

Table S11: Candidate genes associated with response to water deprivation and heat. Gene names, *A. alpina* gene ID, *A. thaliana* orthologs, branches on which signatures are detected (T = terminal, A = ancestral), maximum normalized CLR score, GO term, and GO ID.

Gene name	*A. alpina* ID	*A. thaliana* ortholog	Branch(es)	Normalized CLR (max)	GO term	GO ID
ASPG1	AA3G22390	At3g18490	T(W-CAN1)	14.63	response to water deprivation	0009414
NAC055	AA3G18870	At3g15500	T(W-CAN1), T(ES17)	11.02	response to water deprivation	0009414
PAL2	AA5G19770	At3g53260	T(W-CAN2)	8.90	response to water deprivation	0009414
RH26	AA8G10500	At5g08610	T(W-CAN1)	7.09	response to water deprivation	0009414
RH25	AA8G10510	At5g08620	T(W-CAN1)	7.09	response to water deprivation	0009414
PARG2	AA6G27810	At2g31865	T(ES17), T(W-CAN1)	6.94	response to water deprivation	0009414
ZHD11	AA2G15790	At1g69600	T(W-CAN2)	6.90	response to water deprivation	0009414
DRIPH	AA3G27540	At3g23060	T(W-CAN2)	6.39	response to water deprivation	0009414
ARR1	AA3G20460	At3g16857	T(W-CAN2)	6.22	response to water deprivation	0009414
RBG4	AA3G26460	At3g23830	T(W-CAN2)	6.03	response to water deprivation	0009414
HSP15.7	AA6G27830	At5g37670	T(ES17), T(W-CAN1)	6.55	response to heat	0009408
MYB94	AA5G09300	At3g47600	A(W-CAN2, ES17)	5.52	response to water deprivation	0009414
ASPG1	AA3G22390	At3g18490	A(W-CAN1, W-CAN2, ES17)	5.18	response to water deprivation	0009414
ERF011	AA5G15450	At3g50260	A(W-CAN1, W-CAN2, ES17)	5.18	response to water deprivation	0009414
Cpn10	AA1G29480	At1g14980	A(W-CAN1, W-CAN2, ES17)	6.55	response to heat	0009408
FKBP62	AA3G24960	At3g25230	A(W-CAN1, W-CAN2, ES17)	4.58	response to heat	0009408

Table S12: Candidate genes potentially related to vernalization. Gene names, *A. alpina* ID, *A. thaliana* orthologs, branches on which signatures are detected (T = terminal, A = ancestral), maximum normalized CLR score, GO term, and GO ID.

Gene name	*A. alpina* ID	*A. thaliana* ortholog	Branch(es)	Normalized CLR	GO term	GO ID
VIP4	AA8G47450	At5g61150	T(W-CAN2)	6.27	vernalization response	0042538
VIL1	AA3G25750	At3g24440	T(ES17)	5.00	vernalization response	0042538
FIP1	AA3G31360	At2g06005	T(W-CAN2)	11.17	protein binding	0005515

Table S13: GO categories enriched in the set of genes with significantly different $\theta_{\pi_n}/\theta_{\pi_s}$ compared to the genomic average difference between populations from Cantabrian Mountains and from the Alps (pvalue$<$ 0.005).

Rank	GO.ID	Term	Annotated	Significant	Ratio	Expected	P-value
1	GO:0019288	isopentenyl diphosphate biosynthetic process, methylerythritol 4-phosphate pathway	185	25	0.135	7.72	2.3e-07
2	GO:0016554	cytidine to uridine editing	181	23	0.127	7.56	2.1e-06
3	GO:0031426	polycistronic mRNA processing	174	22	0.126	7.27	3.7e-06
4	GO:0010588	cotyledon vascular tissue pattern formation	188	23	0.122	7.85	4.0e-06
5	GO:0031425	chloroplast RNA processing	188	23	0.122	7.85	4.0e-06
6	GO:0080156	mitochondrial mRNA modification	194	23	0.119	8.1	6.8e-06
7	GO:0010380	regulation of chlorophyll biosynthetic process	210	24	0.114	8.77	8.1e-06
8	GO:0019287	isopentenyl diphosphate synthesis, mevalonate pathway	184	22	0.120	7.68	9.3e-06
9	GO:0048564	photosystem I assembly	188	22	0.117	7.85	1.3e-05
10	GO:0006886	intracellular protein transport	568	46	0.08	23.72	1.5e-05
11	GO:0010207	photosystem II assembly	198	22	0.111	8.27	3.0e-05
12	GO:0010027	thylakoid membrane organization	213	23	0.108	8.89	3.1e-05
13	GO:0010305	leaf vascular tissue pattern formation	216	23	0.106	9.02	3.9e-05
14	GO:0010501	RNA secondary structure unwinding	74	12	0.162	3.09	5.3e-05
15	GO:0046686	response to cadmium ion	624	46	0.074	26.06	0.00015
16	GO:0009658	chloroplast organization	392	32	0.082	16.37	0.00025
17	GO:0010087	phloem or xylem histogenesis	283	25	0.089	11.82	0.00037
18	GO:0016125	sterol metabolic process	258	23	0.089	10.77	0.00054
19	GO:0050790	regulation of catalytic activity	724	49	0.068	30.23	0.00065
20	GO:0046822	regulation of nucleocytoplasmic transport	19	5	0.263	0.79	0.00090
21	GO:0015031	protein transport	729	62	0.085	30.44	0.00091
22	GO:0015833	peptide transport	534	38	0.071	22.3	0.0010
23	GO:0009225	nucleotide-sugar metabolic process	55	8	0.145	2.3	0.0019
24	GO:0006499	N-terminal protein myristoylation	689	45	0.065	28.77	0.0021
25	GO:0016310	phosphorylation	1466	90	0.061	61.21	0.0023
26	GO:0035556	intracellular signal transduction	971	59	0.061	40.55	0.0026
27	GO:1900865	chloroplast RNA modification	24	5	0.208	1.0	0.0028
28	GO:0006099	tricarboxylic acid cycle	35	6	0.171	1.46	0.0030
29	GO:0051302	regulation of cell division	101	11	0.109	4.22	0.0033
30	GO:0051274	beta-glucan biosynthetic process	74	9	0.122	3.09	0.0036
31	GO:0042273	ribosomal large subunit biogenesis	90	10	0.111	3.76	0.0043
32	GO:0006268	DNA unwinding involved in DNA replication	17	4	0.235	0.71	0.0047
33	GO:0006468	protein phosphorylation	1224	70	0.057	51.11	0.0047

Table S14: Candidate genes involved in vernalization and with different rate ratios of non-synonymous to synonymous substitutions or polymorphism, in populations from the Cantabrian Mountains and from the Alps. These genes were significantly different before, but not after FDR correction, therefore they lack a definitive signature of selection, and we mention them only as potential candidates. Rate ratios are compared to the genomic average, which is mostly influenced by demography. *A. alpina ID*, *gene name*, and *ID* show gene identifiers in *A. alpina* and *A. thaliana*. Shown are the number of derived non-synonymous (D_N) and synonymous substitutions (D_S), and their rate ratios (d_N/d_S), and the number of derived non-synonymous (P_N) and synonymous polymorphisms (P_S) and their rate ratios (p_N/p_S), in populations from the Cantabrian Mountains and from the Alps, sorted by p-value. The *logit coef* represents the logit coefficient from the GEE, *p-value* and *FDR p-value* are from the Wald test before and after correction.

	**Ortholog**		**Cantabrian Mountains**			**Alps**					
***A. alpina* ID**	**gene name**	**ID**	$\mathbf{D_N}$	$\mathbf{D_S}$	$\mathbf{d_N/d_S}$	$\mathbf{D_N}$	$\mathbf{D_S}$	$\mathbf{d_N/d_S}$	**logit coeff**	**p-value**	**FDR p-value**
AA4G15350	*UBP26*	At3g49600	34	12	0.786	39	11	0.983	-0.224	0.024	0.321
AA2G06190	*VIP5*	At1g61040	3	18	0.046	3	24	0.035	0.288	0.046	0.454
			$\mathbf{P_N}$	$\mathbf{P_S}$	$\mathbf{p_N/p_S}$	$\mathbf{P_N}$	$\mathbf{P_S}$	$\mathbf{p_N/p_S}$			
AA1G12650	*CDC5*	At1g09770	10	10	0.277	6	65	0.026	2.383	0.001	0.151
AA8G19580	*FRL1*	At5g16320	9	3	0.865	10	22	0.131	1.887	0.050	0.481

Table S15: Allelic variation at six non-synonymous mutations at *FRL1*, and the associated flowering behavior. In the top of the table, population frequencies of six non-synonymous mutations at *FRL1* in the Cantabrian Mountains and in France, and the flowering behavior of the corresponding populations. The proportion of missing genotype data is shown in brackets. Flowering behavior without vernalization for the corresponding populations (mentioned in brackets) from (Wunder et al. 2023) is categorized in early, late, and no onset of flowering. For the Cantabrian Mountains, we reported the range across populations (minimum-maximum). In the bottom of the table, the alleles at these same positions in seven related *Arabis* species and in *Arabidopsis thaliana*.

	**Allele frequency [%]**						**flowering behavior [%]**		
**Population(s)**	**12618926**	**12618944**	**12619056**	**12619062**	**12619091**	**12619412**	**early onset**	**late onset**	**no onset**
Cantabria	100.0	100.0	100.0	100.0	100.0	100.0	0-2	0-1	97-100
FR02(F2)	100.0	100.0	100.0	100.0	100.0	100.0	1	23	76
FR07(F7)	100.0	100.0	100.0	100.0 (9.0)	100.0	100.0	21	21	58
FR10(F6)	100.0	100.0	100.0	100.0	100.0	100.0	0	0	100
FR20	83.3	83.3	83.3	83.3	83.3 (33.3)	100.0 (67)	4	46	50
FR15	37.5	25.0	28.6 (12.5)	28.6 (12.5)	28.6 (12.5)	37.5	NA	NA	NA
FR08(F4)	0.0	0.0	0.0	1.4	1.4	1.4	58	3	39
FR01(F1a)	0.0	0.0	0.0	0.0	0.0	0.0	84	11	5
FR03(F1b)	0.0	0.0	0.0	0.0	0.0	0.0	14	44	42
	**Alleles**						**-**		
**Species**	**12618926**	**12618944**	**12619056**	**12619062**	**12619091**	**12619412**			
Arabis alpina Pajares	G	G	C	A	C	C			
Arabis alpina FR01-001	A	T	A	C	G	G			
Arabis montbretiana	A	T	A	C	G	G			
Arabis nordmanniana	A	G	A	A	G	G			
Arabis hirsuta	A	G	A	A	G	G			
Arabis nemorensis	A	G	A	A	G	G			
Arabis sagittata	A	G	A	A	G	G			
Arabis ciliata	A	G	A	A	G	G			
Arabis caerulea	A	G	A	A	G	G			
Arabidopsis thaliana	A	G	A	A	G	G			

Table S16: Summary statistics of the 11 consecutive genealogies that span the balanced FRL1 haplotype. Columns report the genomic coordinates and span of each genealogy, the number of mapped mutations, the time to the most recent common ancestor (TMRCA) and its empirical genome-wide upper quantile, the root balance (defined as the proportion of descendants in the smaller of the two root subtrees), the Colless index and its empirical genome-wide lower quantile, and the child/root ratio, defined as the mean length of the two branches descending from the root divided by the TMRCA. The last column reports the empirical upper quantile of the child/root ratio among trees with similar age (TMRCA between 100% and 105% of the focal tree) and similar root balance. The genealogy that overlaps five out of the six non-synonymous mutations is in the third to last row. The genealogy that overlap the sixth mutation is in the last row.

Chr	Start (bp)	End (bp)	Span (bp)	Mutations	TMRCA (kya)	TMRCA Q	Root balance	Colless	Colless Q	Child/Root	Child/Root Q[a]
chr8	12608955	12609812	858	14	716.0	0.455	0.358	3445	0.179	0.742	0.129
chr8	12609813	12610599	787	8	597.1	0.544	0.358	3155	0.083	0.803	0.014
chr8	12610600	12611076	477	10	713.9	0.457	0.358	2978	0.044	0.746	0.110
chr8	12611077	12613537	2461	32	726.5	0.448	0.360	3644	0.266	0.799	0.019
chr8	12613538	12615729	2192	26	786.0	0.407	0.384	3201	0.095	0.780	0.045
chr8	12615730	12617517	1788	32	755.4	0.428	0.384	3127	0.076	0.727	0.190
chr8	12617518	12617975	458	7	719.8	0.453	0.384	2772	0.018	0.780	0.040
chr8	12617976	12618738	763	12	754.5	0.428	0.384	2550	0.005	0.799	0.035
chr8	12618739	12619282	544	20	733.9	0.443	0.384	2933	0.037	0.778	0.046
chr8	12619283	12619295	13	2	670.2	0.489	0.395	3603	0.247	0.734	0.085
chr8	12619296	12620546	1251	22	670.6	0.489	0.384	3451	0.182	0.814	0.009

Supplementary Figures

Figure S1

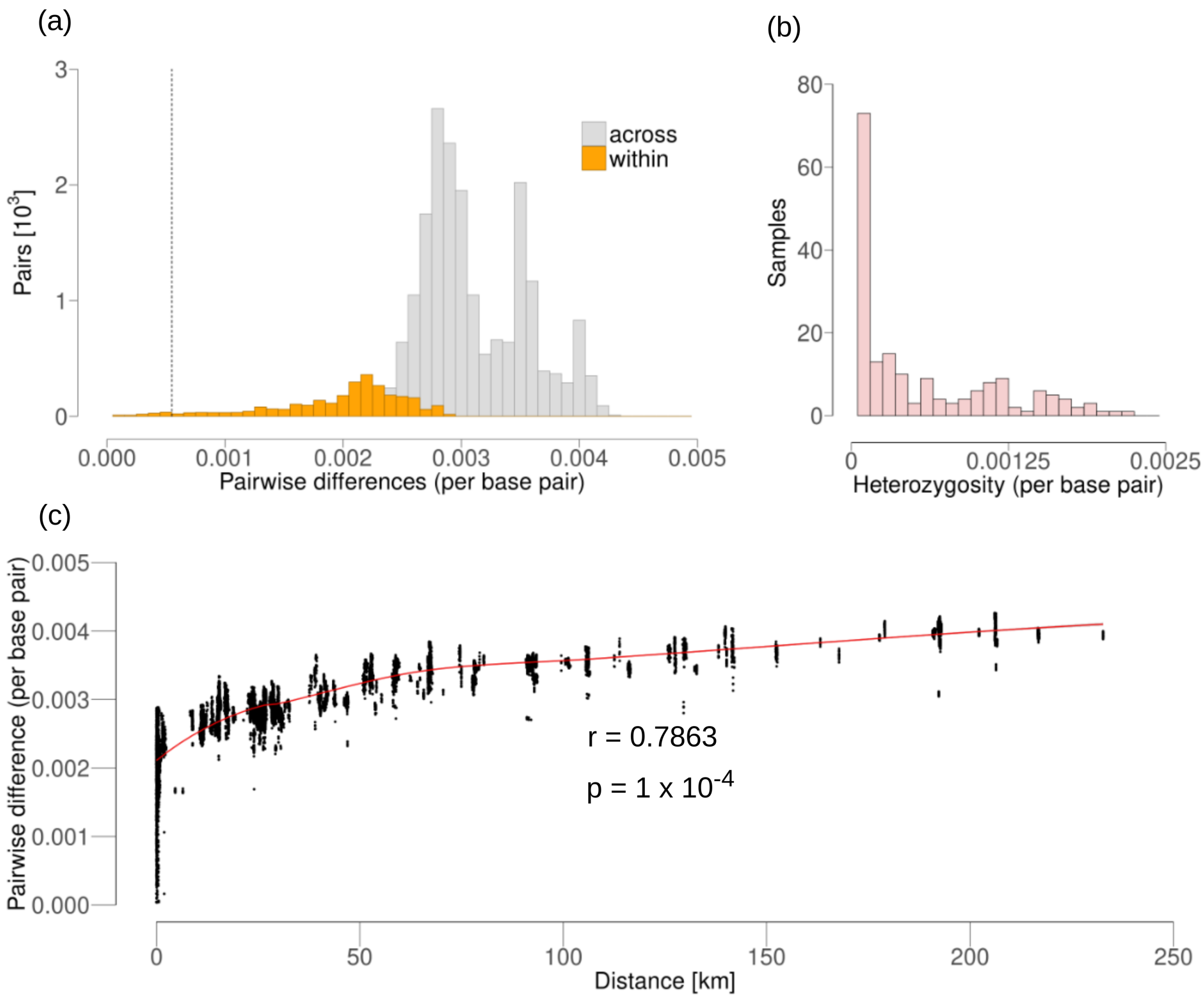

Distribution of genetic differences among pairs of individuals from the Cantabrian Mountains. **a:** Pairwise differences per base pair across (grey) and within (yellow) populations. The dashed line indicates the cutoff used to select unrelated samples. **b:** Distribution of heterozygote sites per base pair and per sample. **c:** Pairwise differences as a function of geographical distances. The figure shows the Mantel test correlation statistic (r) and p-value (p), and a local polynomial regression in red.

Figure S2

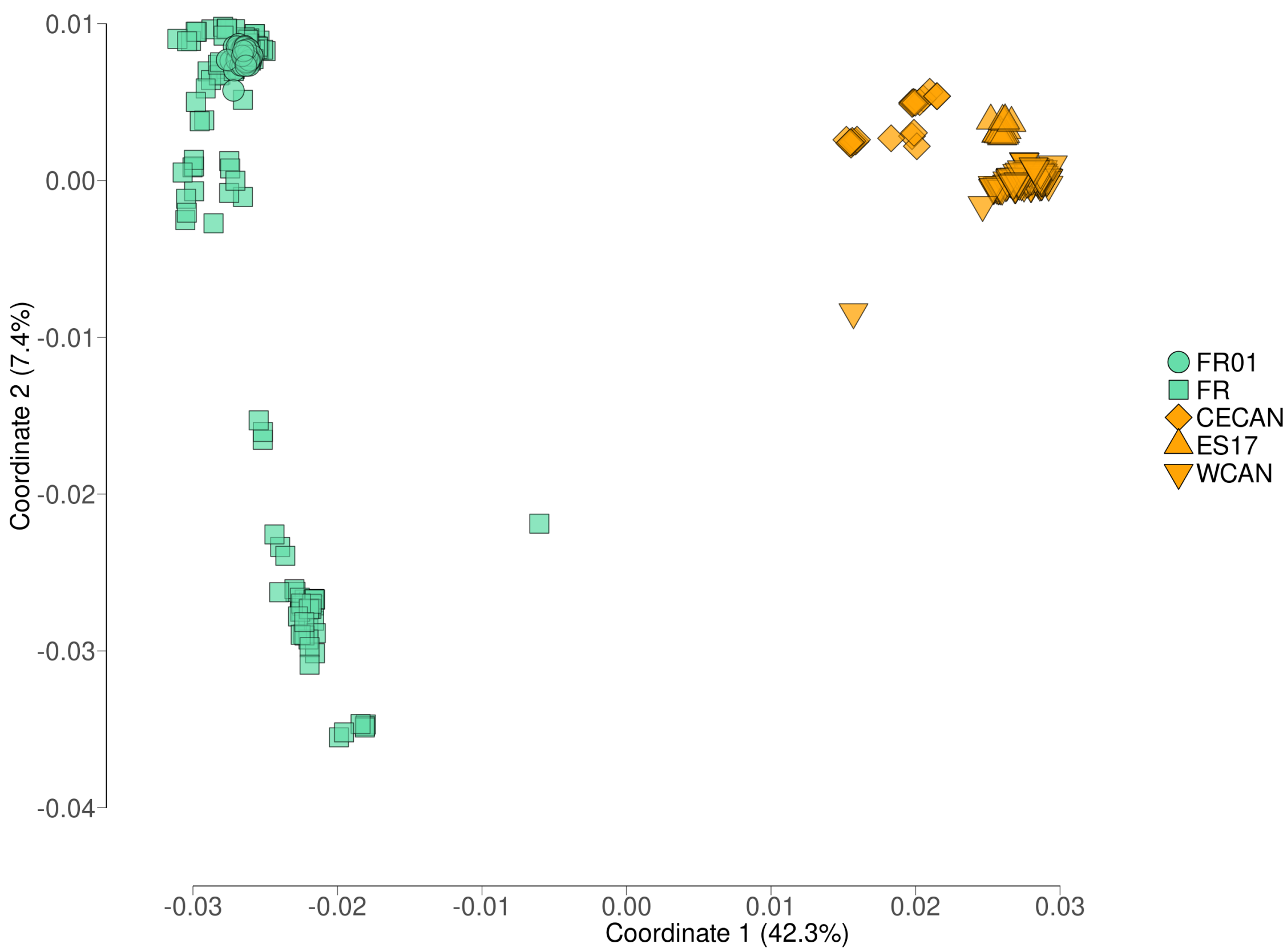

Principal coordinate analysis (PCoA) of accessions from Cantabrian Mountains and from the Alps based on genetic distances (1-IBS, identity by state). Axis labels indicate the percentage of variation explained by each axis. Colors denote the region of origin of samples, the Alps in green, and Cantabrian Mountains in orange. Among the samples from the Alps, shapes differentiate the FR01 population that was used as an outgroup in demographic modeling. Among the samples from the Cantabrian Mountains, shapes differentiate structure groups.

Figure S3

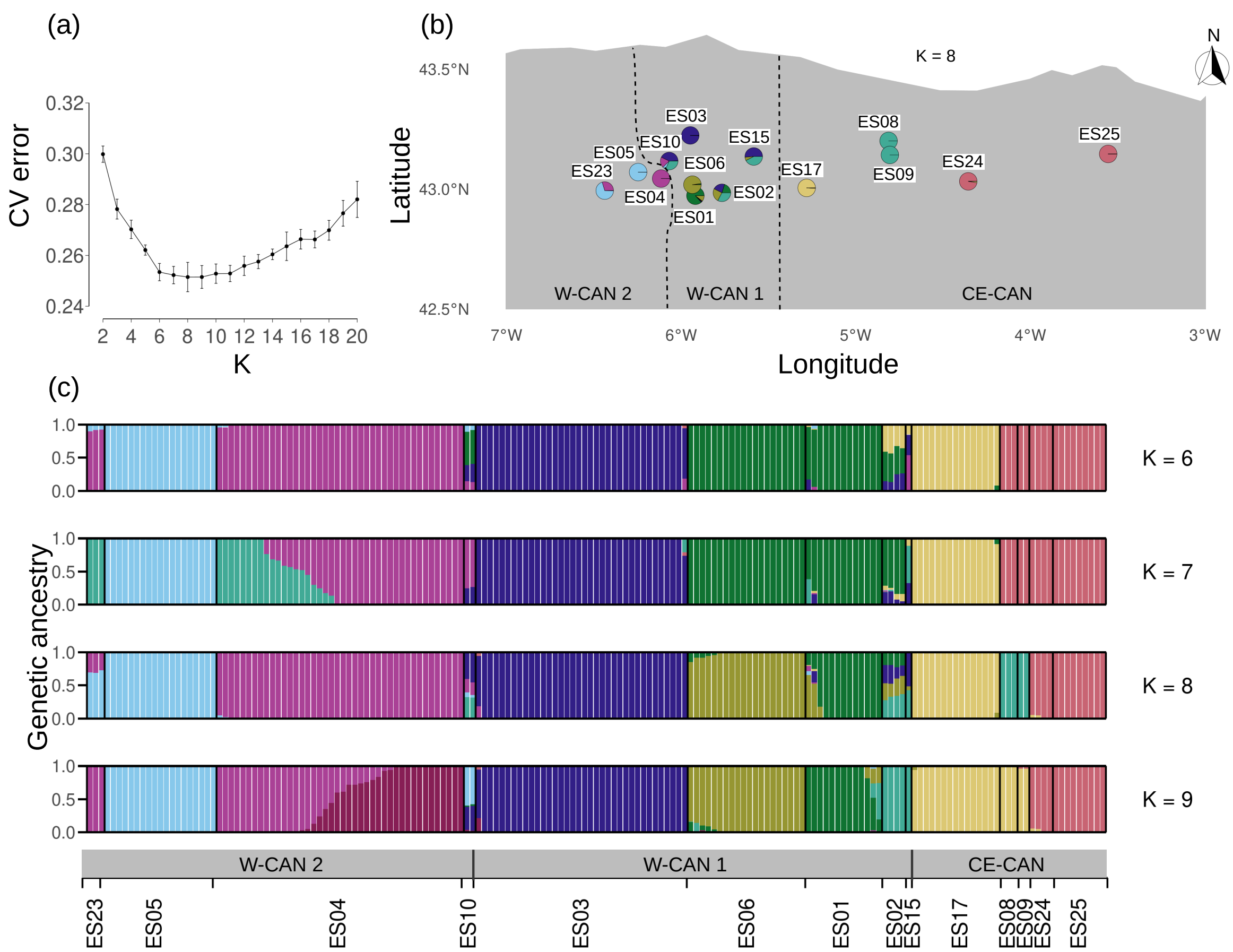

Genetic ancestry groups identified in Cantabrian Mountains *A. alpina* using ADMIXTURE. **a:** Mean cross-validation (CV) errors with two to 20 ancestry groups (K). Bars show standard deviation across 10 replicates. **b:** Geographical distribution of ancestry groups inferred from the run with the lowest average CV error ($K = 8$) across replicates. Pie charts represent populations and colors represent ancestry groups. **c:**. Genetic ancestry proportions per individual for runs using six to nine ancestry groups.

Figure S4

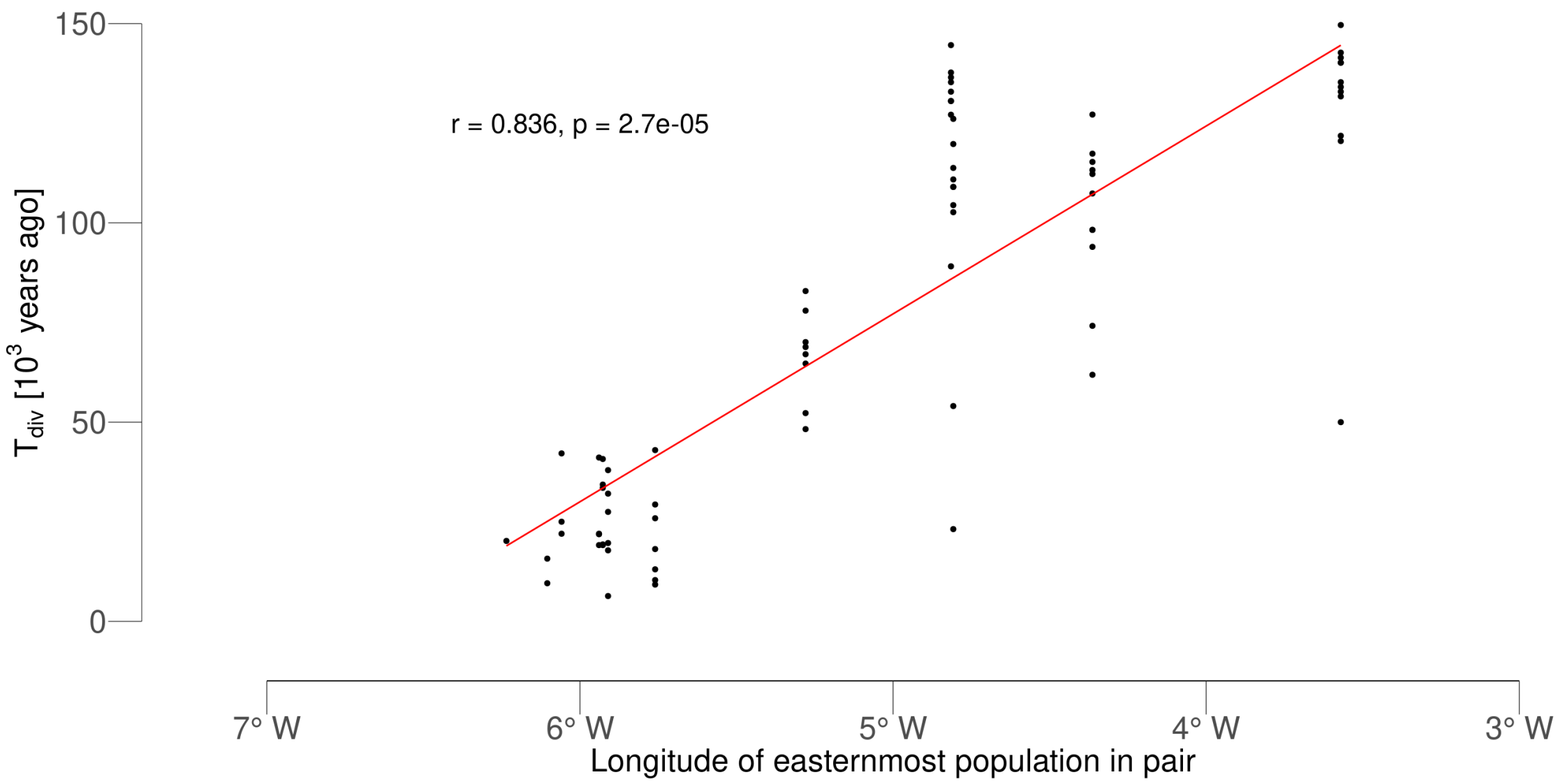

Pairwise split times between populations from the Cantabrian Mountains as a function of the longitude of the easternmost population in each pair. The red line represents a linear fit to this relationship. The Pearson correlation coefficient (r) and empirical p-value based on 10^6 permutations of the population coordinates (p) are shown within the figure.

Figure S5

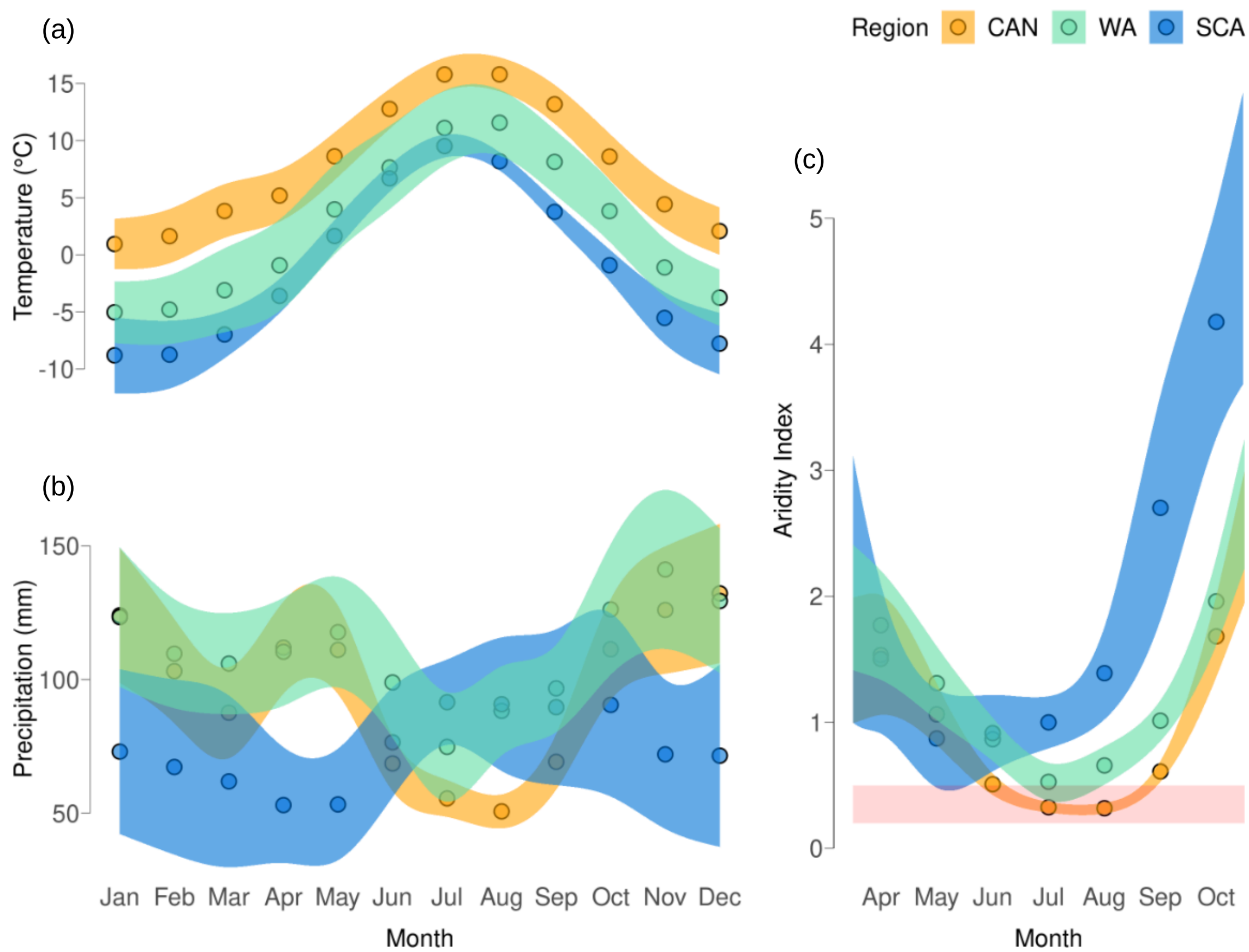

Yearly course of **a:** temperature, **b:** precipitation and **c:** aridity index (AI) across European populations from the Cantabrian Mountains (CAN), Western Alps (WA), and Scandinavia (SCA). Monthly averages across populations are indicated by points, and standard deviations are shown by smoothed ribbons. The red shaded area in (c) indicates semi-arid conditions defined as $0.2 \leq AI \leq 0.5$. Note that lower values for AI correspond to more arid climates that higher values.

Figure S6

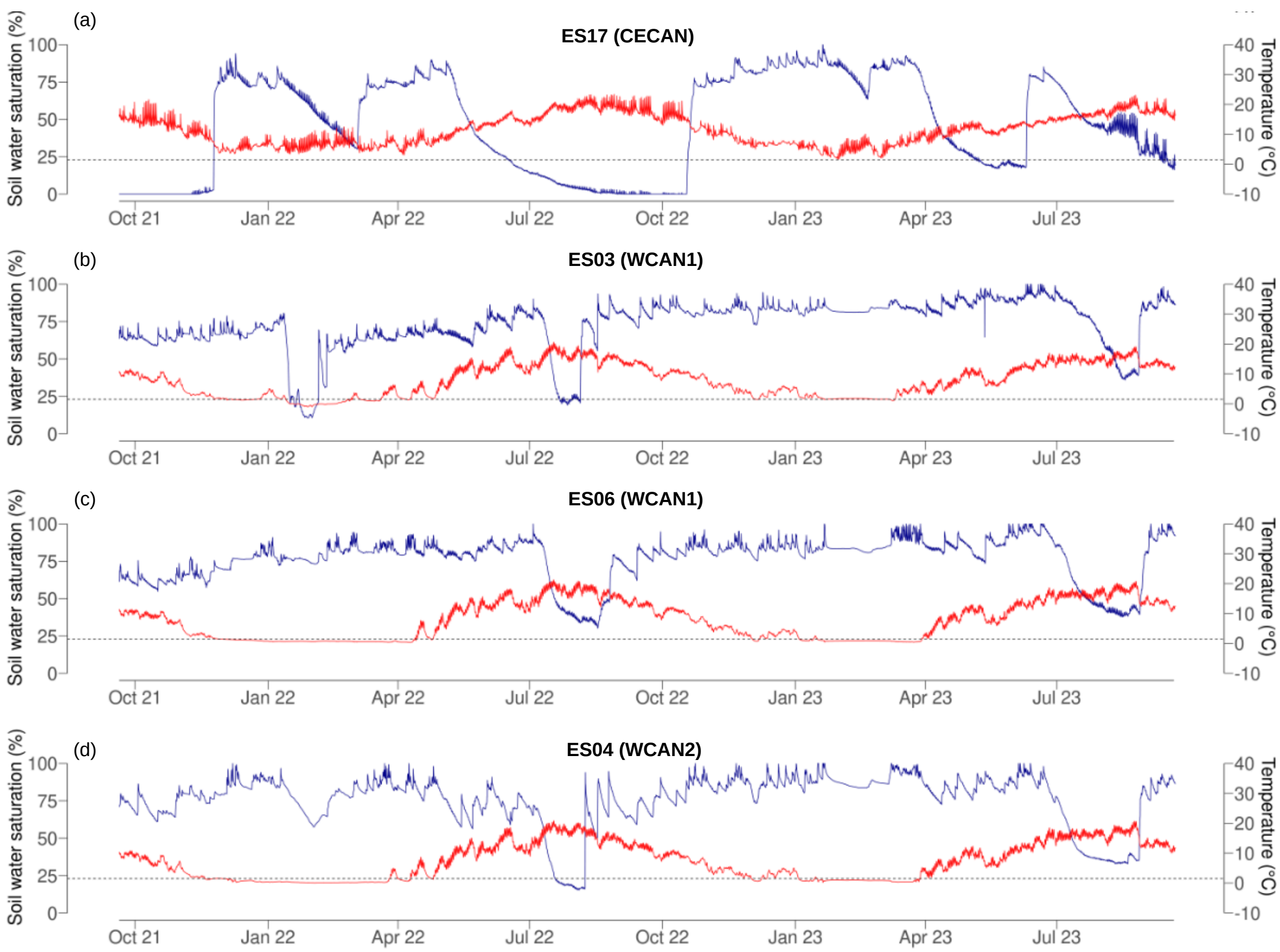

Soil water saturation (blue) and soil temperature (red) measured over a two-year period with soil moisture loggers that we installed at population sites ES17 (**a**), ES03 (**b**), ES06 (**c**), and ES04 (**d**) in the proximity of plants. The dotted line (23%) indicates the estimated wilting point for a silt loam soil, calculated from volumetric water content at wilting point and soil porosity following Saxton and Rawls (2006).

Figure S7

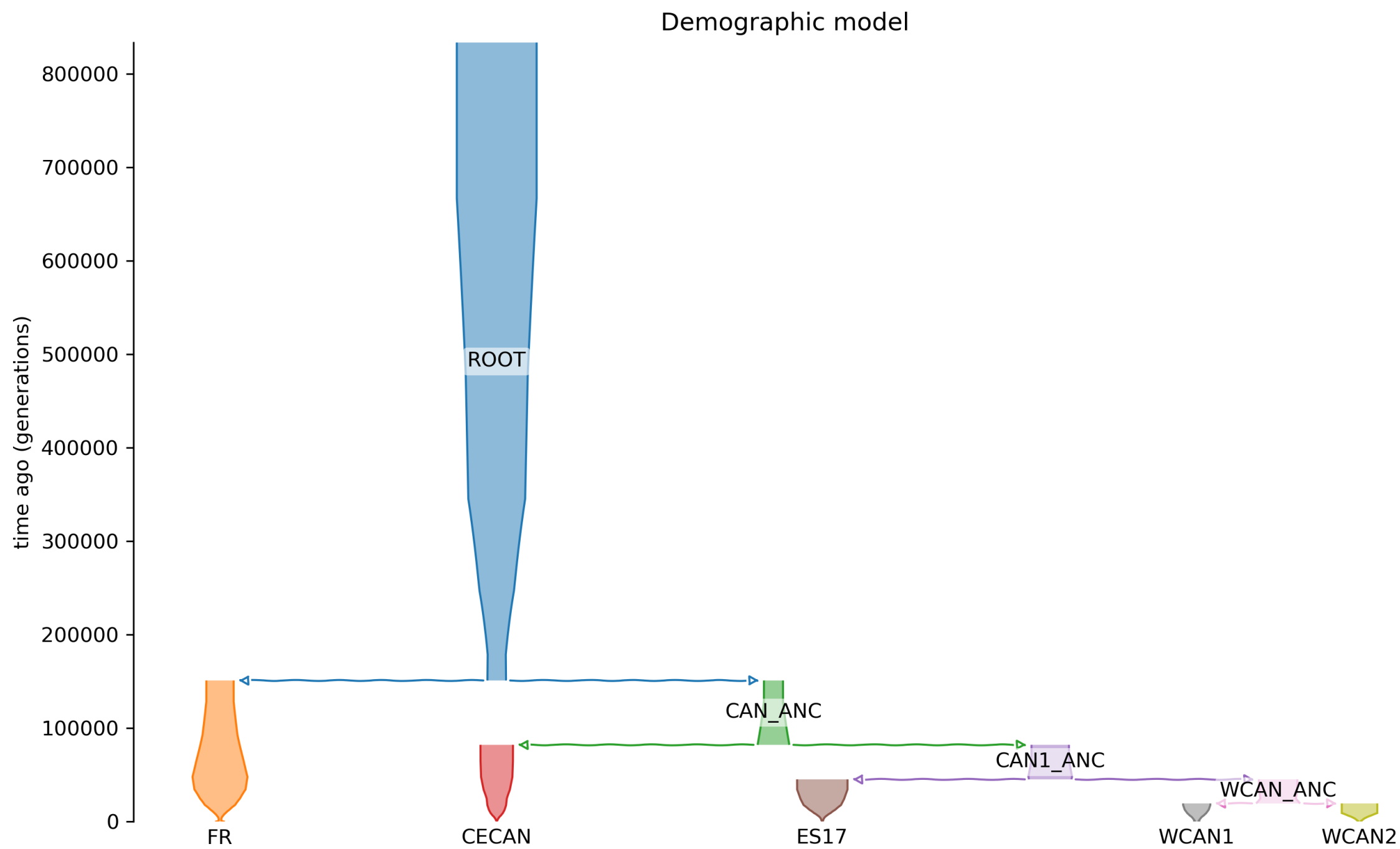

Demographic model used in coalescent simulations. Effective population sizes of the different groups are proportional to the width of the tubes. Population splits are indicated by horizontal arrows.

Figure S8

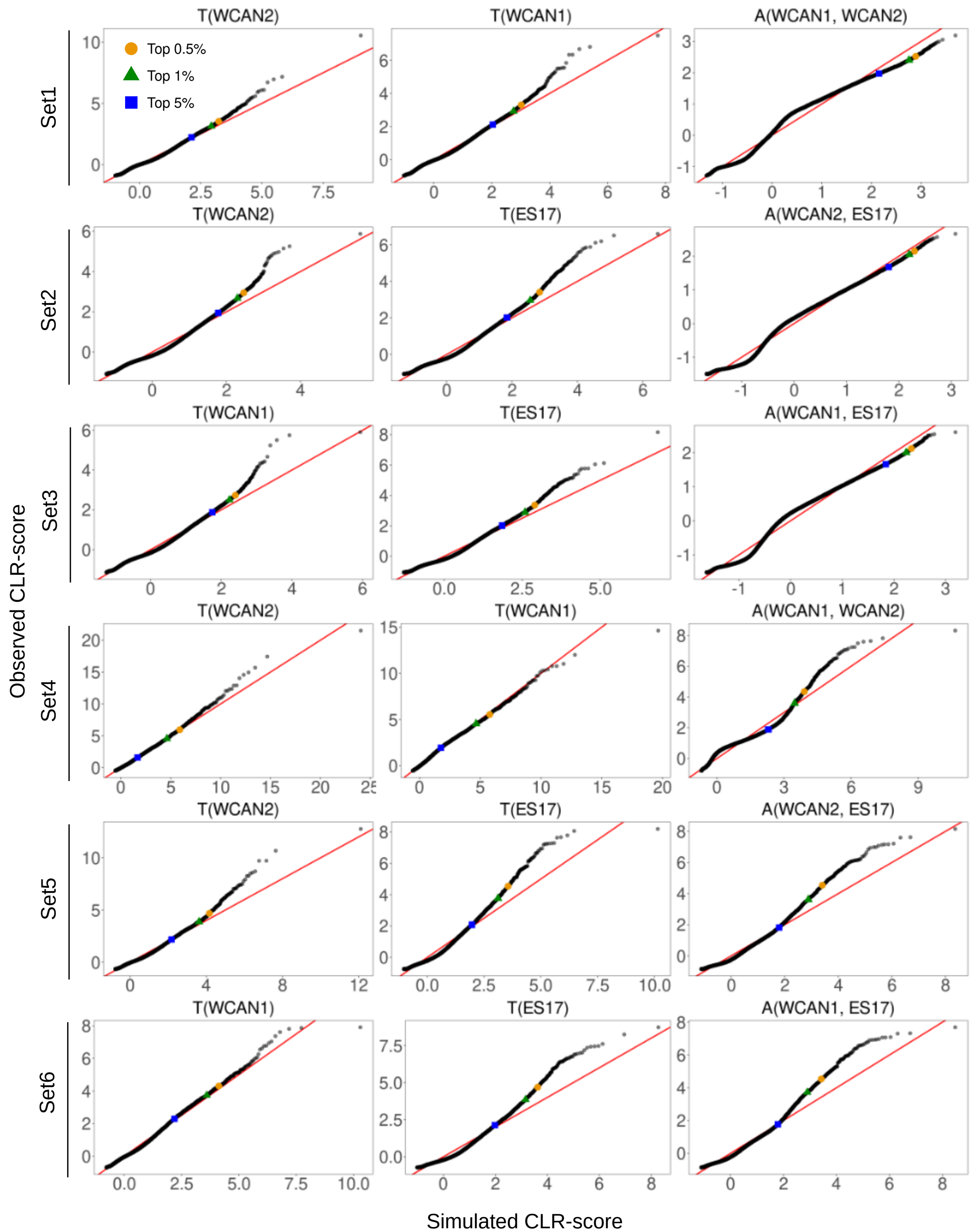

Comparison of quantiles between standardized simulated and observed CLR-scores from our empirical analysis. The red line indicates the 1:1 relationship. The upper 5%, 1%, and 0.5% quantiles are highlighted by blue squares, green triangles, and orange circles, respectively. Each *3P-CLR* setup is displayed in a separate row. Plot labels denote comparisons performed on terminal branches (T) or ancestral branches (A).

Figure S9

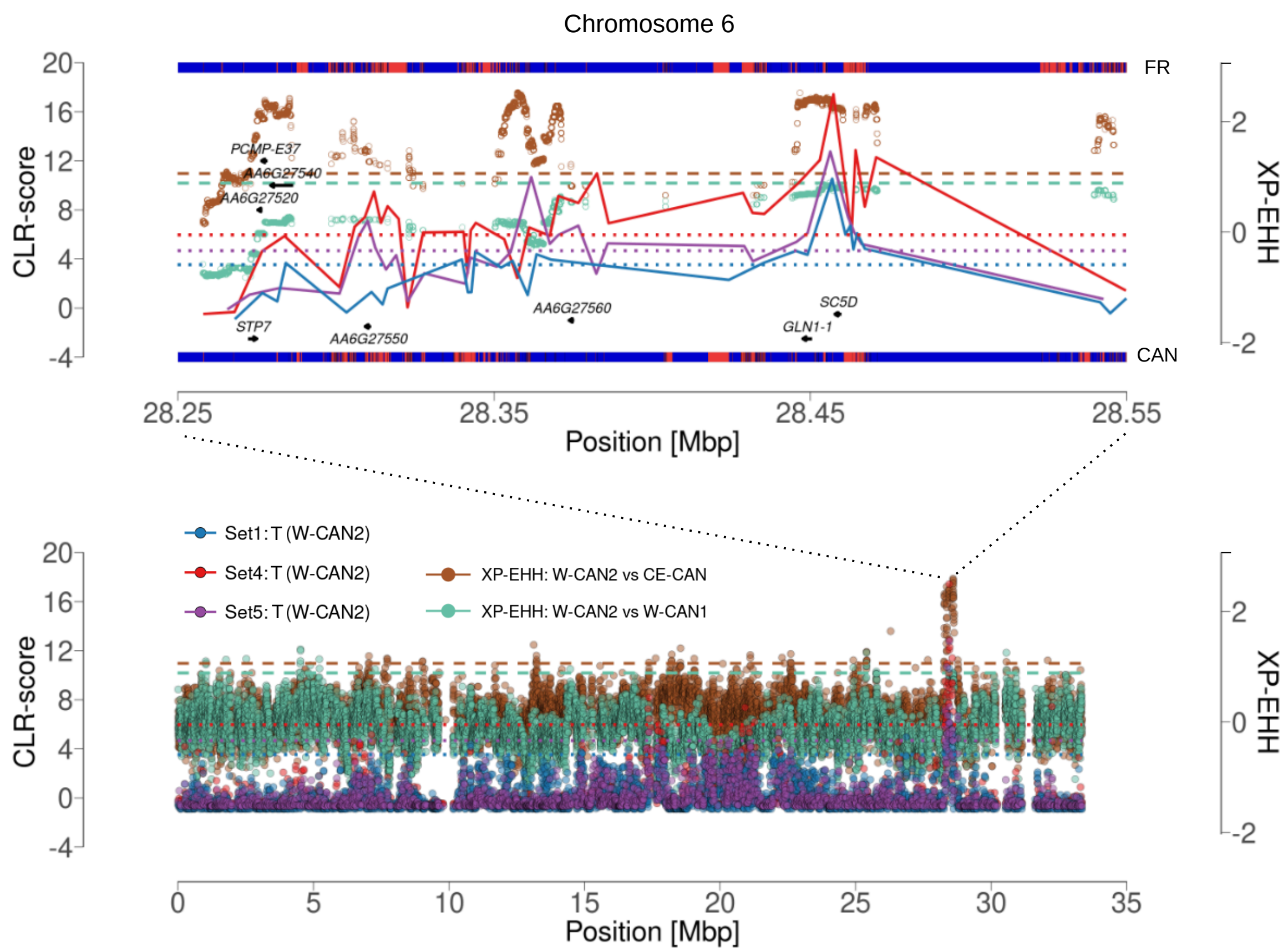

Standardized composite likelihood ratio scores (CLR-score - left y-axis) and cross population extended haplotype homozygosity (*XP-EHH* - right axis) scores at the *SC5D* locus, shown zoomed in (top) and across the chromosome (bottom). Genes are indicated by arrows and labeled with their *A. thaliana* ortholog or, if no ortholog is known, by their *A. alpina* gene identifier. Dotted lines show the 0.5% most extreme CLR scores for each analysis. Segments in the top panels show multicopy (red) and single-copy (blue) regions in France (top segment) and in the Cantabrian Mountains (bottom segment) inferred by ParaMask. The sets in the legend represent different combinations of populations (explained in Supplementary Table S7). CLR-scores for the terminal branch of W-CAN2 and for different group sets are shown in blue (Set1), red (Set4), and purple (Set5). *XP-EHH* scores are shown in brown (compared to CE-CAN) and light green (compared to W-CAN1).

Figure S10

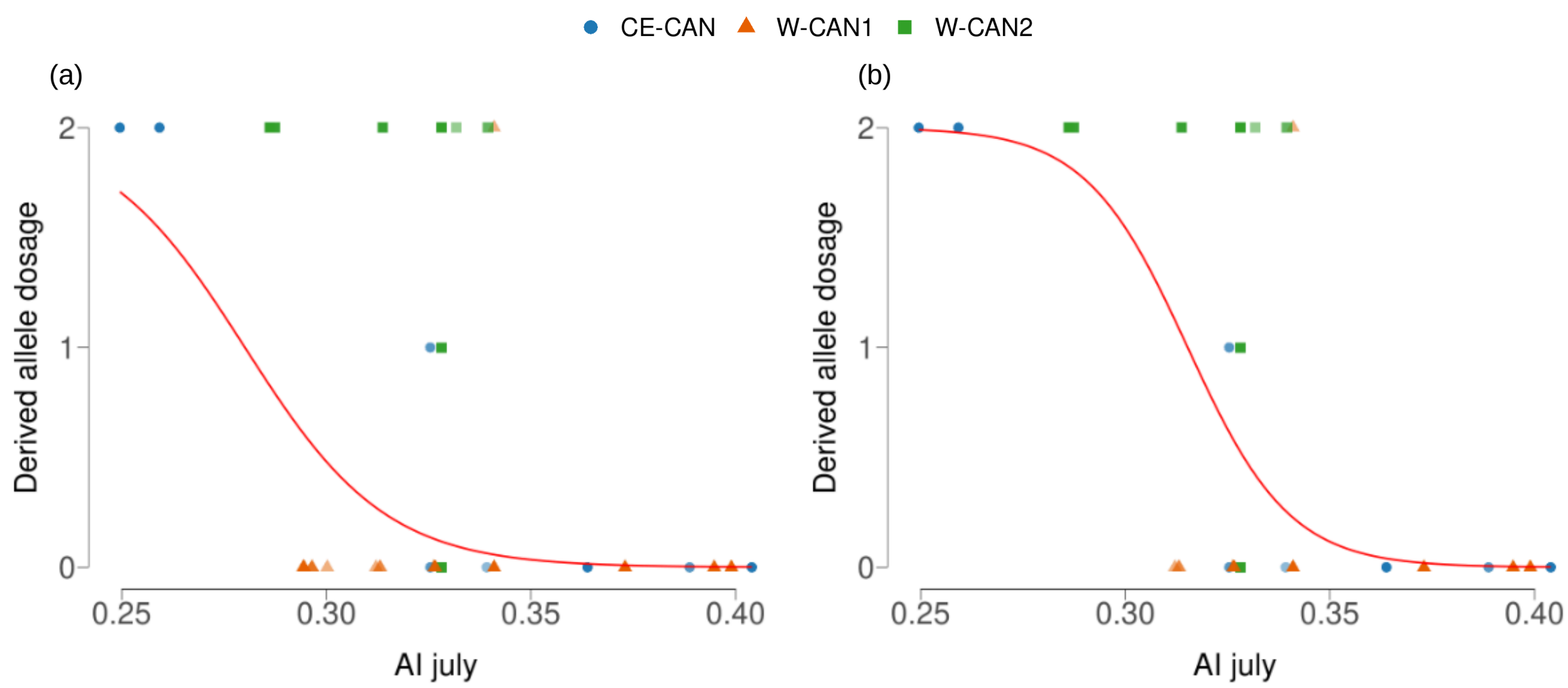

Binomial regression of the derived allele dosage at the 3bp insertion at *NAC055* and aridity index (AI) in July, with four principal coordinates from genome-wide structure analysis included as covariates. Data points represent single accessions, and they are shaped and colored by structure group. The red line shows the predicted relationship between allele dosage and AI. **a:** Binomial regression including all 211 accessions from the Cantabrian Mountains. **b:** Binomial regression excluding populations ES02, ES10, and ES15 (n = 200), which were collected at sites with micro climates very distinct from the surrounding climate (details in Supplementary Text).

Figure S11

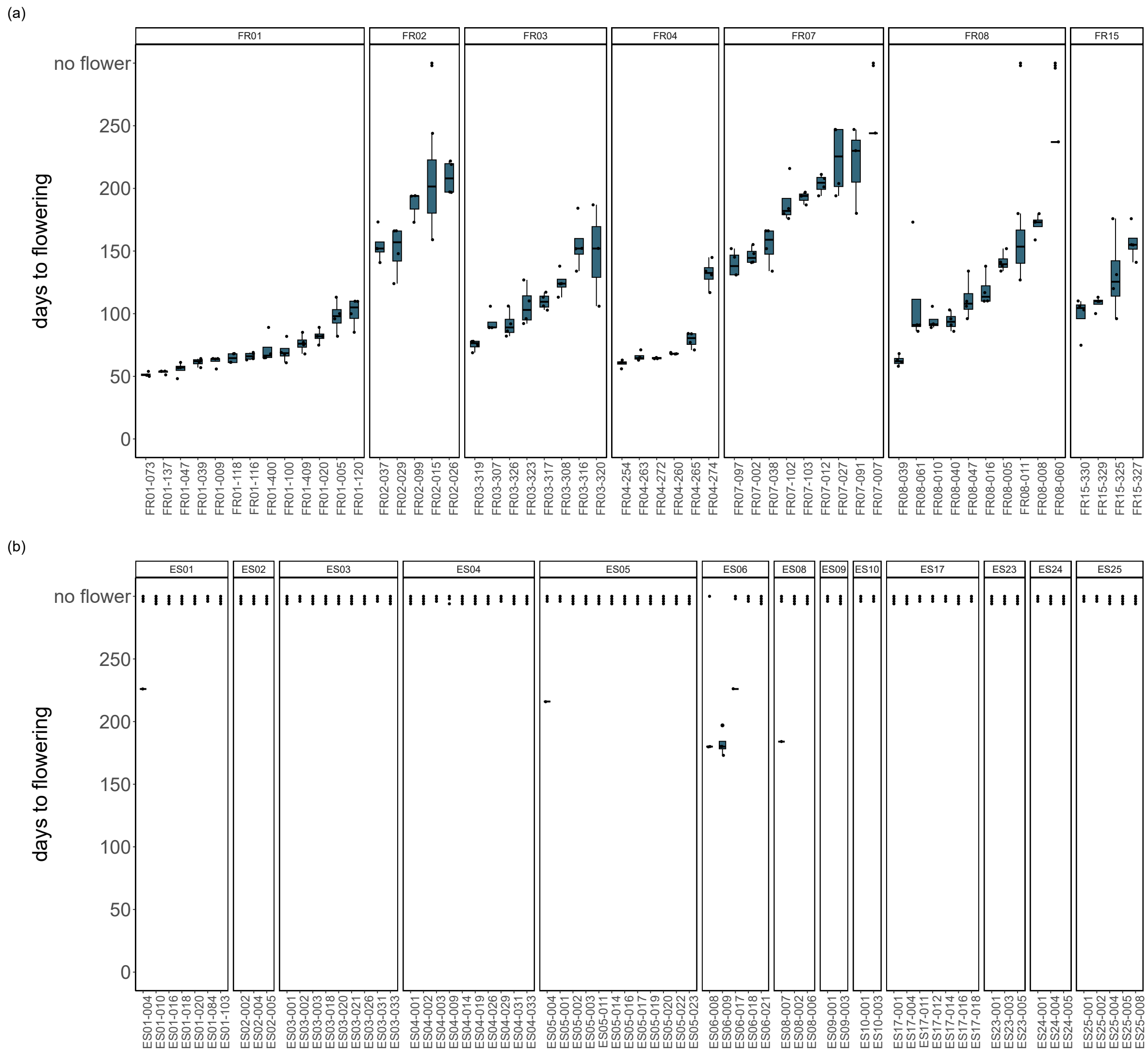

Time of flowering onset measured as the number of days between sowing and the opening of the first flower for accessions from (**a**) French populations and (**b**) Cantabrian Mountains populations grown in greenhouse conditions without vernalization.

Figure S12

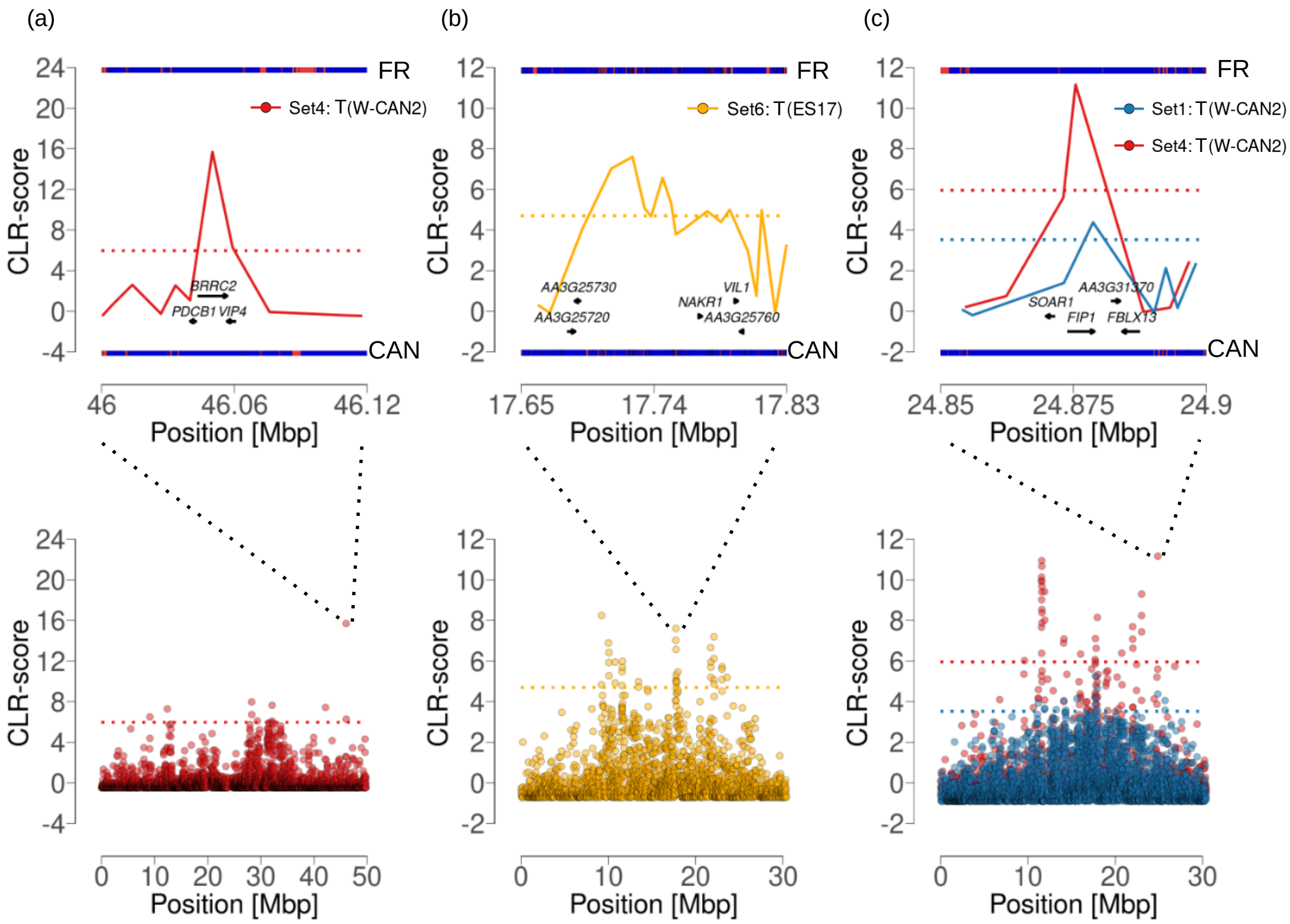

Standardized composite likelihood ratio scores for flowering genes loci *VIP4* (**a**), *VIL1* (**b**) and *FIP1* (**c**), shown zoomed in (top) and across the chromosome (bottom). Genes are indicated by arrows and labeled with their *A. thaliana* orthologs or, if no ortholog exists, by their *A. alpina* gene identifiers. The dotted lines show the 0.5% upper tail of CLR scores for each analysis. Segments in the top panels show multicopy (red) and single-copy (blue) regions in France (top segment) and in the Cantabrian Mountains (bottom segment) inferred by ParaMask. **a:** Scores for the terminal branch of W-CAN2 at the *VIP4* locus. **b:** Scores for the terminal branch of ES17 at the *VIL1* locus. **c:** Scores for the terminal branch of W-CAN2 and different *3P-CLR* analysis (blue - Set1; red - Set4) at the *FIP1* locus.

Figure S13

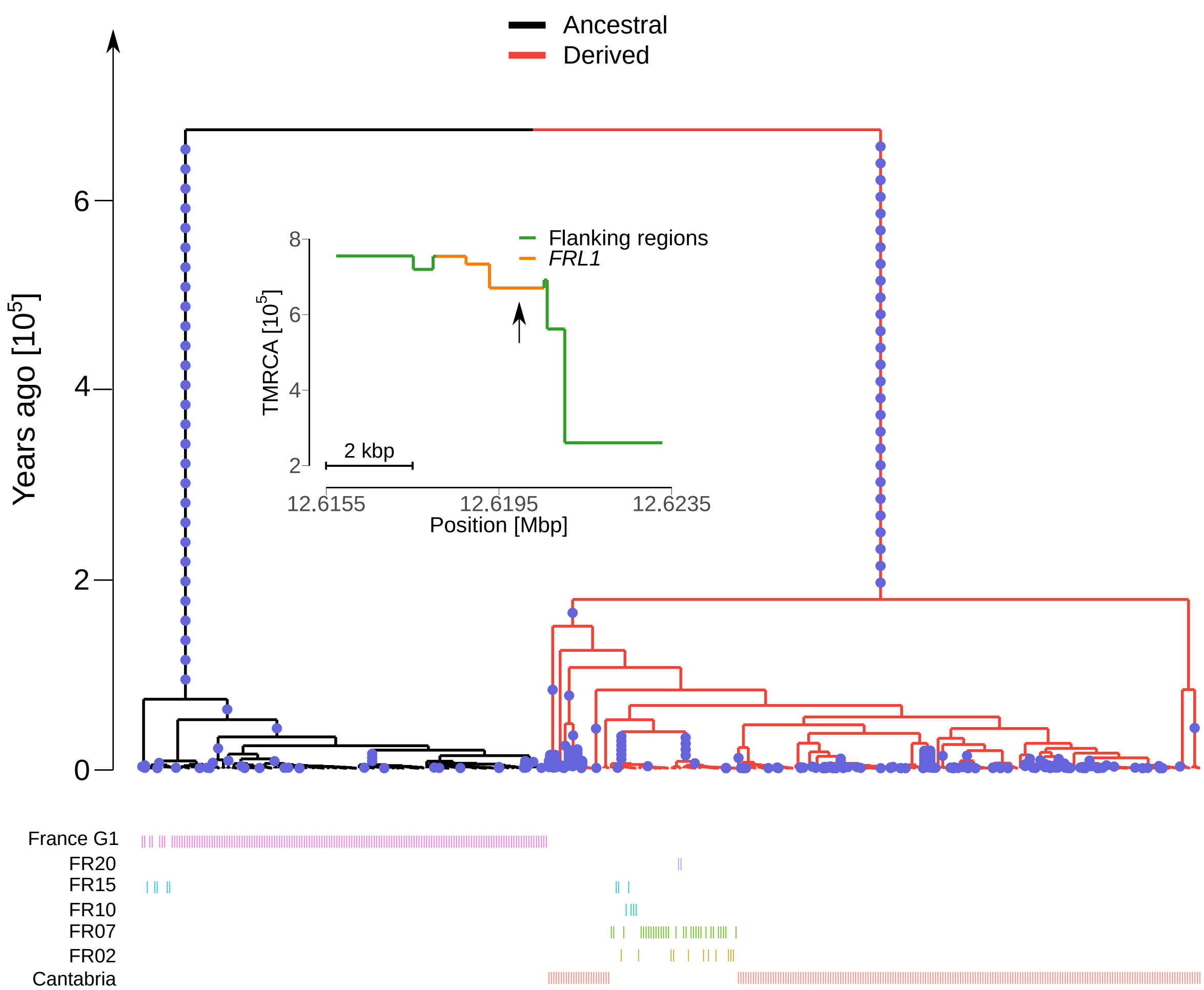

The genealogy at the *FRL1* locus that spans one of the six non-synonymous mutations. The genealogy that overlaps the remaining five mutations is shown in Fig. 5. Red branches indicate the lineages that carry the derived alleles at this mutation, and black branches represent lineages that carry the ancestral allele. France G1 (group 1) includes populations FR01, FR03, FR04, FR06, FR08, FR16, FR17, FR18, FR19, and FR21. The inset shows the time to most recent common ancestor (tmrca) distribution across genealogies at the *FRL1* locus. The tmrca of the genealogy depicted here is marked by an arrow.

Figure S14

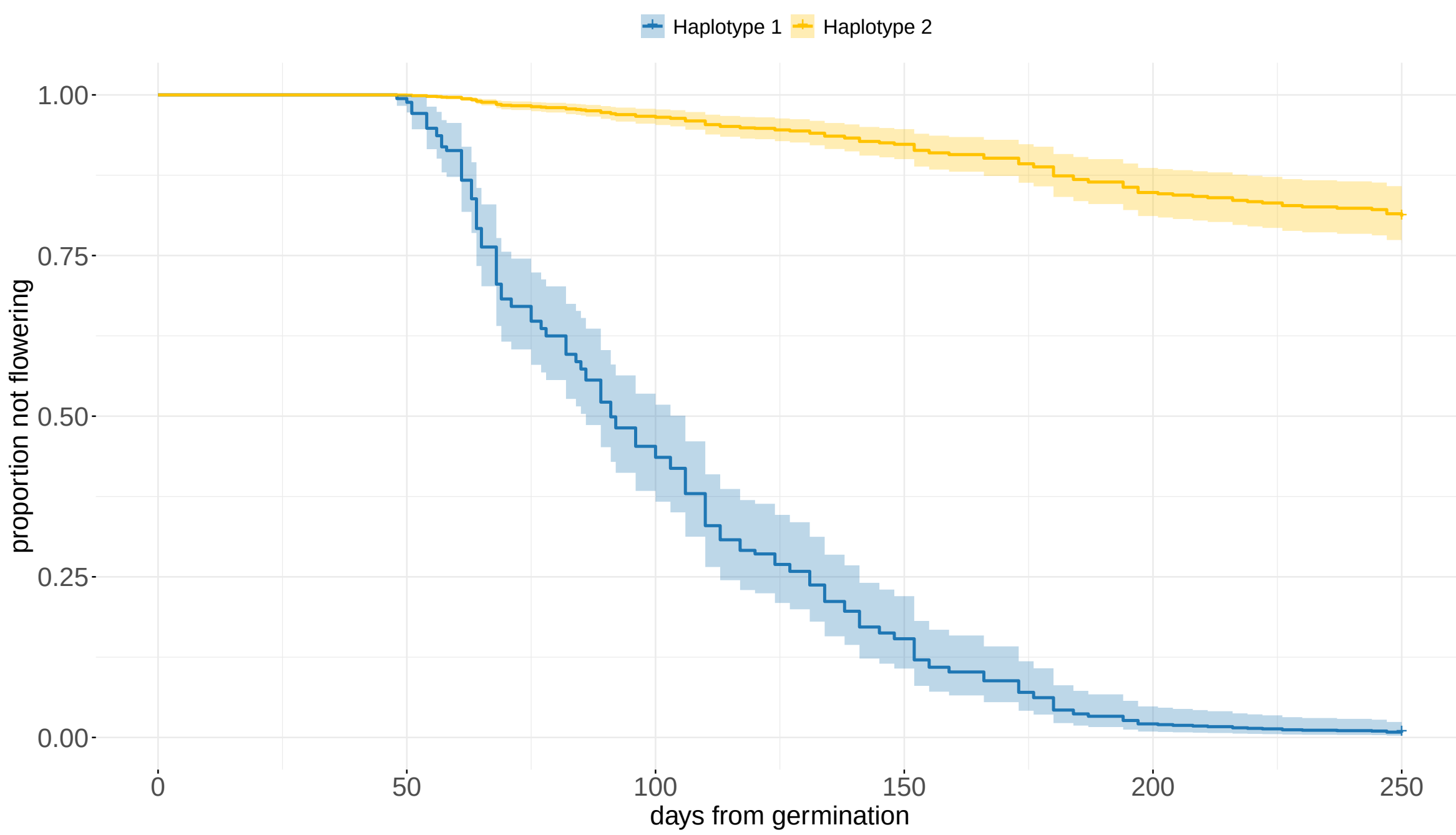

Proportion of non-flowering plants without vernalization fitted with a Cox proportional-hazards model for accessions from the Alps and from the Cantabrian Mountains. The blue curve represents individuals with the haplotype at *FRL1* that segregates at high population frequency in the Alps, and that is absent from the Cantabrian Mountains (haplotype 1). The yellow curve represents individuals with the haplotype at *FRL1* that is fixed derived in the populations from the Cantabrian Mountains, and segregating at low frequency in the Alps (haplotype 2).

References

Baumdicker F, Bisschop G, Goldstein D, Gower G, Ragsdale AP, Tsambos G, Zhu S, Eldon B, Ellerman EC, Galloway JG, et al. Efficient ancestry and mutation simulation with msprime 1.0. *Genetics*. 2022; 220(3):iyab229.

Behr AA, Liu KZ, Liu-Fang G, Nakka P, and Ramachandran S. pong: Fast analysis and visualization of latent clusters in population genetic data. *Bioinformatics*. 2016; 32(18):2817–2823. 10.1093/BIOINFORMATICS/BTW327.

Cantalapiedra CP, Hernandez-Plaza A, Letunic I, Bork P, and Huerta-Cepas J. eggNOG-mapper v2: Functional annotation, orthology assignments, and domain prediction at the metagenomic scale. *Molecular Biology and Evolution*. 2021; 38(12):5825–5829. 10.1093/MOLBEV/MSAB293.

Cantarel BL, Korf I, Robb SM, Parra G, Ross E, Moore B, Holt C, Alvarado AS, and Yandell M. MAKER: An easy-to-use annotation pipeline designed for emerging model organism genomes. *Genome Research*. 2008; 18(1):188. 10.1101/GR.6743907.

Cingolani P, Platts A, Wang LL, Coon M, Nguyen T, Wang L, Land SJ, Lu X, and Ruden DM. A program for annotating and predicting the effects of single nucleotide polymorphisms, SnpEff: SNPs in the genome of *Drosophila melanogaster* strain w1118; iso-2; iso-3. *Fly*. 2012; 6(2):80. 10.4161/FLY.19695.

Consortium TU. UniProt: the Universal Protein Knowledgebase in 2023. *Nucleic Acids Research*. 2023; 51(D1):D523–D531. 10.1093/nar/gkac1052.

Dainat J. AGAT: Another Gff Analysis Toolkit to handle annotations in any GTF/GFF format. `https://doi.org/10.5281/zenodo.3552717`. Version v1.0.0. Zenodo.

Delaneau O, Zagury JF, Robinson MR, Marchini JL, and Dermitzakis ET. Accurate, scalable and integrative haplotype estimation. *Nature Communications 2019 10:1*. 2019; 10(1):1–10. 10.1038/s41467-019-13225-y.

Fernandez UO, Scarpa S, Orgiazzi A, Panagos P, Van LM, Maréchal A, Jones A, et al. LUCAS 2018 soil module. 2022.

Flynn JM, Hubley R, Goubert C, Rosen J, Clark AG, Feschotte C, and Smit AF. RepeatModeler2 for automated genomic discovery of transposable element families. *Proceedings of*

the National Academy of Sciences of the United States of America. 2020; 117(17):9451–9457. 10.1073/PNAS.1921046117/SUPPL˙FILE/PNAS.1921046117.SAPP.PDF.

Gómez-Lende M, Serrano E, Jordá-Bordehore L, Sánchez-Benítez J, Millán JM, and Jordá-Bordehore R. High Mountain Mining Heritage of the Picos de Europa (Northern Spain). Las Gramas mining complex, conservation and potential use as a geotourism resource. *Geoheritage*. 2025; 17(3):106.

Gutiérrez-Valencia J, Fracassetti M, Horvath R, Laenen B, Désamore A, Drouzas AD, Friberg M, Kolář F, and Slotte T. Genomic signatures of sexual selection on pollen-expressed genes in *Arabis alpina*. *Molecular Biology and Evolution*. 2022; 39(1):msab349. 10.1093/molbev/msab349.

Harris RS. "Improved pairwise alignment of genomic DNA". PhD thesis. The Pennsylvania State University.

Holst F, Bolger A, Günther C, Maß J, Triesch S, Kindel F, Kiel N, Saadat N, Ebenhöh O, Usadel B, et al. Helixer–de novo prediction of primary eukaryotic gene models combining deep learning and a hidden markov model. *bioRxiv*. 2023:2023.02.06.527280. 10.1101/2023.02.06.527280.

Huerta-Cepas J, Szklarczyk D, Heller D, Hernández-Plaza A, Forslund SK, Cook H, Mende DR, Letunic I, Rattei T, Jensen LJ, et al. eggNOG 5.0: A hierarchical, functionally and phylogenetically annotated orthology resource based on 5090 organisms and 2502 viruses. *Nucleic acids research*. 2019; 47(D1):D309–D314. 10.1093/NAR/GKY1085.

Jiao WB, Accinelli GG, Hartwig B, Kiefer C, Baker D, Severing E, Willing EM, Piednoel M, Woetzel S, Madrid-Herrero E, et al. Improving and correcting the contiguity of long-read genome assemblies of three plant species using optical mapping and chromosome conformation capture data. *Genome Research*. 2017; 27(5):778–786. 10.1101/GR.213652.116.

Li H. Minimap2: pairwise alignment for nucleotide sequences. *Bioinformatics*. 2018; 34(18):3094–3100.

Martin M, Ebert P, and Marschall T. Read-based phasing and analysis of phased variants with WhatsHap. *Methods in molecular biology (Clifton, N.J.)* 2023; 2590:127–138. 10.1007/978-1-0716-2819-5˙8.

Morellón M, Morales-Molino C, Vegas J, Vera AV de, Plà-Rabes S, Leunda M, Sánchez-España J, Rodríguez JA, Arroyo X, and Mata MP. Reconstructing the environmental impact of mining on mountain lakes. *Science of the Total Environment*. 2025; 961:178382.

Nassar LR, Barber GP, Benet-Pagès A, Casper J, Clawson H, Diekhans M, Fischer C, Gonzalez JN, Hinrichs AS, Lee BT, et al. The UCSC Genome Browser database: 2023 update. *Nucleic Acids Research*. 2023; 51(D1):D1188. 10.1093/NAR/GKAC1072.

Nei M. Molecular evolutionary genetics. Columbia University Press10.7312/NEI-92038/HTML.

Nordborg M. Linkage disequilibrium, gene trees and selfing: An ancestral recombination graph with partial self-fertilization. *Genetics*. 2000; 154(2):923. 10.1093/GENETICS/154.2.923.

Osipova E, Hecker N, and Hiller M. RepeatFiller newly identifies megabases of aligning repetitive sequences and improves annotations of conserved non-exonic elements. *GigaScience*. 2019; 8(11):1–10. 10.1093/GIGASCIENCE/GIZ132.

Ossowski S, Schneeberger K, Lucas-Lledó JI, Warthmann N, Clark RM, Shaw RG, Weigel D, and Lynch M. The rate and molecular spectrum of spontaneous mutations in *Arabidopsis thaliana*. *Science (New York, N.Y.)* 2010; 327(5961):92–94. 10.1126/SCIENCE.1180677.

Racimo F. Testing for ancient selection using cross-population allele frequency differentiation. *Genetics*. 2016; 202(2):733–750. 10.1534/GENETICS.115.178095/-/DC1.

Saxton KE and Rawls WJ. Soil water characteristic estimates by texture and organic matter for hydrologic solutions. *Soil science society of America Journal*. 2006; 70(5):1569–1578.

Sharma V and Hiller M. Increased alignment sensitivity improves the usage of genome alignments for comparative gene annotation. *Nucleic Acids Research*. 2017; 45(14):8369–8377. 10.1093/NAR/GKX554.

Stiehler F, Steinborn M, Scholz S, Dey D, Weber AP, and Denton AK. Helixer: Cross-species gene annotation of large eukaryotic genomes using deep learning. *Bioinformatics*. 2021; 36(22-23):5291–5298. 10.1093/BIOINFORMATICS/BTAA1044.

Szpiech ZA and Hernandez RD. selscan: an efficient multithreaded program to perform EHH-based scans for positive selection. *Molecular biology and evolution*. 2014; 31(10):2824–2827.

Tjeng B, Arimond M, Bråten Grindeland H, Dalla Libera A, and Fulgione A. ParaMask: a new method to identify multicopy genomic regions, corrects major biases in whole-genome sequencing data. *Genome Biology*. 2025; 26(1):368. 10.1186/s13059-025-03836-8.

Toräng P, Vikström L, Wunder J, Wötzel S, Coupland G, and Ågren J. Evolution of the selfing syndrome: Anther orientation and herkogamy together determine reproductive assurance in a self-compatible plant. *Evolution; international journal of organic evolution*. 2017; 71(9):2206–2218. 10.1111/EVO.13308.

Wötzel S. "Molecular - genetic analysis of flowering time natural variation in *Arabis alpina* populations of the French Alps". PhD thesis.

Wunder J, Fulgione A, Toräng P, Wötzel S, Herzog M, Obeso JR, Kourmpetis Y, Van Ham R, Odong T, Bink M, et al. Adaptation of perennial flowering phenology across the European range of *Arabis alpina*. *Proceedings of the Royal Society B*. 2023; 290(2011). 10.1098/RSPB.2023.1401.

Zomer RJ, Xu J, and Trabucco A. Version 3 of the global aridity index and potential evapotranspiration database. *Scientific Data*. 2022; 9(1):409.
